# Research trends in the field of the gut-brain interaction: Functional dyspepsia in the spotlight – An integrated bibliometric and science mapping approach

**DOI:** 10.3389/fnins.2023.1109510

**Published:** 2023-03-08

**Authors:** Tai Zhang, Beihua Zhang, Xiangxue Ma, Jiaqi Zhang, Yuchen Wei, Fengyun Wang, Xudong Tang

**Affiliations:** ^1^Xiyuan Hospital, China Academy of Chinese Medical Sciences, Beijing, China; ^2^Department of Gastroenterology, Xiyuan Hospital, China Academy of Chinese Medical Sciences, Beijing, China; ^3^Institute of Digestive Diseases, Xiyuan Hospital, China Academy of Chinese Medical Sciences, Beijing, China

**Keywords:** functional dyspepsia, gut-brain axis, microbiota, eosinophil, mast cell

## Abstract

**Objectives:**

This study aims to perform a bibliometric analysis of functional dyspepsia (FD), which includes visualizing bibliographic information, in order to identify prevailing study themes, topics of interest, contributing journals, countries, institutions, and authors as well as co-citation patterns.

**Methods:**

The Web of Science™ Core Collection Database was used to retrieve all peer-reviewed scientific publications related to FD research. The validated search terms were entered into the “title” and “author keywords” fields, and the results were sorted by publication year from 2006 to 2022. There were no restrictions on language. On 12 February 2023, a manual export of the complete metadata for each original publication and review article was performed. CiteSpace was used to reveal co-authorship, publication, and co-citation patterns to find prominent authors, organizations, countries, and journals in FD research as well as to identify author keywords with strong citation bursts, which could indicate an emerging research area. VOSviewer was used to build the co-occurrence indicator (co-word) to identify the main author keywords on which previous studies focused and to induce clustered scientific landscape for two consecutive periods to identify intriguing areas for future research.

**Results:**

A search of the database retrieved 2,957 documents. There was a wave-like pattern in the number of publications until 2017, after which there was a spike in publication volume. The USA, China, and Japan provided the majority of contributions. In terms of institution, Mayo Clin, Univ Newcastle, and Katholieke Univ Leuven were found to be the prolific institutions. Additionally, the results indicate that eastern Asian researchers contributed significantly to the global knowledge of literature that led other countries; however, Canada, the USA, Australia, England, and Germany were found to have the highest degree of betweenness centrality. Nicholas J. Talley, Jan Tack, Gerald Holtmann, Michael Camilleri, Ken Haruma, and Paul Moayyedi occupied the top positions based on productivity and centrality indicators. Six thematic clusters emerged (*Helicobacter pylori* infection; pathophysiological mechanisms of FD; extraintestinal co-morbidities and overlap syndromes associated with FD; herbal medicine in FD; diabetic gastroparesis; and dietary factors in FD). “Acupuncture,” “duodenal eosinophilia,” “gut microbiota,” and others were among the author keywords with rising prevalence.

**Conclusion:**

In FD research, eastern Asian countries have established themselves as major contributors with the highest publishing productivity; however, research has primarily been driven by North America, Europe, and Australia, where cooperation is generally more active and highly influential scientific results are produced. Our analysis suggests that increased investments, training of human resources, improved infrastructures, and expanded collaborations are essential to improving the quality of FD research in Asia. The emerging author keyword analysis suggests that eosinophil-mast cell axis, gut microbiota, mental disorders, and acupuncture are the key areas that attract researchers’ attention as future research boulevards. There is a highly skewed distribution of research output across Asia, with most focus on complementary and alternative medicine (CAM) coming from Chinese, Japanese, and South Korean centers. However, CAM remains an underexplored area of research in the context of FD, and it deserves greater research efforts in order to obtain quality scientific evidence. Furthermore, we propose that the research framework of CAM should not be limited to dysmotility; rather, it could be interpreted within a more holistic context that includes the brain-gut-microbiota axis, as well as novel concepts such as duodenitis, increased mucosal permeability, and infiltration and activation of eosinophils and mast cells, among others. Overall, we provided bibliometrics-based overviews of relevant literature to researchers from different backgrounds and healthcare professionals to provide an in-depth overview of major trends in FD research.

## 1. Introduction

Functional dyspepsia (FD) typically manifests as epigastric pain or burning, postprandial fullness, and early satiety without any structural abnormalities on clinical or laboratory evaluation. Based on Rome IV criteria, three subtypes of the disease were identified: postprandial distress syndrome (PDS), epigastric pain syndrome (EPS), and mixed subgroups (Stanghellini et al., 2016). Although FD does not have a direct link to mortality or life-threatening consequences, its prevalence is high and greatly affects the quality of life (QOL) of those suffered (Mahadeva and Goh, 2006).

The pathophysiology of FD has yet to be fully understood in spite of its high prevalence and disease burden. The primary symptoms of FD are thought to be affected by a variety of complex interactions, including visceral hypersensitivity, abnormal gastric motility, *Helicobacter pylori* infection, and psychological stress (Kourikou et al., 2015; Enck et al., 2017; Talley, 2020).

If an FD diagnosis is made, *H. pylori* infection needs to be tested for and treated if present. Although some patients with FD (10–16%) may have symptomatic improvement following effective eradication therapy, only a small percentage will remain symptom-free in the long run, indicating that *H. pylori* is not the main culprit (Pellicano et al., 2016). FD patients may benefit from proton pump inhibitors (PPIs), prokinetic drugs, and antidepressants (Talley and Ford, 2015). In some cases, these medications do not provide satisfactory results, and a considerable number of FD patients seem to be resistant to conventional treatment. Further, some patients with FD display a low compliance with conventional therapy since they are concerned about the side effects of such pharmacological options (Freedberg et al., 2017; Quigley, 2017). PPIs, for example, are generally considered as safe and well tolerated, but when used long-term, there is a risk of developing *Clostridium difficile* infections, pneumonia, fractures, and acute interstitial nephritis (Wilhelm et al., 2013). Consequently, current treatment strategies for FD remain suboptimal.

On the one hand, there remain many aspects of uncertainty regarding FD, and the high scientific output suggests a continuing interest to address these unmet needs, but on the other hand, the exploding increase of research literature has necessitated the development of novel techniques to knowledge organization (Rodrigues et al., 2014). The ability to analyze large quantities of publications both macroscopically and microscopically, coupled with domain independence, classifies bibliometrics as such an approach (Tejasen, 2016). Pritchard (1969) describes bibliometrics as the use of mathematical and statistical approaches to books and other kinds of communication, whereas Hawkins (2001) defines it as the quantitative investigation of a body of literature’s bibliographic properties. Kokol et al. (2021a) demonstrated that the origins of bibliometrics date back to the late 19th century and that it has been widely and effectively utilized in medicine.

By using mathematics, bibliometric software, and statistical tools, bibliometrics makes it possible to conduct an analysis of the scientific literature production in a particular field (Blažun et al., 2015). This analysis can be used to determine (1) key research features such as literature output, productive research entities (i.e., countries, institutions, or authors), the degree of cooperation, or the spatial distribution of research; (2) content of the research literature production such as extensively researched themes, emerging areas of research, knowledge trajectory or influential literature; and (3) other relevant patterns of interest such as citation or co-citation. Thus, the main elements of a research topic can be found, arranged, and analyzed using bibliometric analyses. Moreover, it permits the identification of the most prolific agents in a field of study, be they authors, institutions, or countries; this may help in determining the players who are the primary motivators of a field of research. In the medical field, although bibliometric analysis does not contribute to the generation of new medical knowledge, it can facilitate the development of innovative meta-knowledge in certain medical fields, hence, expediting the production of new knowledge while charting future academic research agenda. Bibliometrics, alone or in combination with systematic, integrative, scoping reviews, and others, can be used to quantitatively evaluate the production of research literature (Šuran et al., 2022).

Blažun Vošner et al. (2017) and Kokol et al. (2021b) developed a novel synthetic knowledge synthesis methodology to solve the emerging complexity of knowledge synthesis and the concurrent new possibilities offered by digital presentation of scientific literature based on the triangulation of (1) distant reading, an approach for understanding the canons of literature not by close (manual) reading, but by using computer-based technologies, such as text mining and machine learning; (2) scientific mapping (also referred to as bibliometric mapping); and (3) content analysis. Such triangulation of technologies enables the quantitative and qualitative synthesis knowledge in a manner that extends traditional bibliometrics of publication metadata to understanding the patterns, structure, and content of publications *via* machine learning. In more detail, to comprehensively evaluate research activity in terms of research content and spatial entities, bibliometrics and knowledge synthesis are frequently used in all scientific fields; combining the two yields synthetic knowledge synthesis, a technique for analyzing large quantities of scientific publications (Blažun et al., 2015). Generally speaking, knowledge synthesis refers to the process of contextualizing and integrating the findings of individual research studies into a broader body of knowledge (Mallidou, 2014). Bibliometrics can therefore be utilized as a component of synthetic knowledge synthesis, which combines bibliometric mapping with content analysis (Kokol et al., 2022). In contrast to traditional and more formal knowledge synthesis techniques (such as meta-analysis, systematic reviews, literature reviews, etc.), which are typically manual, time-consuming, and limited to a small number of publications (typically fewer than 100 publications), synthetic knowledge synthesis permits the processing of tens of thousands of publications. Given its breadth, it can serve as a jumping-off point for researchers in quest of novel research avenues or areas that have been underappreciated. In addition, it offers a fresh or larger perspective on these research areas and facilitates the formulation of solutions to not-yet-solved barriers and challenges.

Some researchers have also proposed classifying the methods used in bibliometric analyses as either (1) performance analysis or (2) bibliometric mapping (Cobo et al., 2011; Swiontkowski et al., 2021). Performance analysis, a descriptive yet essential component of bibliometric studies, assesses the contributions and influence of research constituents (e.g., documents, authors, journals, universities, countries, etc.); in a different vein, bibliometric mapping takes a different tack by analyzing the interconnections between research constituents. A variety of bibliometric maps (also called landscapes) are used in bibliometric mapping to visually represent the volume and subject matter of the research literature (Begum et al., 2018). Through the use of proximity (x and y axes), color, size, and labeling, bibliometric mapping can accommodate a greater number of variables. Bibliometric mapping can be considered to be a form of “distant reading,” an approach that involves examining large volumes of written materials in order to uncover patterns. Generally, co-citations, co-authorships, bibliographic coupling, and co-words are typically utilized in bibliometric mapping analyses.

Performance analysis and scientific mapping can be combined in various bibliometric analysis software/tools to assess bibliographical evidence with validation. SALSA, PRISMA, CiteSpace, and VOSviewer are among the most widely used software applications in the scientific community (Baier-Fuentes et al., 2020).

Overall, the use of bibliometric data in science research can provide insight into the state of knowledge and emerging trends within a particular field or topic over time (Guler et al., 2016). Furthermore, bibliometric mapping analysis was applied to a wide range of topics, such as economics, social science, medical science, environmental studies, computer science, and artificial intelligence (Lin et al., 2021b; Chen et al., 2022b; He et al., 2022; Luo et al., 2022; Zhang L. et al., 2022a; Zhong and Lin, 2022).

Since bibliometrics involves the use of statistics to analyze published information (such as journal articles) and its associated metadata (such as titles, abstracts, and author keywords), it facilitates a greater understanding of what is valued, acknowledged, and utilized in scholarly literature in a particular field (Broadus, 1987; Albert et al., 2020). In the field of gastroenterology, several gastrointestinal diseases, including chronic atrophic gastritis (Zhang et al., 2022c), inflammatory bowel disease (Connelly et al., 2016; Schöffel et al., 2016, 2021; Azer and Azer, 2018; Luo et al., 2018; Chen et al., 2019; Chong et al., 2020; Li K. et al., 2022; Shen et al., 2022; Xiong et al., 2022; Xu et al., 2022), celiac disease (Narotsky et al., 2012; Demir and Comba, 2020), irritable bowel syndrome (IBS) (Zyoud et al., 2021; Zhang T. et al., 2022b), and gastrointestinal stromal tumors (Siddiq et al., 2018) have used bibliometrics to draw boundaries around their fields. Based on these parameters, journals, topics, members, and trends of the field have been described. Further, when narrowing down to functional gastrointestinal disorders (FGIDs), currently known as disorders of gut-brain interaction, several studies have only addressed IBS. In 2020, the prevalence of FGID in the populations of 33 nations was evaluated. Rome IV diagnostic questionnaire was used during the collection of data *via* Internet and personal interviews. In the study, prevalence of FD was 7.2 vs. 4.8%, and IBS was 4.1 vs. 1.5%, respectively (Sperber et al., 2021). FD was then designated as the most common gastroduodenal disorder. Despite the importance of the disorder in the field of FGIDs, the research topic is rife with traditional knowledge synthesis studies but deficient in comprehensive bibliometric analysis. In other words, no comprehensive performance analysis of scientific actors has been conducted, nor have potential research directions been sketched using a data-mining approach. Several studies have used the meta-analysis approach to study FD in terms of diagnosis, treatment, etiology, and epidemiology (Futagami et al., 2015; Ford et al., 2021; Gurusamy et al., 2021). The majority of these studies were aimed at gathering clinical evidence. In the face of the huge variety of studies, the variable quality of scientific research publications, and the vast amount of information available, investigators need to take a great deal of time assessing and selecting pertinent studies. To facilitate scientific research, it is consequently critical to categorize substantial, active, and evocative evidence retrieved from massive databases.

**FIGURE 1:**
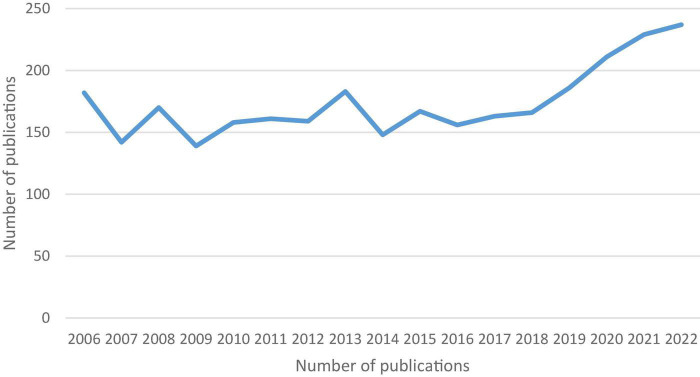
Publication output of FD research from 2006 to 2022.

In fact, the reason for the lack of a bibliometric analysis in the subject matter can be deduced from Figure 1, which reveals that the publication boom is only 4 years old. It was in 1992 that a group of experts convened in Rome with the aim of developing a classification system for FGIDs; consequently, the Rome criteria was developed for classifying gastrointestinal syndromes without any identifiable histopathologic, motility or anatomical abnormalities. This was followed by two additional revisions of the Rome criteria, each of which struggled with the term “functional.” However, “functional” is a vague and potentially stigmatizing term that has, over the years, generated great confusion and debate among professionals. Besides, subtle histopathologic and physiological abnormalities that previously go unrecognized indeed underlie some so-called functional disorders as evidence is updated. There has been a recent trend to modify the nomenclature to “disorders of gut-brain interaction” following the publication of the most recent and best validated Rome IV criteria in 2016 (Drossman and Hasler, 2016). As defined in Rome IV criteria, FGIDs are chronic gastrointestinal disorders closely associated with gut-brain interactions that may be accompanied by motility disturbances, visceral hypersensitivity, or microbial dysbiosis. Since the most recent version of the Rome criteria discourages the use of the term “functional” and suggests that the traditional FGIDs should be renamed “disorders of gut-brain interaction,” the researchers’ attention has shifted significantly toward the emerging “microbiota-gut-brain abnormalities” model and they have acknowledged the heterogeneous pathogenesis of FGIDs. Bibliometric analysis was earlier reasonably unattainable due to the scarcity of literature organized under the “disorders of gut-brain interaction” umbrella. However, research on intestinal microflora has been in full swing in recent years (Yuan et al., 2021), driving the publication boom that has altered the traditional literature’s dynamics. In light of these developments, a research gap has emerged due to the shifting patterns in FGIDs research, necessitating a comprehensive profile of research and identification of emerging trends in this field.

The first Rome classification system was published in 1990; in 1991, the gastroduodenal criteria divided FD into ulcer-like dyspepsia, dysmotility-like dyspepsia, reflux-like dyspepsia, or unspecified FD to take into account the fact that while some patients report pain as their cardinal symptom, others report postprandial symptoms, and many patients experience both types. This distinction was preserved in succeeding classifications, Rome I (1992) and Rome II (1999). Under Rome III (2006), FD was tightly defined, and the subtype labels were altered to EPS and PDS. This distinction was kept in the Rome IV (2016) classification (Stanghellini et al., 2016), which is responsible for some disparities in findings such as those of global epidemiological research. Overall, since 2006, two subsets of FD, PDS and EPS, were initially established by the Rome III consensus and, following the publishing of more supporting evidence, reaffirmed in the Rome IV edition. The time span of 2006–2022 feature two corpora for two successive time periods: 2006–2015 (Rome III) and 2016–2022 (Rome IV), with the latter period introducing the novel “altered gut-brain interactions” concept into this disorder. Hence, studies published after 2006 likely utilized the now accepted classification criteria and witnessed substantial resources invested to examine how the gut-brain axis has been affected in the pathophysiology of FD, resulting in an impressive corpus of scholarly work.

The present study deployed bibliometric analysis as a synthetic knowledge synthesis approach presented above to aggregate current evidence and review the spatial features, content and state of the art in FD research, with the goal of expanding the scope of existing studies and filling a gap in the literature by relating and analyzing FD-related publications and the impact of the authors, institutions, and countries over the past years. Our secondary goals were to assess potential future themes of interest in the discipline based on emerging trends.

In light of this, the following research questions are investigated using bibliometric and text mining analyses:

1. What are the dynamics and trends of FD research literature production in terms of document numbers and descriptive characteristics?
2. In terms of the number of publications, which authors, institutions, and countries are the most prolific?
3. How is the collaboration between the entities (i.e., authors, institutions, and nations) in the field of FD?
4. What are the most influential documents in the FD field?
5. What are the most prevalent author keywords and research themes, and how have they changed over time?
6. What associations exist among author keywords?
7. What are the general characteristics of FD research patterns in different geographical regions?
8. What are the emerging trends and future perspectives?

## 2. Materials and methods

### 2.1. Source of the data and search strategy

Data collection was conducted on 12 February 2023 by searching the Web of Science Core Collection (WOSCC). The WOSCC enables retrieval of complete bibliographic information (e.g., name list of authors and co-authors; institution, state, country; and complete reference lists) as well as information regarding citation activity relevant to the topic being researched. For the purpose of identifying FD-specific publications in the Science Citation Index-Expanded, a search strategy was developed (Supplementary material).

In order to acquire the full scientific output with specific and substantial content concerning FD, we have limited our search to original research articles, case reports, case series, and reviews that contained the search terms in the “title” and “author keywords.” In WOSCC, the “Topic” field corresponds to a search model which includes the words found in the title, abstract, author keywords, and *KeyWords Plus*. A key feature of *KeyWords Plus* is the ability to generate additional search terms based on article titles cited in bibliographies and footnotes in Thomson Institute for Scientific Information database which was once owned by Thomson Reuters, but now is maintained by Clarivate Analytics, and substantially augments indexing of title-words and author-keywords (Garfield and Sher, 1993). Consequently, documents found only by *KeyWords Plus* are more likely to be irrelevant to FD.

Furthermore, search terms mentioned in abstracts are, in most cases, marginal keywords and not essential components of the article itself, making these documents irrelevant to FD. In light of this, the authors chose to conduct a search based on “title” and “author keywords” rather than a search based on “TOPIC” since a “TOPIC” search would have identified the term in “*KeyWords Plus*” and “abstract,” resulting in an extensive list of publications that were not pertinent to the topic.

### 2.2. Data extraction

All data from the WOSCC database was downloaded in plain text format, including the names of the authors, the title of the paper, the name of the journal, the keywords used by the authors, and the abstract for each paper. Data errors were checked by entering all the data into a Microsoft Excel file.

To bring together the various names of an author, two researchers developed a standardization process. For further analysis, the combined dataset was transferred to CiteSpace and VOSviewer.

### 2.3. Data analysis

Bibliometric analysis was coupled with content analysis in this study as part of a two-tier analytic approach. Bibliometric analysis is one of the most widely used methods for determining the progress of a scientific work over time. Content analysis is a qualitative technique used by scholars to elicit information about a study’s findings and objectives (Williamson and Johanson, 2017). It is a widely accepted method for analyzing textual information by grouping it into more informative categories of data (Weber, 1990). In this study, the bibliometric stage examines the publication trend through time, influential parts of the literature, co-citation, and co-authorship. Besides, the content analysis was conducted in the second stage based on a thorough analysis of author keywords. On the basis of quantitative statistical analysis, content analysis is determined from keywords and abstracts in the literature. Specifically, keyword analysis is performed from author keywords, *KeyWords Plus*, and keyword extracted from abstracts using equation results from bibliometric analysis. Therefore, bibliometrics is heavily reliant on keyword analysis for tracing the development and progress of scientific fields. Furthermore, one of the most essential and extensively used tools for content analysis is the co-word analysis technique, which extracts topic-related terms from titles, keywords, and abstracts of publications to identify research emphasis and track research trends from various angles. In this study, the content analysis was conducted based on the author keywords cluster landscape, as previous research indicated that author keywords convey the author’s intended message to the research community in the most effective way (Železnik et al., 2017). The bibliometric analysis can be carried out with many well-developed tools, as discussed previously. Selecting bibliometric analysis tools should take into account their applicability and operability (Xu et al., 2021). VOSviewer and CiteSpace are two popular tools for quick bibliometric analysis since they are simpler to use than SALSA and PRISMA and do not necessitate programming knowledge (Aria and Cuccurullo, 2017). VOSviewer provides a robust mapping function, can manage massive datasets, and may be tailored to a variety of research requirements (Marshall et al., 2010). CiteSpace can produce dynamic timeline views and burst detection methods, facilitating inquiries into forecasts for the future of research and mutations in the behavior of hot topics (Kleinberg, 2002). Several investigations, especially those relating to publishing and citation trends, have utilized these tools simultaneously (Cunill et al., 2019; Alvarez-Peregrina et al., 2021; Brika et al., 2022; Sánchez-Tena et al., 2022). Comparing the differences between the two, VOSviewer is more accurate in its clustering algorithms; however, CiteSpace is superior in exhibiting evolution, has more attractive visuals, and is easier to interpret (Jia et al., 2022). This study use these two visualized analytic tools to illustrate the outcomes of the pertinent bibliographic records in order to create bibliometric maps and conduct content analysis.

Due to their individual strengths, each of these tools was used for a different purpose. CiteSpace 5.8.R3 was employed in this study for collaboration network analysis, co-citation analysis, and determining high-frequency author keywords. Using its clustering algorithms and data mining function, VOSviewer v1.6.10.0 was utilized to conduct co-occurrence analysis and build topical keywords clustering map to reveal hot topics. In addition, the use of different analytical approaches was intended to eliminate bias by complementing one another in a comprehensive, impartial, and responsible way. More specifically, the following techniques and software were used for data analysis:

(1) Overall publication performance: 1. publication output: CiteSpace 5.8.R3; 2. annual evolution of publications: Microsoft Excel 2019.
(2) Analysis of authors, institutions, and countries: 1. most prolific authors, institutions, and countries: CiteSpace 5.8.R3; 2. visualization of collaborative networks: CiteSpace 5.8.R3.
(3) Analysis of journals: CiteSpace 5.8.R3.
(4) Co-cited references and references with citation bursts: CiteSpace 5.8.R3.
(5) Analysis of author keywords: 1. co-occurring networks of author keywords: VOSviewer v1.6.10.0; 2. timeline visualization of co-occurrence author keywords network: CiteSpace 5.8.R3; 3. top author keywords with strong citation bursts: CiteSpace 5.8.R3; 4. top author keywords with the highest frequency: CiteSpace 5.8.R3. These two software are described in detail below.

CiteSpace is a free, Java-based tool that is available online (Chen, 2006a). It is a potent piece of software that employs betweenness centrality to identify the pivot points of literature and burstiness to quantify the sudden growth.

Betweenness centrality can be used to gauge a node’s potential for controlling communication. The goal of this method is to determine how often a point falls between pairs of other points along the shortest or geodesic paths between them (Abbasi et al., 2012); an element with a high betweenness centrality (>0.1) is considered pivotal (Fotopoulou et al., 2022). Betweenness centrality is defined in Eq. 1.


(1) $$C\,e\,n\,t\,r\,a\,l\,i\,t\,y\,\left(n\,o\,d\,e\,i\right)=\sum_{i\neq j\neq k}\frac{\rho \,j\,k\,\left(i\right)}{\rho \,j\,k}$$

In Eq. 1, ρjk is the number of shortest paths between nodes j and k, and ρjk (i) is the number of those pathways that pass *via* node i. By using this approach, unweighted shortest paths can be determined between nodes in a graph. In each node, the number of shortest paths passing through it determines its score. By using betweenness centrality, a network’s node can be assessed for its relative importance. The node with high betweenness centrality functions as an “interconnector” between several entities since it lies on the shortest path between them, connecting components that would otherwise be disconnected if the node was removed (Freeman, 1977).

In CiteSpace, burst detection is based on Kleinberg’s algorithm, which measures sudden increases in the amount of the data (Sohrabi et al., 2019). This indicator identifies an active research area by examining whether a publication or an author keyword has attracted extraordinary interest within its scientific community or whether an entity (such as an author, an institution, or a country) has published a significant amount of articles in a short period of time. A burst also detects a surge in citations for a particular reference or author keyword. It determines whether or not a given frequency function fluctuates significantly over a short period of time (Chen, 2016b). Burst detection therefore provides the opportunity to identify emerging terms and new directions in research (Dong et al., 2017).

A further feature of CiteSpace is that it allows knowledge mapping by visualization of bibliographic data, making it a popular and easy-to-use tool for the extraction of collaboration networks (between authors, institutions, and countries) and co-citation analysis (co-cited references).

An instance of co-citation occurs when two or more items (e.g., journals, articles, and authors) are cited by a third source (Small, 1973). Both Paper B and Paper C are referenced in Paper A, for example. The two papers B and C are considered to be “co-cited” by Paper A. Accordingly, the co-citation may indicate that the two papers contain similar content. Papers B and C are more likely to be related if they were co-cited by other papers (e.g., Papers D, E, and F). Document co-citation analyses were performed to determine how often two publications were co-cited (cited together) in subsequent publications to track time-evolution of influential publications and themes pursued. Using journal co-citation analysis, the most influential journals in the field were identified based on the number of times two journals were co-cited (cited jointly).

The identification of the main authors, institutions, and organizations involved in a research field, as well as their collaborations, is another fundamental topic. Through network analysis, it is possible to identify collaborations on a visual basis, where authors, affiliations, and nations are represented by nodes, while their collaboration is represented by lines or edges. The size of the node corresponds to the number of publications, whereas the thickness of the line or edge indicates the level of scientific collaboration (collaboration frequency) between the entities (i.e. authors, institutions, and nations). Also, isolated nodes can be distinguished by the lack of connections with other nodes, which indicates that they are not associated with any collaborations.

The width of different colored tree rings symbolizes the number of publications in various years, and the years shift from far to near as the ring rings move from inside to outside. The appearance of links and items is indicated *via* a color-coded spectrum (Xiang et al., 2017). A red tree ring indicates the burstiness of publication of the relevant entities (i.e., authors, institutions, or countries), indicating short periods of high scholarly activity.

The collaboration analysis can also be enhanced by determining a node’s level of association based on the betweenness centrality of their position within a network. Betweenness centrality measures the degree to which a node is relevant within a network, as it counts the number of regions on the map that are connected by that node. This indicates how important the node is to information flow in the network (Barthelemy, 2004; Lu et al., 2008). A node with a high betweenness centrality (>0.1) is illustrated by a purple outer ring, which is considered as a major entity that holds revolutionary theories or works and, in the meantime, controls significant resources in collaborative networks. Purple outer ring has a thickness that corresponds to its betweenness centrality.

Another open source, freely accessible software, VOSviewer, provides users with the ability to create and visualize bibliometric networks that are based on co-occurrence data. The co-occurrence analysis is used to evaluate the statistical relationship between two author keywords within a dataset; more frequently two author keywords are used together, the more likely they are to be logically related (Lawal et al., 2019).

Specifically, co-word analysis is predicated on the premise that the co-occurrence of author keywords, through characterizing the content of documents, captures semantic or conceptual groups of themes capable of depicting a field. Thus, co-word analysis is a method of mapping conceptual structure of frameworks *via* co-occurring author keywords in bibliographic records. VOSviewer is built on the normalized term co-occurrence matrix and a similarity measure that calculates the strength of association between terms, which means that terms that are conceptually similar are organized into clusters designated by the same cluster color (Van Eck and Waltman, 2013). These clusters are examined in order to identify research themes in the literature. The proximity of two keywords is indicative of their conceptual closeness. The content analysis of author keyword cluster landscape was conducted as described by Kokol et al. (2022). Further, author keyword networks for 2017–2019 and 2020–2022 were compared to find hot topics, as outlined by Kokol et al. (2018). The reasons for this separation of articles are threefold. First, since 2016, the Rome IV criteria have been in use and both the FD’s definitions and the prevailing research paradigm have undergone rapid evolution to keep up with the new standards. Therefore, for the purpose of tracing the state of the art in FD research, studies published after 2016 are preferred. Second, Figure 1 reflects a surge in publications since 2018, suggesting the emergence of novel research themes with the advent of Rome IV. Our understanding of FGIDs, specifically FD, has evolved significantly since the Rome IV definition. The extensive body of data that has been reported about the central, peripheral and genetic mechanisms involved in the pathophysiology of FD symptoms has enabled the development of a comprehensive disease model based on brain-gut-microbiome interactions. The understanding of the multi-factorial nature of FD has made quantum leaps in the Rome IV era. In response to these advances, FD has been viewed from a new perspective, which allows more pathophysiology-focused diagnoses and treatments for the disorder. As a result, the foci of research are constantly shifting, which differ greatly from the Rome III period, therefore requiring shorter timeframes within which to examine the literature trajectory. Lastly, the inclusion of all research published between 2016 and 2022 in a single dataset hinders the detection of subtle emerging topics in the final results. Therefore, we split the articles in the database into shorter time spans, such as 2 years.

## 3. Results

### 3.1. Publication output

A total of 2,957 research papers were published, including 2,532 articles (85.63%) and 425 reviews (14.37%). As shown in Figure 1, for better exploration of past research shifts, two time intervals were defined: 2006–2017 and 2018–2022. Between 2006 and 2017, the number of studies fluctuated in a wave-like pattern, ranging from 182 to 163 documents. During the last 4 years, the productivity grew exponentially, reaching 237 documents.

### 3.2. Analysis of countries, regions, and institutions

The literature on FD has been published by 369 institutions across 80 countries or regions. As shown in Table 1, the top 10 countries and institutions are listed. FD research was led by the USA and China, accounting for 19.17% and 14.20%, respectively, of the total number of publications in the field. Third place is held by Japan (371, 12.55%).

**TABLE 1:** The top 10 productive countries or regions and institutions of FD research.

Rank	Country	Centrality	Count (% of 2,957)	Rank	Institutions	Centrality	Count (% of 2,957)
1	USA	0.2	567 (19.17)	1	Mayo Clin (USA)	0.01	109 (3.69)
2	China	0	420 (14.20)	2	Univ Newcastle (England)	0.03	86 (2.91)
3	Japan	0.09	371 (12.55)	3	Katholieke Univ Leuven (Belgium)	0.06	50 (1.69)
4	Australia	0.2	191 (6.46)	4	Univ Leuven (Belgium)	0.04	49 (1.66)
5	England	0.13	174 (5.78)	5	McMaster Univ (Canada)	0.13	48 (1.62)
6	Italy	0.04	170 (5.75)	5	Chengdu Univ Tradit Chinese Med (China)	0.03	48 (1.62)
7	Belgium	0.02	162 (5.48)	6	Hyogo Coll Med (Japan)	0.07	40 (1.35)
8	South Korea	0.01	161 (5.44)	7	Nippon Med Sch (Japan)	0.06	38 (1.29)
9	Germany	0.2	142 (4.80)	7	Univ Adelaide (Australia)	0.07	38 (1.29)
10	Canada	0.25	118 (3.99)	8	China Acad Chinese Med Sci (China)	0.09	36 (1.22)
				8	Univ Malaya (Malaysia)	0.11	36 (1.22)
				9	Beijing Univ Chinese Med (China)	0.02	34 (1.15)
				10	Macquarie Univ (Australia)	0.04	31 (1.04)

Over the past decade, Western institutions have dominated the top 10 productive list. Mayo Clin produced the most papers (109, 3.68%), followed by Univ Newcastle (86, 2.91%) and Katholieke Univ Leuven (50, 1.69%).

The top countries with the highest betweenness centrality were Canada (0.25), the USA (0.2), and Australia (0.2). In terms of betweenness centrality, McMaster Univ (0.13) ranked first. Univ Malaya (0.11) and China Acad Chinese Med Sci (0.09) came in second and third, respectively.

**FIGURE 2:**
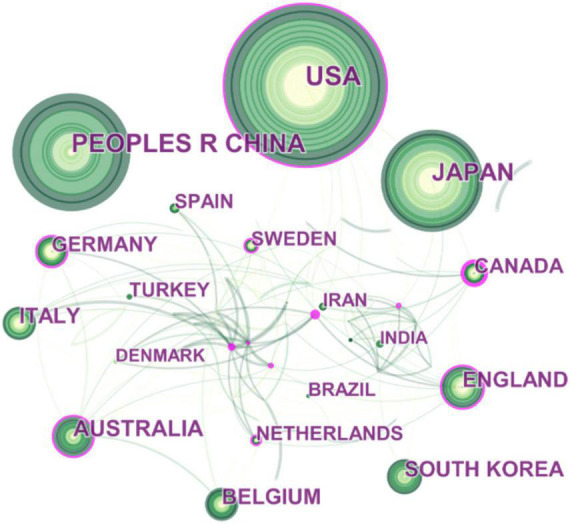
Collaboration network of countries or regions engaged in FD research. The network is made up of nodes, each representing a country or region, and their size represents the number of publications produced by that country or region. A thicker curved line connecting nodes indicates a more frequent co-occurrence, since co-occurrence represents a collaborative relationship. There is no collaboration when a node lacks any connections. A node with a high betweenness centrality (>0.1) (that is, those that are linked to more than 10% of the other nodes) possess significant power over other nodes due to the increased flow of information *via* it. Furthermore, the presence of a purple rim denotes a high degree of betweenness centrality. The presence of a red tree ring indicates a high level of scholarly activity, indicated by a citation burst. The burst associated with a particular node is proportional to the thickness of its red tree ring.

In Figure 2, the collaboration network among different countries contributing to the FD literature is illustrated. In the collaboration cluster community, Canada, the USA, Australia, England, and Germany were key players due to the global nature of their cooperation. In addition, Greece, Norway, Switzerland, Sweden, Singapore, France, and the Netherlands were actively involved in collaborative research, despite not having a high record of publications.

For example, Canada, which was engaged in the most extensive global collaborations, worked closely with the USA, Belgium, England, France, Denmark, the Netherlands, Germany, Portugal, Italy, Sweden, Greece, Czechia, Argentina, Brazil, Peru, Saudi Arabia, Israel, New Zealand, Australia, Thailand, Japan, Iran, and Rwanda.

**FIGURE 3:**
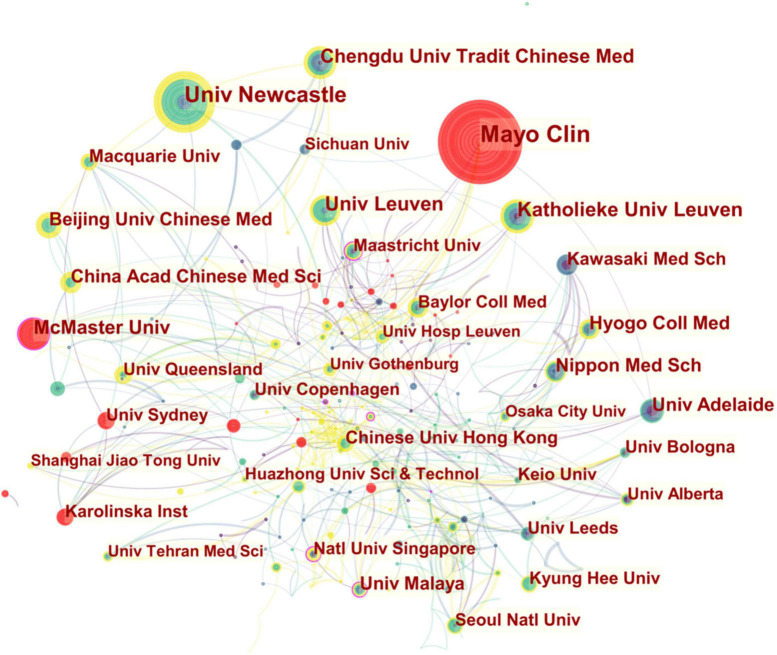
Collaboration network of institutions engaged in FD research. The network is made up of nodes, each representing an institution, and their size represents the number of publications produced by that institution. A thicker curved line connecting nodes indicates a more frequent co-occurrence, since co-occurrence represents a collaborative relationship. There is no collaboration when a node lacks any connections. A node with a high betweenness centrality (>0.1) (that is, those that are linked to more than 10% of the other nodes) possess significant power over other nodes due to the increased flow of information *via* it. Furthermore, the presence of a purple rim denotes a high degree of betweenness centrality. The presence of a red tree ring indicates a high level of scholarly activity, indicated by a citation burst. The burst associated with a particular node is proportional to the thickness of its red tree ring.

As shown in Figure 3, McMaster Univ and Univ Malaya dominated the institutional collaboration network. The main institutions that collaborated with McMaster Univ were Univ Calgary (Canada), Univ Toronto (Canada), Johns Hopkins Univ (USA), St Louis Univ (USA), Univ Wisconsin (USA), Surrey GI Res (England), Leeds Gen Infirm (England), Leeds Teaching Hosp NHS Trust (England), St James Univ Hosp (England), Univ Leeds (England), Dartmouth Hitchcock Med Ctr (Lebanon), and Chulalongkorn Univ Hosp (Thailand).

Univ Malaya had notably strong collaborations with Univ Padua (Italy), Univ Milan (Italy), St James Univ Hosp (England), Natl Def Med Coll (Japan), Yonsei Univ (South Korea), Wonkwang Univ (South Korea), Natl Univ Singapore (Singapore), Natl Univ Singapore Hosp (Singapore), Chulalongkorn Univ Hosp (Thailand), and Sheikh Russel Natl Gastroliver Inst & Hosp (Bangladesh).

### 3.3. Authors

There were 422 authors contributing to the FD studies. Table 2 illustrates that authors affiliated with European institutions published the majority of FD articles (199). The most articles (128, 4.33%) were contributed by Jan Tack, followed by Nicholas J. Talley (122, 4.13%), and Hiroto Miwa (32, 1.08%). Nicholas J. Talley (0.41), Jan Tack (0.27), Gerald Holtmann (0.15), and Michael Camilleri (0.13) were the top authors in terms of betweenness centrality.

**TABLE 2:** The top 10 productive authors of FD research.

Rank	Authors	Count (% of 2,957)	Centrality
1	Jan Tack (Belgium)	128 (4.33)	0.27
2	Nicholas J. Talley (Australia)	122 (4.13)	0.41
3	Hiroto Miwa (Japan)	32 (1.08)	0.08
4	Marjorie M. Walker (Australia)	29 (0.98)	0.02
5	Paul Moayyedi (Canada)	28 (0.95)	0.11
5	Alexander C. Ford (England)	28 (0.95)	0.07
6	Gerald Holtmann (Australia)	26 (0.95)	0.15
7	Michael Camilleri (USA)	25 (0.95)	0.13
7	Fanrong Liang (China)	25 (0.95)	0
7	Sanjiv Mahadeva (Malaysia)	25 (0.95)	0.03
7	Fang Zeng (China)	25 (0.95)	0
8	Seiji Futagami (Japan)	23 (0.78)	0.09
8	Lukas Van Oudenhove (Belgium)	23 (0.78)	0.06
9	Tim Vanuytsel (Belgium)	20 (0.68)	0.02
9	Jae-Woo Park (South Korea)	20 (0.68)	0.05
9	Ken Haruma (Japan)	20 (0.68)	0.13
9	Brian E. Lacy (USA)	20 (0.68)	0
10	Tadayuki Oshima (Japan)	18 (0.61)	0

**FIGURE 4:**
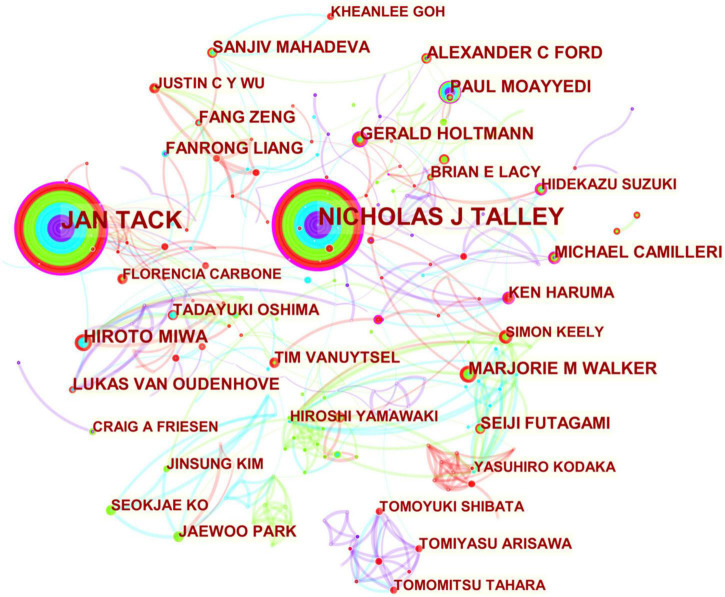
Collaboration network of authors engaged in FD research. The network is made up of nodes, each representing an author, and their size represents the number of publications produced by that author. A thicker curved line connecting nodes indicates a more frequent co-occurrence, since co-occurrence represents a collaborative relationship. There is no collaboration when a node lacks any connections. A node with a high betweenness centrality (>0.1) (that is, those that are linked to more than 10% of the other nodes) possess significant power over other nodes due to the increased flow of information *via* it. Furthermore, the presence of a purple rim denotes a high degree of betweenness centrality. The presence of a red tree ring indicates a high level of scholarly activity, indicated by a citation burst. The burst associated with a particular node is proportional to the thickness of its red tree ring.

The authors’ collaboration network, illustrated in Figure 4, showed that among the topmost productive authors, Nicholas J. Talley, Jan Tack, Gerald Holtmann, Michael Camilleri, Ken Haruma, and Paul Moayyedi were leading authors within their collaborative clusters.

Nicholas J. Talley had close ties with Jan Tack, Hidekazu Suzuki (Japan), Magnus Simrén (Sweden), Alexander C. Ford, Guido Gerken (Germany), John K. DiBaise (USA), Cathy D. Schleck (USA), Brian E. Lacy, Paul Moayyedi, Alan R. Zinsmeister (USA), Linda M. Herrick (USA), Rok Seon Choung (USA), Kerith Duncanson (Australia), Grace Burns (Australia), Mike Jones (Australia), Jennifer Pryor (Australia), Michael P. Jones (Australia), Simon Keely (Australia), Gerald Holtmann, Marjorie M. Walker, Henry P. Parkman (USA), Awat Feizi, and Peyman Adibi (Iran).

Jan Tack had close collaborations with Alexander C. Ford, Dominique Vanderghinste (Belgium), Magnus Simrén, Hans Törnblom (Sweden), Brecht Geeraerts (Belgium), Patrick Dupont (Belgium), Tim Vanuytsel, Rita Vos (Belgium), Ricard Farré (Belgium), Lukas Van Oudenhove, Benjamin Fischler (Belgium), Hanne Vanheel (Belgium), Joris Vandenberghe (Belgium), M. Florencia Carbone (Belgium), Dorien Beeckmans (Belgium), Nathalie Weltens (Belgium), Hidekazu Suzuki, Tatsuhiro Masaoka (Japan), Michael Camilleri, Henry P. Parkman, Nimish Vakil (USA), Paul Moayyedi, Nicholas J. Talley, Christine Feinle-Bisset (Australia), Michael P. Jones, and Fernando Azpiroz (Spain).

Gerald Holtmann produced internationally collaborative publications with Michael P. Jones, Peter Malfertheiner (Germany), Ayesha Shah (Australia), Kok-Ann Gwee (Singapore), Nicholas J. Talley, and Jin-Song Liu (China).

Paul Moayyedi collaborated closely with Nicholas J. Talley, Hidekazu Suzuki, Alexander C. Ford, Colin W. Howden (USA), Alan R. Zinsmeister, Cathy D. Schleck, Brian E. Lacy, Premysl Bercik (Canada), Enzo Ubaldi (Italy), Luigi Gatta (Italy), Cesare Tosetti (Italy), Giulia Fiorini (Italy), and Dino Vaira (Italy).

Ken Haruma engaged in broad collaborations with Hidekazu Suzuki, Kazuaki Chayama (Japan), Shinji Tanaka (Japan), Yoshikazu Kinoshita (Japan), Hiroto Miwa, Tomoari Kamada (Japan), Noriaki Manabe (Japan), Michael Camilleri, and Alexander C. Ford.

Michael Camilleri worked closely with Jessica Atieh (USA), Nicholas J. Talley, Jan Tack, Noriaki Manabe, Alan R. Zinsmeister, Cathy D. Schleck, Yuri A. Saito (USA), Linda M. Herrick, and Myung-Gyu Choi (South Korea).

### 3.4. Productive and co-cited journals

There were 742 journals in total that published the retrieved records. The top 10 productive journals in FD research are listed in Table 3. *Neurogastroenterology and motility: the official journal of the European Gastrointestinal Motility Society* published the most literature (204, 6.90%).

**TABLE 3:** Top 10 prolific journal and top 10 co-cited journals in FD research.

Rank	Journal	Count (% of 2,957)	IF	JCR	Rank	Co-cited journal	Count (% of 31,858)	IF	JCR
1	*Neurogastroenterology and motility: the official journal of the European Gastrointestinal Motility Society* (England)	204 (6.90)	3.960	Q2	1	*Gastroenterology* (USA)	2,139 (6.71)	33.883	Q1
2	*Journal of gastroenterology and hepatology* (Australia)	96 (3.25)	4.369	Q2	2	*Gut* (USA)	1,952 (6.13)	31.793	Q1
2	*World journal of gastroenterology* (USA)	91 (3.01)	5.374	Q2	3	*The American journal of gastroenterology* (USA)	1,908 (5.99)	12.045	Q1
4	*Journal of neurogastroenterology and motility* (South Korea)	80 (2.71)	4.725	Q2	4	*Alimentary pharmacology & therapeutics* (England)	1,687 (5.30)	9.524	Q1
4	*Digestive diseases and sciences* (USA)	80 (2.71)	3.487	Q3	5	*Digestive diseases and sciences* (USA)	1,329 (4.17)	3.487	Q3
5	*Alimentary pharmacology & therapeutics* (England)	74 (2.50)	9.524	Q1	6	*Neurogastroenterology and motility: the official journal of the European Gastrointestinal Motility Society* (England)	1,222 (3.84)	3.960	Q2
6	*The American journal of gastroenterology* (USA)	55 (1.85)	12.045	Q1	7	*Scandinavian journal of gastroenterology* (England)	1,086 (3.41)	3.027	Q4
7	*Scandinavian journal of gastroenterology* (England)	45 (1.52)	3.027	Q4	8	*World journal of gastroenterology* (USA)	1,044 (3.28)	5.374	Q2
8	*BMC gastroenterology* (England)	44 (1.49)	2.847	Q4	9	*Journal of gastroenterology and hepatology* (Australia)	1,010 (3.17)	4.369	Q2
8	*European journal of gastroenterology & hepatology* (England)	44 (1.49)	2.586	Q4	10	*Clinical gastroenterology and hepatology: the official clinical practice journal of the American Gastroenterological Association* (USA)	958 (3.01)	13.576	Q1
9	*Journal of gastroenterology* (Japan)	42 (1.42)	6.772	Q2					
10	*Clinical gastroenterology and hepatology: the official clinical practice journal of the American Gastroenterological Association* (USA)	41 (1.39)	13.576	Q1					
10	*Helicobacter* (England)	41 (1.39)	5.182	Q2					

We recorded the 2022 Journal Citation Reports (JCR) Journal Impact Factor (JIF) of a journal from the Web of Science. A journal’s JIF is determined by dividing the number of citations to its articles in the previous 2 years by the number of citable articles it published during those 2 years. To measure the quality of a journal, all journals in a particular subject are sorted according to their IF values from the previous year, then divided into four quartiles: Q1, Q2, Q3, and Q4. Journal co-citation occurs when a journal is co-cited (cited together) with another journal in a research paper, often indicating similar topics between the two journals. The journal co-citation analysis is used to determine the most influential journals.

A journal co-citation analysis was also performed in order to identify influential journals in FD research. FD-related research papers were co-cited in 174 scholarly journals. As shown in Table 3, *Gastroenterology* had the most co-citations (2,139, 6.71%).

### 3.5. Co-cited references and references with citation bursts

Two articles are co-cited when they are both cited in a third article. By combining highly cited papers and the evolution of research trends, co-cited references (documents) illustrate the intellectual structure of the research. The use of co-citation references also allows us to estimate the contribution of countries, institutions, authors, and journals to the genesis of the intellectual foundation. It follows the same method as the previous co-citation analysis, but instead of journals, it analyzes documents. A total of 1,155 references were co-cited in the 2,957 FD publications. Table 4 shows the top 10 co-cited references.

**TABLE 4:** Top 10 co-cited references in FD research.

Rank	References	Journal	Co-citation	Publishing year
1	Epidemiology, clinical characteristics, and associations for symptom-based Rome IV functional dyspepsia in adults in the USA, Canada, and the UK: a cross-sectional population-based study	*The lancet gastroenterology & hepatology*	75	2018
2	Effect of amitriptyline and escitalopram on functional dyspepsia: a multicenter, randomized controlled study	*Gastroenterology*	64	2015
3	Impaired duodenal mucosal integrity and low-grade inflammation in functional dyspepsia	*Gut*	62	2014
4	A placebo-controlled trial of acotiamide for meal-related symptoms of functional dyspepsia	*Gut*	52	2012
5	Worldwide prevalence and burden of functional gastrointestinal disorders, results of Rome foundation global study	*Gastroenterology*	48	2021
6	Randomised clinical trial: rifaximin versus placebo for the treatment of functional dyspepsia	*Alimentary pharmacology & therapeutics*	47	2017
7	Functional dyspepsia: the economic impact to patients	*Alimentary pharmacology & therapeutics*	46	2013
7	Pathophysiological abnormalities in functional dyspepsia subgroups according to the Rome III criteria	*The American journal of gastroenterology*	46	2017
8	Anxiety is associated with uninvestigated and functional dyspepsia (Rome III criteria) in a Swedish population-based study	*Gastroenterology*	44	2009
9	Efficacy of mirtazapine in patients with functional dyspepsia and weight loss	*Clinical gastroenterology and hepatology: the official clinical practice journal of the American Gastroenterological Association*	38	2016
10	Duodenal eosinophilia and early satiety in functional dyspepsia: confirmation of a positive association in an Australian cohort	*Journal of gastroenterology and hepatology*	37	2014
10	Functional dyspepsia impacts absenteeism and direct and indirect costs	*Clinical gastroenterology and hepatology: the official clinical practice journal of the American Gastroenterological Association*	37	2010
10	Alteration in the gastric microbiota and its restoration by probiotics in patients with functional dyspepsia	*BMJ open gastroenterology*	37	2017

**FIGURE 5:**
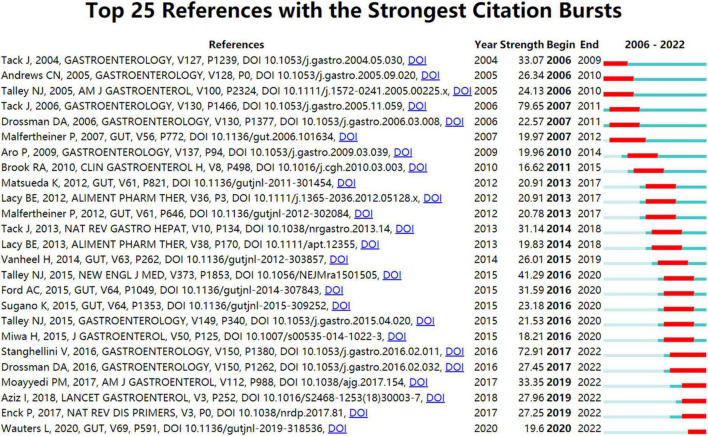
Top 25 references with strong citation bursts in FD research. Burst strength is a measure of the rate of change in citations. A citation burst with greater strength indicates a greater number of citations during a given period. During the period of 2006–2022, a thin blue line runs, while the bold red line marks a reference burst, which is characterized by an increase in citations over a short period of time.

Citation bursts can be interpreted as evidence of a spike in citations following the publication of a specific article. Citation bursts, therefore, indicate active areas of research or themes that are at the forefront of research. There are strong citation bursts for 25 references illustrated in Figure 5. When identifying research hotspots, it is crucial to consider references (Drossman, 2016; Stanghellini et al., 2016; Enck et al., 2017; Moayyedi et al., 2017; Aziz et al., 2018; Wauters et al., 2020b) whose citation bursts ended in 2022, as these references continue to generate rapidly escalating citation counts.

### 3.6. Keyword analysis

**FIGURE 6:**
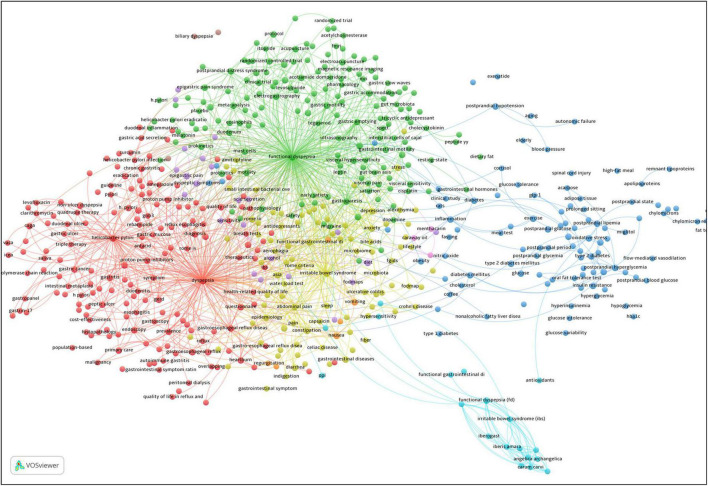
Co-occurrence network of author keywords with a minimum of 5 occurrences in FD research. A node size indicates the number of occurrences of an author keyword. An edge between nodes indicates that author keywords are commonly occurring. The greater the thickness of an edge, the more frequently an author keyword co-occurs with the other keyword. The color of a node indicates its category.

The co-occurrence analysis, namely content analysis, is one of several types of synthetic knowledge synthesis. It is derived by co-occurrence of keywords and concepts within texts and sources, which provides an accurate way to identify the main concepts within a particular field or scientific area. It is therefore possible to discover, plot, and manage patterns and conceptual events as well as scientific structure, conceptual network, hierarchical relationships between concepts and conceptual categories of the field being studied. The conceptual network is constructed by counting the number of thematic words in the text and their relationship to other topics. The more frequently two terms are used in a document and repeated, the more semantically related they are. Using the co-occurrence of two terms or two keywords allows us to discover the connections between two topics in a field of research, and in this way we can gain insight into the developmental trends of that field. In the literature retrieved by VOSviewer, 4,725 author keywords were identified. In preparation for creating bibliometric maps, thesauri were developed, which disambiguated the various forms in which author keywords appear, combined synonyms, corrected spelling mistakes, and removed unnecessary terms. Additionally, author keywords that occurred more than five times were included in the analysis, and small clusters with fewer than 30 author keywords were automatically merged. A total of 360 qualified author keywords were identified and categorized into seven clusters in the network. Synthetic knowledge synthesis performed on author keywords cluster landscape (Figure 6) resulted in six themes and 27 categories presented in Table 5. The frequent codes of each cluster were shown in Supplementary Figures 1–6.

**TABLE 5:** Results of the synthetic knowledge synthesis in FD research.

Theme	Color	Representative author keywords	Prevailing sub-categories
*H. pylori* infection	Red	Gastric cancer, gastropanel, gastrin-17, cytotoxin associated gene A (CagA), vacuolating cytotoxin, duodenal ulcer promoting gene (dupA), malignancy, gastric ulcer, duodenal ulcer, PPI, antacids, rebamipide, reflux esophagitis, quadruple therapy, triple therapy	The association between *H. pylori* and the development of noncardia gastric cancer and peptic ulcer disease; the molecular basis by which *H. pylori* induces gastric cancer; diagnostic tests for *H. pylori* infection; antibiotic eradication of *H. pylori* infection; screening and treatment for *H. pylori* infection; the link between gastroesophageal reflux disease (GERD) and *H. pylori*; the involvement of *H. pylori* in FD
Pathophysiological mechanisms of FD	Green	Duodenal inflammation, mast cells, eosinophils, GNB3, interleukin (IL)-17F, IL-10, melatonin, gastric motility, impaired gastric accommodation, gastric slow waves, leptin, peptide YY, cholecystokinin, glucagon-like peptide 1, motilin, gastric emptying, tegaserod, itopride, prokinetics, acotiamide, gut microbiota, small intestinal bacterial overgrowth (SIBO), the interstitial cells of Cajal (ICCs), visceral hypersensitivity, functional magnetic resonance imaging (fMRI), bile acids, brain-gut axis, enteric nervous system, vagal nerve, food allergy, dopamine, acupuncture, electroacupuncture	Neuronal mechanisms of visceral hypersensitivity in FD; abnormal visceral pain signaling in FD; subtle infiltration and activation of eosinophils and mast cells; dysregulation of the gut-brain axis in FD; neuropeptides in the pathogenesis and treatment of FD; dysregulation of enteroendocrine cells signaling in FD; pathophysiologic involvement of ICCs in FD; bile acid alterations as an important determinant in FD etiopathogenesis; food allergy as stimulants in FD symptoms; brain network alterations in FD; acupuncture treatment of FD
Extraintestinal co-morbidities and overlap syndromes associated with FD	Yellow	Migraine, chronic pelvic pain, fibromyalgia, chronic fatigue, endometriosis depression, sleep disorders, insomnia, anxiety, antidepressants, posttraumatic stress disorder, alexithymia, post-cholecystectomy syndrome, aerophagia, Crohn’s disease, celiac disease, lactose intolerance, GERD, IBS, functional constipation	The extraintestinal co-morbidities of FD, many of which are chronic painful disorders; psychological co-morbidities associated with FD; FGID overlap syndrome; FD patients with overlapping GERD
Herbal medicine in FD	Light blue; pink	Iberogast (STW5), *Iberis amara*, *Mentha piperita*, *Glycyrrhiza glabra*, *Angelica archangelica*, *Carum carvi*, *Chelidonium majus*, carraway oil, peppermint oil	Phytomedical treatment for FD
Diabetic gastroparesis	Dark blue	Glucose tolerance, type 2 diabetes mellitus, type 1 diabetes mellitus, post-prandial hyperglycemia, hyperinsulinemia, hypertriglyceridaemia, and insulin resistance, nonalcoholic fatty liver disease, high-fat meal, oxidative stress, nitric-oxide, adipose tissue, exercise, prolonged sitting	The overlap of symptoms in FD and gastroparesis; pathophysiology of FD and gastroparesis and overlap
Dietary factors in FD	Purple	Body mass index (BMI), alcohol, FODMAPs (fermentable oligo-, di-, and monosaccharides and polyols), gluten wheat	Mechanisms underlying food- and eating-induced symptoms of FD; diet-focused approach to treating FD

**FIGURE 7:**
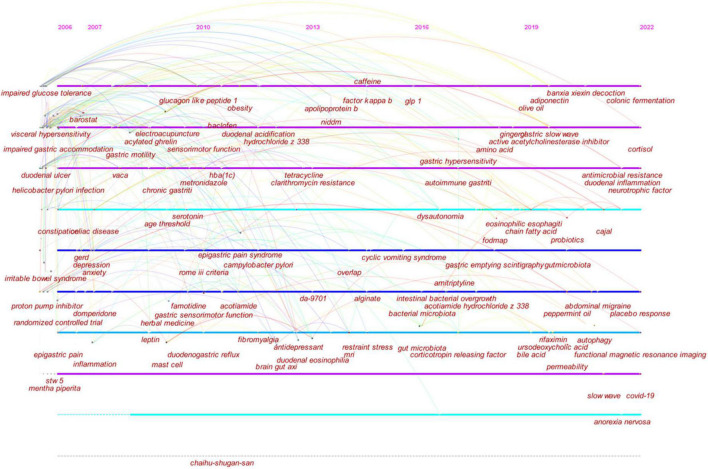
Timeline visualization of co-occurring author keywords network. In the map, each author keyword is displayed along with the year in which it first appeared. Each node represents an author keyword. Co-occurrences of author keywords are represented by the links. The node’s size is proportional to the frequency with which an author keyword occurs.

In Figure 7, the keyword co-occurrences were visualized in chronologic order, where the timeline view enables clear identification of the different research trends and their evolution.

In regard to the period from 2006 to 2010, the primary foci of the research were: (1) barostat testing, nutrient drink test, water load test, stool antigen test, ^13^C-octanoic acid breath test, gastric emptying scintigraphy, intraluminal electrical impedance, and electrogastrography; (2) *H. pylori* infection; (3) BMI and smoking; (4) GERD, hiatal hernia, atrophic gastritis, intestinal metaplasia, gastric adenocarcinoma, and duodenal ulcer; (5) duodenogastric reflux; (6) dysrhythmia; (7) gastric fundus tone, antroduodenal motility, impaired gastric accommodation, slow gastric emptying, and gastric acid secretion; (8) visceral hypersensitivity and visceral perception; (9) type 2 diabetes mellitus, insulin resistance, and impaired glucose tolerance; (10) metabolic syndrome; (11) chest pain, nausea, constipation, epigastric pain, heartburn, and gastric distension; (12) psychological factor, abuse history, and stress; (13) triple therapy, metronidazole, PPIs, baclofen, alosetron, atropine, prokinetic agent, serotonin reuptake inhibitor, psychotherapy, and nonsteroidal anti-inflammatory drug; (14) *Mentha* × *piperita* L., *Glycyrrhiza glabra*, *Iberis amara*, *Matricaria recutita*, *Carum carvi*, *Chamomilla recutita*, *Chelidonium majus*, caraway oil, *Silybum marianum*, *Angelica archangelica*, and STW5; (15) electrical stimulation and acupuncture; (16) diabetic gastroparesis and autonomic neuropathy; (17) stress, anxiety, and depression; (18) endocrine cell; (19) melatonin, leptin, 5-hydroxytryptamine (serotonin), and ghrelin; (20) cholecystokinin A receptor; (21) gut microbiota; (22) dietary fiber, gluten, high fat meal, lipid metabolism, and carbohydrate metabolism; (23) duodenal eosinophilia and oxidative stress; (24) antioxidant, arginine, nitric oxide, and glutathione; and (25) CagA and vacuolating cytotoxin.

From 2011 to 2016, the field focused on (1) obesity, hypertriglyceridemia, and hyperglycemia; (2) IBS and Crohn’s disease; (3) non-insulin-dependent diabetes mellitus; (4) chronic pancreatitis; (5) magnetic resonance imaging and single photon emission computed tomography; (6) gastric myoelectrical activity, gastric sensorimotor dysfunction, and duodenal acidification; (7) capsaicin; (8) satiety, belching, and bloating; (9) cyclic vomiting syndrome, functional abdominal pain, fibromyalgia, allergy, restraint stress, sleep disorder, and insomnia; (10) caffeine and coffee; (11) brain activity and anterior cingulate cortex; (12) aerobic exercise; (13) alginate, tetracycline, antidepressants, and DA-9701; (14) acotiamide; (15) exenatide; (16) ferulic acid, citrate, and chlorogenic acid; (17) glucagon-like peptide 1; (18) alanine aminopeptidase and adipose tissue; (19) dupA; (20) GNB3 825T allele; (21) gut-brain axis and brain network; (22) mast cells; and (23) nuclear factor-κB.

From 2017 to 2022, researchers focused their research on (1) electrogastrogram, fMRI, and Gastroparesis Cardinal Symptom Index; (2) alcohol; (3) default mode network; (4) overlap syndromes, anorexia nervosa, abdominal migraine, eosinophilic oesophagitis, and lactose intolerance; (5) herbal medicine, 6-gingerol, and olive oil; (6) rifaximin, bismuth, amitriptyline, escitalopram, and digestive enzyme; (7) cognitive behavioral therapy; (8) placebo response; (9) low FODMAPs diet, isomaltase, and fructose; (10) cortisol and corticotropin-releasing factor; (11) brain-gut peptide, neurotrophic factor, adiponectin, and interstitial cells of Cajal; (12) bile acid, ursodeoxycholic acid, and SIBO; (13) coronavirus disease 2019; (14) duodenal inflammation; and (15) dynamin-related protein 1.

**FIGURE 8:**
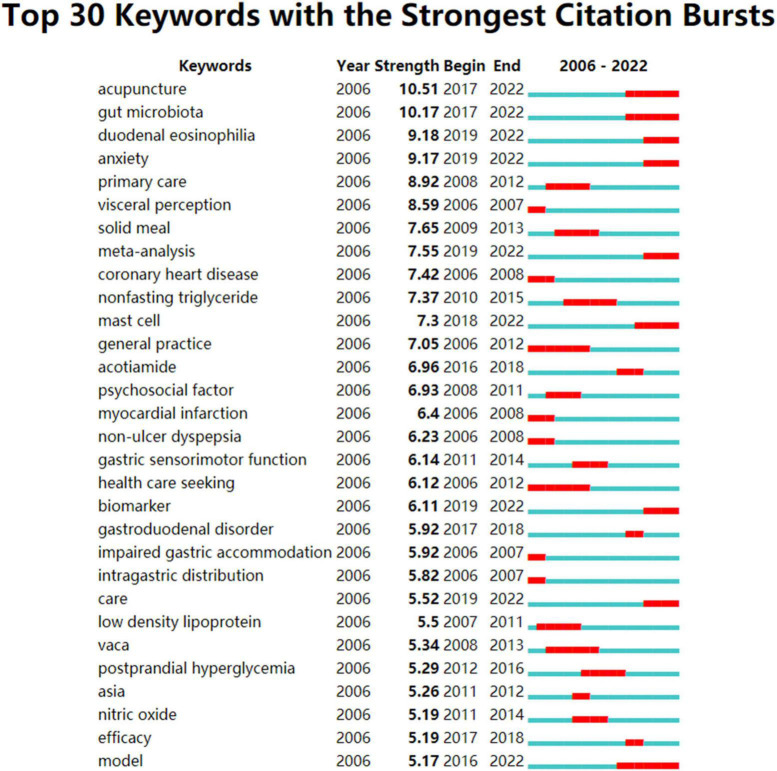
Top 30 author keywords with strong citation bursts in FD research. Burst strength is a measure of the rate of change in citations. A citation burst with greater strength indicates a greater number of citations during a given period. During the period of 2006–2022, a thin blue line runs, while the bold red line marks an author keyword burst, which is characterized by an increase in citations over a short period of time.

In the burstiness analysis in Figure 8, the top 30 author keywords were extracted; the ongoing bursts for the investigated period were “acupuncture,” “duodenal eosinophilia,” “gut microbiota,” and “anxiety.”

A list of author keywords with a high frequency in FD research is presented in Table 6. The most frequent keywords were “*H. pylori* infection” (705), “IBS” (602), “GERD” (367), and “quality of life” (311).

**TABLE 6:** Top 20 author keywords with the highest frequency in FD research.

Rank	Keywords	Count	Rank	Keywords	Count
1	*H. pylori* infection	705	11	Anxiety	121
2	IBS	602	12	Impaired glucose tolerance	116
3	GERD	367	13	Gastroparesis	107
4	Quality of life	311	14	Depression	101
5	Impaired gastric accommodation	252	15	Type 2 diabetes mellitus	99
6	Duodenal ulcer	239	16	Atrophic gastritis	97
7	PPIs	207	17	Gastric emptying	94
8	Epigastric pain	186	18	Prokinetic agents	90
9	Visceral hypersensitivity	147	19	Oxidative stress	77
10	Acupuncture	135	20	Inflammation	76

Clustered scientific landscapes (Supplementary Figures 7, 8) were generated using VOSviewer for two consecutive periods (2017–2019 and 2020–2022), as described by Kokol et al. (2018). Both landscapes were subjected to thematic analysis, and the landscapes were analyzed and compared in order to identify new research topics. Notably, terms related to *H. pylori*-associated dyspepsia and disordered motility appeared in 2017–2019 but not in 2020–2022, indicating that the research fervor on these topics is fading. Further, it was revealed from the hot topic analysis that FD research in the last 5 years has centered on:

(1) subtle immune activation and inflammatory responses in FD characterized by infiltration and activation of eosinophils and mast cells;
(2) acupuncture therapy in FD;
(3) microbial dysbiosis in FD;
(4) FD’s frequent co-morbidity with other FGIDs, as well as chronic pain and psychiatric disorders;
(5) the mechanisms responsible for the development of visceral hypersensitivity in FD;
(6) the mechanisms of food-induced symptoms and dietetic management in FD.

In the present study, the hot topics result obtained by VOSviewer was consistent with the author keyword burstiness analysis from CiteSpace, suggesting reliability in the findings.

## 4. Discussion

### 4.1. General information

This is the first exhaustive analysis of FD research that employs a novel knowledge synthesis method and cutting-edge bibliometric techniques to map out areas of FD research, analyze scientific context within those areas, and assess how it has evolved over time.

As indicated by the number of publications in this field shown in Figure 1, this field remained stagnant until 2017, when a sudden explosion of scientific productivity became apparent. Therefore, the last 4 years have witnessed intriguing findings that have attracted considerable attention.

Table 1 shows that the USA, China, and Japan dominated scientific production in this field. A major contributing factor to the USA and China’s active performances is likely the second phase of Integrative Human Microbiome Project launched by the National Institutes of Health in 2013 and the Microbiome Program of the Chinese Academy of Sciences in 2017. These programs provided financial support for related research, which further attracted psychiatrists, microbiologists, neurologists and gastroenterologists to the area and sparked their interest in it.

The centrality indicator can be used to quantify the significance of each entity (i.e., an author, an institution, or a country) within the co-authorship network; due to its short path to other nodes, a node with a high degree of betweenness centrality can be viewed as a bridge between different entities (represented by a purple halo) (Abbasi et al., 2012). In other words, a higher centrality index indicates stronger engagement with other entities as well as greater influence in the academic community (Freeman, 1977; Fotopoulou et al., 2022).

In this regard, as shown in Figure 2, collaboration among countries was headed by Canada, the USA, Australia, England, and Germany; also, during the period of cooperation, they held a leading position in this field by authoring extremely influential publications. This is not a surprising result because in another bibliometric analysis which sought to provide a comprehensive insight into the scientific profile of IBS research, it was found that the USA, Canada, England, Sweden, and others were identified as central co-authoring countries due to their worldwide collaboration and production of impactful research (Zhong and Lin, 2022). This indicates that European and North American countries have invested significant resources and efforts in conducting high-quality studies in FGIDs field.

In Table 1 and Figure 3, Western institutions topped the most productive list; Japanese, Chinese, and Malaysian institutions also demonstrated robust publishing activities.

An exceptional bibliometric profile and a high record of publications made McMaster Univ the most impactful among the top 10 most productive affiliations. Furthermore, Univ Malaya had a remarkable number of publications and a high degree of betweenness centrality, indicating its exceptional academic impact on FD research. Although Univ Padua (10 publications; betweenness centrality 0.14), Natl Univ Singapore (26 publications; betweenness centrality 0.13), Queen Mary Univ London from England (15 publications; betweenness centrality 0.12), Maastricht Univ from the Netherlands (21 publications; betweenness centrality 0.11), and Royal Adelaide Hosp from Australia (17 publications; betweenness centrality 0.11) did not contribute to the top 10 list due to their low literature output, they demonstrated a high degree of collaboration and their scientific publications were viewed as potentially revolutionary contributions to the field. A significant increase in the number of Asian institutions participating in FD research has been observed; however, similar to the country co-authorship landscape, institutions in North America, Europe, and Australia generally tended to collaborate more actively and produce highly influential scientific outcomes.

In Table 2 and Figure 4, Asian scholars exhibited the highest levels of publication activity despite a low degree of international collaboration. As regards betweenness centrality, Nicholas J. Talley, Jan Tack, Gerald Holtmann, Michael Camilleri, Ken Haruma, and Paul Moayyedi were ranked at the top of the list, reiterating the excellence of North America, Europe, and Australia in FD research. The results of our study lend empirical support to recent papers (Zyoud et al., 2019, 2021; Chen et al., 2022a; Zhang T. et al., 2022b) that argues greater diversity of collaboration is necessary to advance FGIDs field in Asia. Research on FD will therefore have much to offer since Asian research into FD is still a fledgling.

As indicated in Table 3, the active journals that published FD studies provide substantial coverage to gastroenterology-related topics. Furthermore, they are mostly based in England. In order to identify the journals that have influenced the field, which researchers dedicate considerable attention to, the co-citation technique was used. The most highly co-cited journals are, however, based in the USA, although it is important to note that *Alimentary pharmacology & therapeutics*, *Neurogastroenterology and motility: the official journal of the European Gastrointestinal Motility Society*, and *Scandinavian journal of gastroenterology* also received high co-citations.

High-quality, well-designed studies are required to bolster the evidence base for FD research, as the most prolific journals are found in Q2 or Q4. In addition, it is proposed that the professional capacity building of journals published in Asian nations be strengthened in order to produce high-quality scientific results and share knowledge gained in the field of FD in Asia.

*Neurogastroenterology and motility: the official journal of the European Gastrointestinal Motility Society*, *World journal of gastroenterology*, *Journal of gastroenterology and hepatology*, *Digestive diseases and sciences*, *Alimentary pharmacology & therapeutics*, *The American journal of gastroenterology*, and *Scandinavian journal of gastroenterology* were considered core journals in the field based on their high publications and co-citations.

### 4.2. Knowledge base

The document co-citation analysis was performed to identify “*intellectual turning point papers*” in the field of FD, i.e., papers that made significant contributions to domain knowledge. Based on Table 4, the most influential articles describe the epidemiology and economic burden of FD, as well as its pathophysiology (e.g., duodenal inflammation; dysbiosis; and psychological factors) and associated therapeutic approaches, as well as overlap syndromes in FD (Aro et al., 2009; Brook et al., 2010; Matsueda et al., 2012; Lacy et al., 2013; Vanheel et al., 2014, 2017; Walker et al., 2014; Talley et al., 2015; Tack et al., 2016; Igarashi et al., 2017; Tan et al., 2017; Aziz et al., 2018; Sperber et al., 2021).

Based on an analysis of the most frequently used author keywords in FD papers, as displayed in Table 5, the extensively studied topics include: (1) IBS, FD and GERD overlap; (2) gastroparesis and FD; (3) the pathophysiology of FD (e.g., disturbed gastric accommodation, impaired gastric emptying, visceral hypersensitivity, psychosocial factors, and gastroduodenal mucosa inflammation); and (4) treatments options such as PPIs, prokinetics, and acupuncture.

### 4.3. Emerging trends

In addition to highlighting the fast-moving or newly emerging areas in FD research based on the hot articles (Drossman, 2016; Stanghellini et al., 2016; Enck et al., 2017; Moayyedi et al., 2017; Aziz et al., 2018; Wauters et al., 2020b) with ongoing citation bursts, burstiness of author keyword was used to define foci of research in more depth. Those author keywords with ongoing citation bursts (i.e., acupuncture, gut microbiota, duodenal eosinophilia, mast cell, and anxiety) suggest the topics that prove to be potentially of particular interest. Furthermore, the presented topics were well represented in the results of a comparison of two corpora pertaining to FD extracted from the WOSCC database (one for the period 2017–2019 and the other for 2020–2022). Based on Figure 8 and Supplementary Figures 7, 8, potential future hot research topics have been determined.

As previously stated, the pathophysiology of FD is likely multifaceted, and a variety of classic pathophysiologic mechanisms have been linked in certain dyspeptic symptoms. These proposed themes in the pathophysiology include gastric hypersensitivity, dysregulated gastric motor function (e.g., gastric dysaccommodation and delayed gastric emptying), increased sensitivity to chemicals or to gastric distension, *H. pylori* infection, as well as dietary, environmental, and psychological factors. Current treatment options for these pathways are limited to chronic symptom relief with suboptimal efficacy. This contributes to a high disease burden, diminished QOL, and financial hardships. Further research on the pathophysiology of FD is therefore necessary to provide more precise targets for future therapies and novel treatments are desperately needed for this condition.

Figure 8 and Supplementary Figures 7, 8 demonstrated that prevailing pathogenesis-associated author keywords were related to duodenal abnormalities such as altered microbiota, increased mucosal permeability, infiltration and activation of eosinophils and mast cells, and psychiatric co-morbidities. It is therefore possible to attribute FD’s pathogenesis to alterations in the duodenal luminal and microbiota that interact with the mucosal immune system to elicit symptoms, as evidenced by accumulating evidence indicating that specific patient groups may exhibit tangible pathology in the duodenum, including eosinophilia, mast cell proliferation, and neuronal structural changes.

With evidence available, the pathogenesis of FD was further conceptualized as follows: (1) antigen presentation to the duodenal mucosa, which may involve food macromolecules, nutrients or microbial antigens; (2) activation of eosinophils and mast cells *via* an immune cascade; (3) local nerve sensitization and systemic immune activation contribute to FD symptomatology; and (4) maintenance of a low-grade inflammation *via* bidirectional gut-brain and brain-gut axes.

**FIGURE 9:**
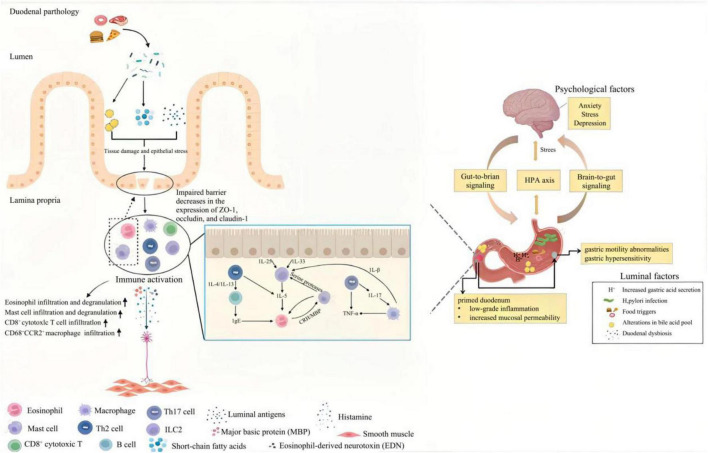
Schematic overview of the evidence regarding immune activation in FD.

Therapeutically, despite the use of numerous therapeutic approaches (such as dietary intervention, antibiotics, acid suppressants, and prokinetics) for the treatment of the various symptoms associated with FD, no single approach has proven to be consistently successful. Also, there are currently no treatments that have been approved by the Food and Drug Administration for the disorder. As depicted in Figure 8, a growing body of research is examining the role of the microbiota, psychological distress, and mood disorders in FD. In light of the recent reclassification of FD as brain-gut regulation disorder and the growing body of evidence demonstrating the crucial role of the gut microbiota in symptom expression, there have been calls for integrated clinical management models that combine conventional medical care with microecological therapy, behavioral, and dietary interventions. Besides, a survey conducted in Italy of more than 200 patients with FGIDs, of whom 82.4% were diagnosed with FD according to Rome III criteria, revealed that 48.7% of patients also used complementary and alternative medicine (CAM) as a supplement to conventional therapy, despite the fact that the majority of patients receive conventional therapy (Lahner et al., 2013). Acupuncture is one of the most frequently used CAM for FD. Based on a meta-analysis of eight randomized controlled trials (RCTs) that compared acupuncture with sham therapy in patients with FD, acupuncture was found to significantly reduce symptoms and gastric emptying time, while having few adverse effects (Mao et al., 2020). Further, these key hot issues in FD research details follow, with their associations emphasized (Figure 9).

#### 4.3.1. Eosinophil-mast cell axis

Visceral hypersensitivity and abnormal gastric motility are thought to directly contribute to FD symptoms, but other factors such as increased gastric acid secretion, infection with *H. pylori*, dysbiosis, psychological disorders, diet, lifestyle, and stomach shape modify those physiological abnormalities and thus modify FD symptoms. Collectively, symptoms of dyspepsia result from the above factors interacting in a complex way.

Evidence is mounting suggesting that these interactions primarily occur in the duodenum, a key organ involved in the pathophysiology of FD, where the aforementioned factors cause symptoms by affecting the organ; the duodenum is thought to be essential for regulating the physiological processes of the stomach, and in FD, gastric malfunction is thought to be secondary to duodenum stimulation, thus causing dyspeptic symptoms.

In light of the idea that gastric dysmotility and gastric hypersensitivity may be the downstream effects of duodenal pathology, recent advances have shown that FD may be caused by changes in the duodenum, such as minimal mucosal inflammation and impaired mucosal integrity (Miwa et al., 2019).

There is no consensus as to whether immune activation precedes barrier damage or *vice versa*, despite the repeated links between the two. Most likely, the two pathophysiological phenomena are amplified by one another, resulting in a vicious circle, making it even more complex to unravel these mechanisms (Ceulemans et al., 2022). The inflammatory infiltrate is likely to release granular components, resulting in barrier impairment. A barrier defect, however, can allow luminal substances, such as microbial components, food antigens, acids, bile acids, and lipids, to invade the intestinal tissue, triggering mucosal immune responses (Wauters et al., 2020b).

In FD, mucosal eosinophils are predominant, with mast cells infiltration often occurring alongside, despite reports of CD8^+^ cytotoxic T lymphocytes and CD68^+^CCR2^+^ macrophages as tissue cell populations (Gargala et al., 2007; Futagami et al., 2010; Burns et al., 2019a). There is therefore low-grade immune activation of the duodenum, which can be observed through elevated amounts and activation of eosinophils and mast cells (clustering and degranulation), resulting in greater duodenal epithelial permeability (Vanheel et al., 2014), challenging the notion that FD is purely psychosomatic. It was further observed that FD was associated with increased duodenal eosinophils in a majority, which do not exclusively represent either subtype of this disorder.

The initial report of duodenal eosinophil infiltration in FD has been presented in pediatric patients from the USA, although there are no controls included in this study (Friesen et al., 2002), as well as in a nested case-control study of Swedish adults (Talley et al., 2007). In this population-based study with 1,001 participants who underwent endoscopy, there was a significant positive correlation between eosinophil infiltration in the duodenum and early satiety, a defining symptom of FD and particularly PDS (Talley et al., 2007). This suggests that duodenal impairments may contribute to meal-related complaints.

Subsequent replication of these results in the UK (Walker et al., 2010) and Australia (Walker et al., 2014) confirmed a preponderance of duodenal eosinophilia in PDS. However, there is a similar prevalence between PDS and EPS in other cohorts (Walker et al., 2009; Vanheel et al., 2014; Tanaka et al., 2016).

In fact, it is estimated that up to 40% of FD patients have micro-inflammation, frequently in the form of eosinophil infiltration (Du et al., 2018). In contrast, few studies have demonstrated changes in stomach immune cell profiles, none of which are associated with symptoms of FD (Futagami et al., 2010; Li et al., 2010; Pasricha et al., 2021), confirming that dyspeptic symptoms do not directly correlate with gastric pathophysiology. Accordingly, this further strengthens the notion that the duodenum is likely to be the key pathogenic organ for FD in that impaired duodenogastric communication is induced by duodenal abnormalities therefore generating FD symptoms (Vanheel and Farré, 2013; Wauters et al., 2020a; Ceulemans et al., 2022). Furthermore, increased mast cells and degranulation was found in the duodenum of FD patients in addition to the eosinophilic infiltration (Vanheel et al., 2014; Cirillo et al., 2015; Taki et al., 2019), although these findings were not always consistent (Tanaka et al., 2016; Puthanmadhom Narayanan et al., 2021), nevertheless substantiating the concept of an eosinophil-mast-cell axis (Wechsler et al., 2021).

Chemokines regulate eosinophil recruitment into the gastrointestinal tract, with eotaxin-1 [C-C motif chemokine ligand (CCL) 11], which is constitutively expressed in the intestinal lamina propria, being one of the most important (Powell et al., 2010). Eotaxin-3 (CCL26) is another powerful eosinophil-recruiting chemokines (Weller and Spencer, 2017). Therefore, the expression of CC-chemokine receptor (CCR)-3, which binds eotaxins, is required for the recruitment of eosinophils to the intestine (Blanchard and Rothenberg, 2009).

As far as immune profiles are concerned, those of the proximal small intestine appear to be predominantly influenced by the T-helper (Th)-2 phenotype (Brown and Esterházy, 2021). IL-5 is a key component of eosinophil biology, as its expression acts as the strongest stimulator among the various Th-2 cytokines required for eosinophil activation (Keely et al., 2015). Further, increased production of IL-5 and IL-13 by stimulated lymphocytes in FD patients is consistent with the profile of a Th2-type immune response (Kindt et al., 2009). In this Belgian study, Kindt et al. (2009), specifically, reported systemic changes in both FD and IBS patients, including increased CD3^+^CD45RA^+^CD45RO^+^ lymphocytes, as compared with the control group, suggesting that naïve T cells may be in transition to a memory phenotype (Kindt et al., 2009). Further, increased levels of IL-5 and IL-13 and decreased interferon-γ (IFN-γ) production following lymphocyte stimulation was reported in the combined FD and IBS cohort when compared to controls (Kindt et al., 2009). A decrease in IL-12 production was also observed following stimulation of monocytes (Kindt et al., 2009). Therefore, there is a shift to a Th-2 cytokine response in FD, such as those observed in atopy and allergy.

Another study, by Liebregts et al. (2011), reported increased proportions of circulating gut-homing T helper cells (CD4^+^α4β7^+^CCR9^+^) and increased production of tumor necrosis factor-α (TNF-α), IL-1β, IL-6, and IL-10, despite no increase in overall Th cell levels, implying immune recruitment to the gastrointestinal tract in these patients. In addition, this study demonstrated that elevated serum cytokines and enhanced lymphocyte recruitment to the small intestine, both of which are indicative of active inflammatory states, were associated with symptoms (upper abdominal pain, cramps, nausea, vomiting, and delayed gastric emptying) in patients with FD (Liebregts et al., 2011).

Mechanistically, a compromised mucosal barrier allows pathogens and food antigens to penetrate the intestinal barrier, leading to a Th2 immune response, which then activates immune responses initiated by mast cells and eosinophils. Upon activation, mast cells and eosinophils further produce pro-inflammatory cytokines and transforming growth factors that contribute to Th1/Th2 polarization (Keely et al., 2015). In parallel, activated eosinophils function as antigen-presenting cells for Th2-lymphocytes with immunoglobulin (Ig) class-switching to IgE in B cells *via* IL-4 or IL-13. Importantly, eosinophils are the most widely recognized and influential effector cells during Th2 responses (Powell et al., 2010). In this regard, Th2-associated IL-5 can activate duodenal eosinophils, and the granular content released by eosinophils, such as major basic protein (MBP), may then act as a signal to activate mast cells. As a reciprocal mechanism, mast cells are also identified as recruiters of eosinophils in FD, when they are activated by IL-4 or IL-13 released from Th2 cells.

Further to this, evidence is also growing that group 2 innate lymphoid cells (ILC2s) may contribute to the recruitment of eosinophils by producing cytokines, including IL-5 and IL-13 (Larose et al., 2017). In terms of mechanism, the loss of duodenal homeostasis is accompanied by the switch from ILC3-mediated homeostasis to ILC2-dominant response (Larose et al., 2017). Expansion of ILC2 populations are therefore activated by cytokines such as IL-33, IL-25, IFN-γ, and IL-1β, as well as serine protease released by mast cells (Larose et al., 2017). In this cascading inflammatory response, IL-5 and IL-13 are secreted by ILC2s, subsequently promoting eosinophilic recruitment and further perpetuating the inflammatory events (Larose et al., 2017). In addition, IL-33 is shown to stimulate mast cell activation, which in turn triggers the recruitment of eosinophils (Larose et al., 2017).

A link between FD and atopy and allergy can, therefore, be explained by the presence of duodenal eosinophils (Wauters et al., 2020a); although the hypothesis has been proposed in the literature, there is no direct evidence that the duodenal eosinophilia observed in FD patients is due to a type-2 immune response (Burns et al., 2019a).

In contrast, increased levels of TNF-α and IL-1β production point toward a Th17-driven response, as IL-17 has been shown to trigger the production of these cytokines from human macrophages (Liebregts et al., 2011). Eosinophils are responsible for regulating Th17 responses (Sugawara et al., 2016). Furthermore, pre-clinical studies have shown that intestinal eosinophil recruitment involves IL-17-dependent mechanisms, implying that FD-associated eosinophilia may occur independently of type-2 responses (Griseri et al., 2015). In light of recent reports of increased mucosal Th17 density in pediatric FD patients with chronic gastritis (Singh et al., 2020), it may be possible to explain at least some of the symptoms of this condition by Th17 cytokine responses (Keely et al., 2015; Burns et al., 2019a). The most recent work by Burns et al. (2023) shows that FD patients have elevated Th2- and Th17-like cells localized to the duodenum, implying that dual lymphocyte response pathways are implicated in the generation of FD symptoms. In addition, the existence of effector and memory cells indicates that the microinflammation in FD is antigen driven (Burns et al., 2023). Taken together, the interplay between Th2- and Th17-signaling and ILC2-mediated inflammatory signals may be hallmarks of immune activation profile in the text of FD (Keely et al., 2015).

In addition to the stimulation of mast cells by MBP derived from eosinophils, IL-4 and IL-13 released by Th2 cells may also stimulate mast cell activation in a manner similar to that of eosinophils, resulting in high levels of mediators released, such as histamine, substance P, and tryptase (Powell et al., 2010). Consequently, a co-accumulation of eosinophils and mast cells is prevalent in disorders of gut-brain interaction, lending credence to the idea of an eosinophil-mast cell axis.

With growing interest in the duodenum, research on duodenal immune dysfunction in FD, as indicated by increased eosinophil and mast cell infiltration and degranulation, has confirmed its association with FD symptoms such as early satiation, postprandial fullness, and abdominal pain (Wang et al., 2015; Lee et al., 2019; Järbrink-Sehgal et al., 2021).

But how does the low-grade inflammation augment mucosal permeability and cause physiological abnormalities in the stomach (i.e., gastric motility dysfunction and gastric hypersensitivity) that directly result in dyspeptic symptoms?

Visceral hypersensitivity is a crucial factor in FGIDs, greatly influencing the occurrence and severity of symptoms. This is due to abnormal mucosal immune response activation in relation to the level of inflammatory response and the close proximity of immune cells and sensory neurons.

In particular, Tanaka et al. (2016) found increased glial cell line-derived neurotrophic factor expression in eosinophils and a correlation with epigastric burning in patients with FD. Additionally, eosinophils may interact with neuronal signaling *via* neurotrophins that they produce, such as brain-derived neurotrophic factor and nerve growth factor (Cirillo et al., 2015; Ceulemans et al., 2022). The neuro-immunological perspective thus adds to the understanding of the mechanism of gastrointestinal symptoms associated with immune activation, as seen in FD. Furthermore, cytotoxic granular proteins produced by eosinophil degranulation, such as MBP and eosinophil peroxidase, can act as antagonists of the muscarinic M2 receptor, potentially causing smooth muscle dysfunction, which is linked to disturbed intestinal motility (Walker et al., 2010; Zala et al., 2015). The mechanistic association between these immune cells, intestinal epithelium, and various nerves may therefore cause increased mucosal permeability and altered smooth muscle contraction, leading to gastroduodenal hypersensitivity and motor dysfunction, which are directly associated with FD symptoms.

In addition, since the findings reveal altered expression of proteins that comprise the cell junction, the paracellular pathway is likely to be involved in barrier dysfunction in FD. Trans-tight junction conductance occurs *via* two distinct pathways: pore and leak (Shen et al., 2011). There is thus evidence that tight junction dysregulation contributes to increased intestinal permeability, loss of intestinal barrier, and disorders of gut-brain interaction, including FD. The pathophysiology of FD is likely to be influenced, more specifically, by the paracellular leak pathway as opposed to the pore pathway, according to recent literature.

There is a paucity of research on the specific pathways that are responsible for the altered permeability seen in FD; however, it is likely that duodenal immune activation will occur as a result of and contribute to changes in duodenal permeability since eosinophils and mast cells have been linked to both epithelial cell damage and repair (Komori et al., 2019). Duodenal eosinophil counts were also correlated with the protein expression of intercellular tight junctions, including zonula occludens-1, occludin, and claudin-1 (Vanheel et al., 2014; Nojkov et al., 2020); these tight junctions have also been associated with altered gene expressions in the duodenum, including IL-1β and IL-6 (Stenfeldt and Wennerås, 2004), but a more comprehensive understanding of the cause-and-effect relationship between duodenal barrier defect and abnormal immune activation is still awaited.

Additionally, the promotion and sprouting of fine nerve fibers in the duodenum, a condition more prevalent in patients with EPS, was linked to the accumulation of mucosal eosinophils with degranulation (Lee et al., 2019). According to another study in FD patients, eosinophils were found to be more abundant near submucous plexus neurons and were associated with structural abnormalities (gliosis, altered ganglionic architecture, and neuronal abnormalities) as well as functional impairments (lower calcium response to depolarization and electrical stimulation) of the submucous nerve plexus, thereby affecting neuronal and muscular function (Cirillo et al., 2015). Since the submucous plexus is responsible for mediating local gut contractions and reflex responses (Fleming et al., 2020), it is possible that inflammatory mediators released by eosinophils contribute to abnormal stimulation of submucous plexus neurons, leading to altered duodenogastric feedback and, ultimately, results in the manifestations of FD, including disturbances in gastric motility and symptoms associated with it. The results of both studies thus provide further evidence that duodenal immune activation in FD is linked to altered submucous neuronal responsiveness, neuronal structural abnormalities and sensitization of the afferent nerves (Cirillo et al., 2015; Lee et al., 2019).

The expanding knowledge of immune activation in FD has not yet led to significant advances in anti-inflammatory medications targeting eosinophil infiltration. It is recommended that PPIs be used as the first line of treatment in accordance with consensus recommendations from North America, Europe, and Japan (Moayyedi et al., 2017; Wauters et al., 2021b; Miwa et al., 2022).

The expression of vascular cell adhesion molecule-1, which is recognized by eosinophil ligands, has been shown to be inhibited by the use of PPIs (Barthel et al., 2006), providing an explanation for the response of eosinophilia in an acid-independent manner. In addition, the release of IL-5 by Th2-lymphocytes can activate eosinophils *via* eotaxins through a mechanism involving the activation of signal transducer and activator of transcription 6 (STAT6) pathway (Zimmermann et al., 2003). It has been demonstrated that omeprazole prevents STAT6 from binding to the eotaxin-3 promoter, which in turn inhibits the expression of eotaxin-3 by esophageal cells in eosinophilic esophagitis (EoE) (Cheng et al., 2013). Even though these mechanisms are implicated in EoE, which is characterized by a substantial inflammation profile where there are an increased number of eosinophils present, certain mechanisms associated with eosinophil infiltration may also exist in FD; therefore, similar mechanisms may be at work in the duodenum of FD patients when taking PPIs for their anti-eosinophilic effects (Min et al., 2017).

PPI therapy was also evaluated clinically for its effect on suppression of duodenal eosinophilia in 20 FD patients in a case-control study (Potter et al., 2019). Patients who took PPIs had a lower descending duodenal eosinophil count than those who did not (*p* = 0.03) despite there being no difference between the groups in the eosinophil counts in the duodenal bulb or in gastric biopsies (Potter et al., 2019).

Furthermore, Wauters et al. (2021a) reported the first prospective evidence for PPIs reducing duodenal eosinophils. The study found that treatment with pantoprazole reduced the number of mast cells and duodenal eosinophils in patients with FD, along with the ameliorated intestinal permeability as well as severity of symptoms (Wauters et al., 2021a). Moreover, clinical efficacy was associated with reduced mucosal eosinophil counts in these patients (Wauters et al., 2021a). Due to the fact that this effect was not mediated by changes in duodenal pH, it appears to be a direct impact on inflammation, free of PPI’s acid suppressant properties (Wauters et al., 2021a). FD patients also showed duodenal changes that were not reversed once long-term PPI was withdrawn, suggesting that persistent changes may be at play (Wauters et al., 2021a).

In dyspeptic patients, gastric acid secretion is within the normal range (Collen and Loebenberg, 1989). It is not completely clear how acid-suppressive drugs ease dyspeptic symptoms when GERD and FD do not overlap significantly. The available evidence suggests that PPIs alleviate symptoms by suppressing eosinophils, which indeed supports the central role played by eosinophils in fueling and maintaining low-grade inflammation and altered brain-gut communication, thus in turn generating symptoms in FD.

Furthermore, other anti-inflammatory therapies for gastroduodenal disorders other than FD should be considered, as they may support the transferability of these results to FD patient cohorts. An excellent illustration of this is budesonide, which is efficient in treating diseases of the proximal small intestine, such as refractory celiac disease (Mukewar et al., 2017). In a pioneering study of assessing anti-inflammatory therapies for FD, Talley et al. (2021) conducted a randomized, double-blind, placebo-controlled trail in adults with FD and mucosal eosinophilia using steroids that are also known to be effective in treating EoE accompanied by a reversible over-expression of eotaxin-3 and IL-13 (Blanchard et al., 2007). Although there was no significant difference in symptomatic response between the treatment and placebo groups, a decrease in duodenal eosinophil counts from pre- to post-treatment was linked to improved postprandial fullness and early satiety (Talley et al., 2021). Although this study failed to demonstrate efficacy of steroids in the treatment of FD, it shows that duodenal immune dysfunction is linked to gastroduodenal symptoms.

Other anti-inflammatory treatments that target duodenal eosinophil recruitment and activation, such as monoclonal antibodies against Th2-derived cytokines (Stein et al., 2006; Straumann et al., 2010; Rothenberg et al., 2015; Hirano et al., 2020), anti-α4β7 integrin antibody (Bochner, 2000; Grandinetti et al., 2019), and Janus kinase/STAT6 pathway inhibitors (Cheng et al., 2016), are also anticipated for FD patients.

#### 4.3.2. Gut microbiota

As a third component of the “gut-brain” axis, microbiota has been actively researched. The luminal contents, although separated by the intestinal barrier, are influential on mucosal homeostasis and immune activation, with the gut microbiome being an important factor (Peterson and Artis, 2014). It has been suggested that FD patients exhibit low-grade duodenal and systemic inflammation, specifically increased duodenal permeability and eosinophilia (Talley et al., 2007; Liebregts et al., 2011); hence, altered gut microbiota are relevant to these processes (Vanheel et al., 2014).

There is evidence that FD is associated with alterations in microbial communities throughout the gastrointestinal tract, not only at one location. There was a significant difference in the composition and total number of microbiota in gastric fluid of patients with FD compared to healthy subjects (Nakae et al., 2016). When compared to healthy subjects, there was a significant decrease in *Prevotella* abundance and an increase in *Bifidobacterium* and *Clostridium* abundance (Nakae et al., 2016). Furthermore, a negative correlation was found between the relative abundance of *Prevotella* and the severity of PDS symptoms (Nakae et al., 2016).

In addition, one study demonstrated that gastric fluid samples from patients with FD lacked *Acidobacteria*, and a higher ratio of *Bacteroidetes* to *Proteobacteria* was observed (Igarashi et al., 2017). Despite the ambiguity as to what constitutes a healthy microbiome, gastric fluid from healthy individuals normally contains *Acidobacteria* and typically has a low ratio of *Bacteroidetes* to *Proteobacteria* (Igarashi et al., 2017).

Mechanistically, gastric fluid has a higher species richness, which may indicate that the microbes present, as well as toxic bacterial components and metabolic mediators such as lipopolysaccharides, induce leukocytes to produce pro-inflammatory cytokines, leading to gastric inflammation and, potentially, increased mucosal permeability (Rupp and Stengel, 2022). The result could be a malfunctioning enteric nervous system manifesting as a disturbance in gastric motility (Rupp and Stengel, 2022). However, the gut microbiota is linked to immune cells, enteroendocrine cells, and the enteric nervous system *via* a complex circuit system that operates in a coordinated but still unclear fashion. In addition, as previously discussed, there have been very few studies reporting changes in gastric immune cell subpopulations, and none of these have been linked to FD symptoms (Futagami et al., 2010; Li et al., 2010; Pasricha et al., 2021). Given the preceding discussion, additional research is required to determine if the gastric fluid microbiota is sufficiently large and diverse for bacterial components and bacteria-derived molecules to affect the stomachs of FD patients, causing inflammation and mucosal permeability.

Further, in the FD group, luminal *Streptococcus* was found at higher abundance in the oral cavity, esophagus, stomach, and duodenum than in the control group, and its relative abundance was positively associated with upper gastrointestinal symptoms in both the PDS and EPS subtypes (Fukui et al., 2020). FD patients were also found to exhibit *Streptococcus* as the predominant genus within the duodenal mucosa (Zhong et al., 2017). Moreover, microbial load was found to be positively related to the severity of meal-related FD symptoms, but negatively correlated with QOL (Zhong et al., 2017). Despite the inconsistency of microbiota studies, which is largely due to differences in methodology used in assessing the microbiome as well as differences in sampling sites and sequencing targets, it appears that there is a consensus that a bacterial overgrowth of *Streptococcus* species (bacterium with acid producing abilities) is present in FD patients (Paroni Sterbini et al., 2016; Zhong et al., 2017; Fukui et al., 2020).

One example of gut microbiota abnormalities in the small bowel is a condition known as SIBO, which is defined as an increased concentration of small intestine bacteria as measured by quantitative culture (at least 10^5^ colony-forming units per milliliter of jejunal aspirate) (Ghoshal and Ghoshal, 2017). An association between SIBO and the FD has been demonstrated in several studies (Shimura et al., 2016; Tziatzios et al., 2021; Chuah et al., 2022). In addition, a recent systematic review and meta-analysis revealed that patients with FD are more likely to suffer from SIBO detected by breath tests than healthy controls, with no significant differences in SIBO prevalence between subtypes of FD (Gurusamy et al., 2021). These studies point to a potential link between dysbiosis and the pathogenesis of FD, with SIBO possibly being associated with the onset or worsening of symptoms in a subset of FD patients. However, there is no evidence that dysbiosis and FD are causally related to one another, and the specific microbiome that is pathognomonic for FD is also unknown.

Although FD microbial changes are not restricted to a single region in the gastrointestinal tract, low-grade duodenal inflammation may result from alterations to the gut microbiota of the small intestine. Future studies may benefit from this hypothesis. In particular, the duodenal microbiota is physiologically necessary for the maintenance of small intestinal digestive functions because it is responsible for the fermentation of food and the release of digestive enzymes such as bile salt hydrolase (Urdaneta and Casadesús, 2017; Duncanson et al., 2021). However, total dietary fat consumption and dietary fat profile alter the relative abundance and diversity of duodenal bacteria due to their role as substrate, with a high-fat diet associated with decreased Bacteroidetes and increased Firmicutes (Sandhu et al., 2017). Diet, therefore, is closely associated with the microbiota, contributing to immune and microbial interactions in FD. In fact, FGIDs, such as FD, were significantly more prevalent in obese compared to normal weight patients (Tambucci et al., 2019).

As microbiota, and thereby their functional repertoires are altered in the context of FD, the changes in the short-chain fatty acid (SCFA) profile, products of fermentation as well as the pool of bile acids follow (Pavlidis et al., 2015; Wei et al., 2021). As a result, these changes in bile acid composition may, in turn, promote alterations in gut microbial diversity, resulting in epithelial stress and damage (Banerjee et al., 2016; Beeckmans et al., 2020).

The link between bile acid biology and FD pathophysiology suggests that duodenal microbiota-mediated bile acid signaling might be involved. It has been observed that reduced duodenal primary bile salt amounts in FD are associated with decreased intestinal barrier function (Beeckmans et al., 2018, 2020). The mechanism for this phenomenon is thought to involve overgrowth of pro-inflammatory bacteria as a result of reduced primary bile salt concentrations in the duodenum, leading to cellular stress responses and barrier dysfunction, which also contributes to low-grade inflammation (Kakiyama et al., 2013; Pavlidis et al., 2015). Duodenal epithelial barrier dysfunction could occur as a result of these changes.

In addition to this, changes in bile acid may be the link between diet, the microbiota, and leaky gut, all of which may be factors that contribute to the development of FD (Keely and Talley, 2020). For example, excess dietary fats are likely to increase intestinal permeability through a variety of mechanistic pathways, including the modification of tight junction expression and distribution, the promotion of the transition to barrier-disruptive hydrophobic bile acids, and the induction of oxidative stress and apoptosis in enterocytes (Duncanson et al., 2021). Also, increased gut permeability associated with a high-fat diet may also result from the activation of pro-inflammatory signaling cascades directly, as well as indirect mediators, such as increased barrier-disruptive cytokines, decreased barrier-forming cytokines, negative modulation of intestinal mucus composition, and enrichment of gut microbiota with barrier-disruptive properties (Rohr et al., 2020). In general, disruption of bile acid-microbiota-epithelial barrier homeostasis is a crucial mechanism underlying FD pathophysiology.

The dysbiotic signature relating to symptomatology has not yet been definitively identified despite microbial changes being implicated in FD pathogenesis. Research is still underway to determine exactly how the duodenal dysbiosis, duodenal eosinophilia, and barrier dysfunction interact in FD. A recent study found that decreased duodenal mucosal *Neisseria* and *Porphyromonas* abundance in FD patients is associated with symptoms and duodenal eosinophils, implying a link between duodenal dysbiosis and immune activation (Wauters et al., 2021d). Inflammatory immune responses and microbial changes may lead to the dysfunctional regulation of cellular stress response pathways, disrupting gut homeostasis and causing loss of mucosal integrity, which in turn leads to the recruitment of eosinophils and the perpetuation of symptoms (Burns et al., 2019b). Furthermore, the studies summarized above highlight the importance of further research into the effects of primary and secondary bile salts on duodenal permeability, as well as the potential role of bile salts in the mucosal immune profile of FD (Beeckmans et al., 2020).

Aside from compositional imbalances, there may be a metabolomic dysregulation as well. Among the key functions of the gut microbiota is the processing of indigestible components of polysaccharides, producing SCFAs such as butyrate, which has anti-inflammatory properties (Silva et al., 2020). In colitis models, SCFAs have demonstrated beneficial effects by alleviating mucosal inflammation (Vieira et al., 2012); other studies have linked the SCFA profile with specific IBS subtypes as well as the disturbance of gut motility (Hurst et al., 2014; Dalile et al., 2019; Shaidullov et al., 2021). In light of the paucity of literature regarding the role of SCFAs in FD, the profile of SCFA in FD duodenum has yet to be defined. Nonetheless, evidence from IBS or colitis-related data, along with others, give a clue that altered SCFAs may, to some extent, be responsible for gut motility disturbances, increased duodenal permeability, and immune cell infiltration in FD.

There are no conclusive studies that have established whether dysbiosis itself is the cause or merely an outcome of the FD pathological process, but several studies have pointed to probiotics as a possible treatment alternative.

As reported by Igarashi et al. (2017), a markedly different bacterial composition in gastric fluid was found between FD patients and healthy controls, and the shift in the microbiota analysis of FD patients was restored to that seen in the control group after treatment with the probiotic *Lactobacillus gasseri* OLL2716 (LG21 strain). In this study, a probiotic product containing *Lactobacillus* strains was used, which produce SCFAs including acetate, propionate and butyrate (Markowiak-Kopeć and Śliżewska, 2020). In a RCT using LG21 strain in *H. pylori* negative FD, continuous intake of LG21 strain resulted in symptom resolution in PDS rather than EPS (Ohtsu et al., 2017). Therefore, LG21 may exhibit its beneficial effects mainly in conditions associated with abnormal gastric motility such as delayed gastric emptying and gastric dysaccommodation (Ohtsu et al., 2017).

In addition, a recent study showed that *Bacillus* spores were more effective than placebo in treating FD adults (Wauters et al., 2021c). Probiotic therapy may be beneficial for immunomodulation and microbial regulation in FD as patients taking spore-forming probiotics more frequently achieved clinical endpoints with decreased pro-inflammatory IL-17 and Th17 cytokines and increased concentrations of *Faecalibacterium* and *Roseburia* in stools (Wauters et al., 2021c). In addition to providing evidence for the positive effects of probiotics on symptom severity, microbiome composition, and immune regulation, additional research indicates that probiotic consumption is associated with the modulation of microbial metabolites, resulting in the increase of beneficial intestinal metabolites, such as pelargonic acid, benzoic acid, and SCFAs, and the decrease of harmful intestinal metabolites, such as hippuric acid (Sun et al., 2021).

It is currently unknown what exact mechanism may be responsible for the potential beneficial effects of probiotics on FD symptoms; however, SCFAs are hypothesized as potential mediators. As mentioned above, the addition of probiotics to FD may enrich beneficial bacteria or SCFA-producing bacteria, thereby restoring SCFA production (van Zanten et al., 2012; Sun et al., 2021). By virtue of their immunological, endocrine, vagal, and other actions, SCFAs have also been shown to modulate gut-brain interactions, as well as ameliorate gut-barrier injury, inhibit intestinal inflammation, and regulate gut motility (Ropert et al., 1996; Lewis et al., 2010). Given limited studies investigating these effects in humans, probiotic-mediated resolution of microbial dysbiosis through SCFAs and the related immune modulation in FD remains a hypothesis.

Further, it is interesting to note that in a recent study that analyzed the microbial profiles of FD patients, no distinct differences were observed between them and their healthy control counterparts (Vasapolli et al., 2021). Though, it is noted that the effects of PPI and prokinetic use, dietary modifications, and delayed gastric emptying on the microbiota composition in the stomach and duodenum of FD patients cannot be completely ruled out. Overall, the evidence presented thus far points to a link between FD and dysbiosis; additional research is necessary to gain a deeper understanding of the pathogenic microbiome and its mechanisms, as well as the mechanisms underlying probiotics’ potential beneficial effects. Further clues might be provided by the duodenal microbiota-mediated bile acid signaling and metabolites resulting from diet-microbiota interactions.

#### 4.3.3. Mental disorders

Psychological co-morbidities, such as stress, anxiety, or depression, are linked to FGIDs and contribute to the pathophysiology of the disorder. The prevalence of anxiety and depression has been found to be higher among those with FD, although the estimates of the co-morbidities are highly variable (Li Y. et al., 2002; Hartono et al., 2012). The results of a study in Sweden, which followed up 887 participants for 10 years, indicated that anxiety at baseline, but not depression, was associated with the newly onset FD (Aro et al., 2015). As evidenced by these observations, the pathogenesis of FD is strongly influenced by mental disorders. Further studies indicate that mental disorders and psychosocial factors may contribute to FD through modulation of cerebral processing of visceral afferent signals and pain perception mediated by stress hormones (Van Oudenhove and Aziz, 2013; Hannibal and Bishop, 2014).

The altered glycometabolism was studied in 40 FD patients and 20 controls during resting state (Nan et al., 2015). In FD patients, glucose metabolism in the insula, anterior cerebral cortex, middle cingulate cortex, and middle frontal cortex was higher after controlling dyspepsia (Nan et al., 2015). Among the subjects, the abnormalities were positively correlated with anxiety and depression scores, which suggests that the altered cerebral glycometabolism might result from the vicious cycle of psychopathology and gastrointestinal symptoms. Psychological vulnerabilities may affect glucose metabolism in these homeostatic afferents and sensory areas beyond simply influencing visceral afferent signaling (Nan et al., 2015). Also, other studies that used fluorodeoxyglucose positron emission tomography to assess resting brain glucose metabolism in FD patients found higher levels of metabolism in various brain regions thought to be involved in pain modulation circuits, including the anterior cingulate cortex, the insula, and the thalamus compared to healthy subjects (Zeng et al., 2011). However, these differences were not associated with anxiety levels, but rather with symptom severity (Zeng et al., 2011).

Furthermore, while not specifically identified in FD patients, psychological variables and stress hormones have been demonstrated to influence gastrointestinal motility, mucosal immune activation, intestinal permeability, and microbiota (Molina-Torres et al., 2019). Taken together, psychosocial disorders, such as anxiety and mood disorders, were found to be independently associated with the occurrence of FD and PDS, which suggests top-down communication between the brain and gut (Aro et al., 2009; Jones et al., 2017).

Alternatively, the symptoms of FD or the disease burden itself are thought to cause anxiety or depression as a result of low-grade intestinal inflammation. It has been noted that patients with high baseline anxiety levels are more likely to develop new onset gastrointestinal symptoms; additionally, patients with FD or IBS and no mood disorders at baseline were more likely to develop anxiety or depression symptoms (Koloski et al., 2016). A study conducted by Koloski et al. (2016) has suggested that mood disorders precede FGID in one-third of patients, whereas FGID precedes mood disorders in two-thirds, indicating that bottom-up pathway signaling is relevant to a subset of these patients.

A number of different pathways are involved in the interaction of the gut microbiota with the central nervous system, which could interfere with brain function *via* possible neurological (vagus nerve and spinal cord), endocrine [hypothalamus-pituitary-adrenal axis (HPA)], metabolic (SCFAs, bile acids, and others), and immunological (cytokines) pathways (Cryan and Dinan, 2012). This suggests that mental disorders may be the result of dysbiosis and, as such, might be contributing to the development of FD.

Furthermore, an increased number and rate of degranulation of mast cells in the duodenum is associated with anxiety and depression in FD (Yuan et al., 2015). In addition, a 10-year study found a correlation between baseline duodenal eosinophilia and anxiety at follow-up, suggesting that gastroduodenal inflammation may contribute to psychiatric symptoms in patients with gut symptoms (Ronkainen et al., 2021). However, to verify the bottom-up model of FD based on the view of duodenal subtle inflammation, large prospective controlled studies are required, since the limited number of patients with increased duodenal mucosa infiltration by mast cells and eosinophils has prevented firm conclusions regarding the interaction between symptoms, duodenal low-grade immune activation, and psychological distress (Wauters et al., 2021e).

It is also suggested that, apart from psychosocial factors such as anxiety and depression, activation of the HPA axis may bridge gut-brain-axis by producing both gut-derived and central mediators that induce stress, which, in turn, produces symptoms associated with FD (Ceulemans et al., 2022). There is evidence that stress may negatively affect mental health in a variety of ways. For its role in FD, occupational stress or burnout was found to be associated with FD among female employees (Nam et al., 2018). Additionally, patients with FD had higher levels of stress compared with healthy controls; there was also a positive correlation between preceding and concurrent stress and the severity of fullness, a cardinal symptom of the FD (Klaassen et al., 2022). The authors conclude that elevated stress levels may precede fullness sensations in patients with FD (Klaassen et al., 2022).

Hyperactivity of the HPA axis plays a role in the interaction between stress and immune activation. The HPA axis is activated in response to stress-related events, which is indicated by an increase in cortisol, adrenocorticotropin, and corticotropin-releasing hormone (CRH) production (Barden, 2004). It is important to note that CRH is not only produced by the hypothalamus during chronic restraint stress (Zheng et al., 2009). Intestinal eosinophils produce CRH and substance P locally, which activate mast cells and, consequently, increase intestinal permeability (Zheng et al., 2009). The release of CRH by eosinophils is stimulated by psychological stress, possibly through stimulation of the neurokinin receptor 1/2 purinergic receptors located on eosinophils by substance P derived from nerve endings. Further, these stress-induced changes in mucosal permeability have been linked to the eosinophil-mast cell axis in other studies (Wallon et al., 2008; Vanuytsel et al., 2014). This is demonstrated by peripheral administration of CRH, which activates both eosinophilic and mast cell receptors, increasing the permeability of the small intestine (Wallon et al., 2008; Vanuytsel et al., 2014). By pre-treating with cromoglycate, a mast cell stabilizer, the effect can be blocked (Wallon et al., 2008; Vanuytsel et al., 2014). Collectively, enteric permeability can be altered by stress-induced signaling between the central nervous system and immune activation or, more specifically, mucosal mast cells (Söderholm and Perdue, 2001; Teitelbaum et al., 2008; Rodiño-Janeiro et al., 2015). In support of the notion that a mast cell-mediated mechanism may increase mucosal permeability in response to psychological stress, Vanuytsel et al. (2014) conducted a study where healthy volunteers were exposed to psychological stress (public speeches), and the effects of this psychological stress on intestinal mucosal permeability were measured using lactulose-mannitol ratios. In response to this type of psychological stress, mucosal permeability increased (Vanuytsel et al., 2014). Moreover, the study showed that CRH administration increased mucosal permeability and that the mast cell stabilizer cromoglycate reduced the mucosal permeability increases caused by both speech and CRH (Vanuytsel et al., 2014).

It has recently been shown by Wauters et al. (2021a) that patients with FD exhibit higher subjective stress levels and salivary cortisol levels than controls. Additionally, patients with FD showed reduced awakening cortisol levels after taking PPIs; these changes were associated with PPI-related duodenal eosinophil reduction, but not with a decrease in mucosal hyperpermeability (Wauters et al., 2021a). There were, however, no associations between PPI-related changes in mast cells and cortisol in FD, and the reduction in higher awakening cortisol was observed in the absence of subjective stress-level changes (Wauters et al., 2021a). Accordingly, the exact role of mast cells in the increased mucosal permeability induced by stress is yet enigmatic.

Overall, a valuable insight is provided by these findings, which demonstrate that in response to psychological stress, the CRH-eosinophil-mast axis is activated, resulting in degranulation and the release of potentially damaging pro-inflammatory mediators such as tryptase, histamine, serotonin, and others, which potentiate duodenal low-grade inflammation and impaired mucosal barriers. Thus, the hyperactivity of the HPA axis also provides a pathway, which allows infiltrating antigens or components of microbiota to penetrate the epithelial barrier and influence immune cells and neurocytes directly through the intestinal mucosa, establishing a critical link between gut microbiota and brain function and leading to further stress induction (Teitelbaum et al., 2008). The suppression of stress-induced low-grade inflammation of the mucosa as evidenced by the infiltration and degranulation of eosinophils or mast cells could, therefore, be a therapeutically useful approach for restoring homeostatic brain-gut axis to these sufferers, as it may break the vicious circle of gut-brain communication resulting from stress stimulation by targeting pro-inflammatory signaling pathways in the intestine.

In addition, the vagus nerve’s role in chronic stress response cannot be neglected. There is evidence that stress can reduce the activity of the vagus nerve, which may contribute to gastrointestinal inflammation (Taché and Bonaz, 2007; Bonaz et al., 2016). It has been suggested that the vagus nerve’s efferents provide an anti-inflammatory effect in the gut as well as ameliorate the increased intestinal permeability, both of which might be linked to vagal activity mediated reinforcement of tight junctions (Zhou et al., 2013; Van Houten et al., 2015), although further research is needed to determine the exact mechanism. This suggests that reduced vagal activity induced by stress may have a weaker protective effect on the epithelial barrier, increasing epithelial permeability and, as a result, promoting bacterial translocation (Rupp and Stengel, 2022). In this regard, targeting vagal tone in FD patients with high stress levels may also have a positive effect on regulating brain-gut interactions (Bonaz et al., 2018).

The activation of the duodenal eosinophil-mast cell axis has been highlighted in emerging studies of subtle duodenal pathology in FD patients. It is still debated, though, how infiltrating eosinophils and mast cells contribute to increased intestinal mucosal permeability. A decrease in transepithelial electrical resistance and an increase in passage of a paracellular probe associated with reduced expression of tight junction proteins were observed in healthy volunteers after 30 min of acid perfusion of the duodenum (Vanheel et al., 2020). Additionally, duodenal acid perfusion may activate mast cells, as seen by enhanced tryptase expression (Vanheel et al., 2020). However, pretreatment with cromoglycate fail to abrogate acid-induced epithelial barrier impairment, suggesting that the compromised integrity after acid exposure is caused by acid exposure itself rather than by a mast cell-dependent event (Vanheel et al., 2020). Eosinophils in colonic mucosa, however, have been shown to express muscarinic acetylcholine receptors, releasing CRH when stimulated by cholinergic stimulation, disrupting intestinal epithelial barrier function by paracrinely acting on mast cells, whereas activated eosinophils alone had no effect on epithelial permeability (Wallon et al., 2011). Although these mechanisms have not been specifically studied in patients or animal models of FD, discrepancies in the association between immune activation and barrier dysfunction may be explained by the fact that barrier dysfunction may be secondary to other pathological process, such as luminal substances (e.g., changes in enteric bacteria, SCFA profile, and bile acid pool), in addition to factors released by the inflammatory infiltrate (Xu et al., 2014). Further research in the context of FD may be spurred by the potential triad of mental disorders, duodenal immune cell infiltrates, and barrier defects.

As previously mentioned, FD patients may initially develop mental disorders during the course of their illness. Thus, neuromodulators and phytotherapy may be of benefit to these patients. In an updated meta-analysis of RCTs of all drugs for FD, it was concluded that, compared with placebo, antipsychotics showed greater efficacy, followed by pregabalin and tricyclic antidepressants (Ford et al., 2021; Black et al., 2022). In this analysis, serotonin norepinephrine reuptake inhibitors, selective serotonin reuptake inhibitor, and serotonin-1A agonists did not provide any benefit (Black et al., 2022).

Furthermore, a wide range of psychological therapies have been investigated, but the four main modalities studied in FD are psychodynamic-interpersonal therapy, cognitive behavioral therapy, stress management and mindfulness, and gut-directed hypnotherapy (Black et al., 2022). Despite promising results for patients with FD in terms of decreased psychological distress, improved gastrointestinal symptoms, and enhanced QOL, the evidence base for these treatment approaches is scant, which makes it impossible to recommend any particular modality over another (Black et al., 2022).

Regarding the mechanisms by which psychological therapies benefit patients with FD, there is a hypothesis that psychotherapy alleviates mental disorders by altering the microbiome or modulating vagal tone (Rupp and Stengel, 2022), but further research is required to examine these intriguing effects of psychotherapy specifically on patients with FD.

There may be a pre-existing gut-driven syndrome in some FD patients that is related to the onset of brain-related symptoms, therefore necessitating a different treatment approach; however, this strategy is still debated. As discussed previously, two factors may be implicated in the pathogenesis of gut-to-brain bottom-up models: one is dysbiosis, and the other is activation of duodenal mast cells and eosinophils.

From the perspective of dysbiosis, although studies abound repeatedly demonstrating the efficacy of probiotics in improving mental health, leading to the coinage of the term “psychobiotics,” they are not specifically studied in the context of FD (Liu et al., 2019; Ansari et al., 2020). Fecal matter transplant (FMT) is also a proven method for restoring a balanced microbiota and may be a suitable treatment option for intestinal dysbiosis (Khoruts and Sadowsky, 2016). Still, it is unclear whether FMT treatment for FGIDs is a panacea or merely a placebo (Pulipati et al., 2020; Shanahan et al., 2021). Further, there is a paucity of FD-related data investigating whether FMT may pose potential therapeutic gains for patients with FD-associated dysbiosis and, simultaneously, mitigate or even prevent common mental disorders in those with FD.

Furthermore, an RCT of 86 FD patients without SIBO found that rifaximin provided significantly more relief of postprandial fullness or bloating than placebo; however, psychological co-morbidities were not assessed (Tan et al., 2017).

Nevertheless, little is known about this entity that lies at the intersection of FGIDs, depression, and anxiety. At least some evidence indicates that co-morbid patients cluster differently than those with FGID, depression, or anxiety alone (Zhang T. et al., 2022b). In this regard, the treatment strategies to ameliorate gut microbiota dysbiosis that is associated with improved mental function needs to be further examined with regards to whether these have been studied specifically among patients with mental disorders, co-morbid FD patients, or simple FD patients, as variability in symptom and subgroup characterization can skew interventional trial results and lead to falsely attributed positive effects to other patient cohorts. It is therefore paramount, not only to demonstrate a specific microbial signature for FD or mental disorders, but also to well characterize the gut microbiota species in patients who have both FD symptoms and established psychological co-morbidities.

The bottom-up model also allude to eosinophilic duodenitis and increased duodenal mast cells as therapeutic targets for improving mental health and psychological function. Thus, anti-inflammatory (Dellon et al., 2020; Wauters et al., 2021a) and anti-allergy therapies (Friesen et al., 2006; Potter et al., 2020) that target these immune infiltrates and demonstrate promising therapeutic efficacy in FD patient cohorts or those with eosinophilic gastrointestinal disorders are awaited in FD patients co-morbid with psychiatric conditions. As shown in the study by Wauters et al. (2021a), perceived stress was greater in FD patients compared to healthy controls, and was unaffected by PPI therapy. Further, *ex vivo* permeability in FD patients was higher with a significant decrease to levels of healthy controls following PPI therapy (Wauters et al., 2021a). Nonetheless, the PPI’s anti-inflammatory effects did not seem to be responsible for this effect (Wauters et al., 2021a). Thus, the role of duodenal eosinophils and mast cells as therapeutic targets in co-morbid FD sufferers requires further investigation.

#### 4.3.4. Acupuncture

As previously mentioned, dyspeptic symptoms are more likely to arise when multiple functional abnormalities are present (Vanheel et al., 2017). There may be multiple pathophysiological mechanisms responsible for symptoms, so agents targeting only one mechanism are unlikely to be effective (Lee, 2021).

In cases of refractory symptoms, acupuncture can be used as an alternative treatment to conventional therapies (Masuy et al., 2019). This treatment involves the use of solid metallic needles, which penetrate the skin and are then manually manipulated, such as lifting, twisting, and thrusting, in order to stimulate specific acupuncture points (Ko et al., 2016; Tomita et al., 2018).

Neuroimaging research has accumulated a growing body of evidence indicating significant functional and structural changes in multiple brain regions in FD sufferers, including the frontal cortex, somatosensory cortex, postcentral gyrus, precuneus, and caudate tail (Zeng et al., 2009; Nan et al., 2014; Lee et al., 2016; Qi et al., 2020). Interestingly, acupuncture can regulate, to a certain extent, some of the aforementioned abnormal functional activities (Zeng et al., 2012; Chen et al., 2021; Dong et al., 2022). Acupuncture and electroacupuncture have been studied extensively for their effects on gastric and intestinal motility impairments. However, more attention is now being focused on the effect of these techniques on the brain-gut interaction (Ye et al., 2018; Masuy et al., 2019).

Several systematic reviews and meta-analyses have confirmed acupuncture’s promising efficacy in treating FD patients (Kim et al., 2015; Pang et al., 2016; Zhou et al., 2016; Ho et al., 2017; Guo et al., 2020; Zhang J. et al., 2020). A Cochrane review, however, found no evidence of benefit (Lan et al., 2014). The inconsistent results of meta-analyses are likely to be attributed to the low levels of evidence (no explicit blinding or randomization and short follow-up periods), as well as the inherent characteristics of the acupuncture procedure (no single adequate sham intervention for acupuncture trials, impossibility of complete blinding, positive therapeutic effects produced by sham acupuncture, and operator dependence due to no standardized protocol) (Yoon et al., 2022). However, RCTs involving acupuncture therapy in FD research are typically subject to these limitations. In general, a number of RCTs have well documented acupuncture’s efficacy in patients with FD.

The physiological abnormalities known to directly cause FD symptoms are thought to be gastric motor and sensory dysfunction. There is evidence that acupuncture modulates gastric sensory-motor function, which accounts for its therapeutic effect on some FD patients (Tang et al., 2020; Zhang S. et al., 2020; Li Y. et al., 2022).

It is however possible that constant exposure to multiple factors (e.g., *H. pylori* infection, increased gastric acid, and altered microbiota) may lead to priming of the duodenum, as manifested by increased mucosal permeability and low-grade inflammation, thus modifying these sensory-motor abnormalities and ultimately resulting in the manifestation of symptoms (Miwa et al., 2019). Acupuncture’s alleviation of sensory-motor dysfunction may therefore be explained more explicitly in terms of its effects on the root cause, i.e., impaired intestinal permeability and duodenal micro-inflammation.

It is important to note, however, that although subtle duodenal inflammation characterized by increased eosinophils and mast cells with their degranulation has led to new insights into immune-mediated pathophysiology, there is limited evidence to support the effect of acupuncture on duodenal low-grade inflammation in FD, since most data come from studies that are not specifically targeted at this disease. The possible mechanism of action includes acupuncture’s immunomodulatory effects to maintain homeostasis under Th2-skewed conditions, inhibition of duodenal mast cell degranulation, and regulating inflammatory responses as well as modulating gastrointestinal function by stimulating distinct autonomic pathways through regional or heterotopic acupoints (Yang et al., 2022).

In the context of brain to gut interaction, abnormal brain activation or altered engagement of descending or ascending pathways may contribute to the pathophysiology. Greater levels of glycometabolism in the anterior cingulate cortex, insula, middle cingulate cortex, and cerebellum were found to have a positive correlation with Symptom Index of Dyspepsia (SID) scores, while having a negative correlation with SID scores (Zeng et al., 2011). As reported by Zeng et al. (2012), acupuncture treatment reduced glycometabolism in the brainstem, anterior cingulate cortex, insula, thalamus, and hypothalamus, resulting in a decrease in SID scores and an increase in NDI scores.

From the gut to brain perspective, intestinal microbiota plays a significant role in altering brain function by balancing host immune responses; immune responses are further considered to be a key regulator of the gut-brain axis. As evidence grows, altered microbiota may contribute to the pathophysiology and clinical course of FGIDs, rather than simply being an epiphenomenon. It appears, based on the IBS-related data (Song et al., 2020), that acupuncture may help by managing microbial dysbiosis, improving the interaction between the intestinal microbiota and the brain-gut axis, and decreasing pro-inflammatory cytokines. Thus, acupuncture may be able to suppress inflammation not only by inhibiting the release of inflammatory cytokines *via* the various somatic autonomic reflex pathways, as was discussed previously, but also by regulating the brain-gut axis through the intestinal microbiota. New approaches to FD can therefore be fostered by acupuncture’s ability to modulate gut microbiota, interrupting the gut-brain vicious cycle.

Several chemosensing receptors are found in the gastrointestinal mucosa that detect nutrients in the lumen (Farré and Tack, 2013; Depoortere, 2014). This avalanche-effect occurs as a result of gut peptides being released from enteroendocrine cells into the bloodstream due to the detection of nutrients in the duodenum and jejunum (Farré and Tack, 2013; Depoortere, 2014). These signals are either transmitted *via* the vagus nerve or directly by fenestrated endothelial cells within circumventricular organs, such as the area postrema (Farré and Tack, 2013; Depoortere, 2014). There has been recent evidence that intensities of symptoms in FD may be associated with nutritionally specific altered release of gut hormones.

The peptide ghrelin, which has been studied extensively in FD pathophysiology, was captured in the present study with a strong citation burst. Ghrelin is an appetite-stimulating hormone that is primarily produced by gastric X/A-like endocrine cells in rats and P/D1 type cells in humans (Cummings et al., 2001; Farré and Tack, 2013). Ghrelin is produced and released primarily by the stomach, but it is also produced and released in small amounts by the small intestine and the brain, where it regulates gastric motility and appetite *via* mammalian/mechanistic target of rapamycin (mTOR) signaling activation (Yagi et al., 2013). As a ligand for growth hormone secretagogue receptors, acyl ghrelin aids normal gastrointestinal motility by acting on the brain-gut axis (Yagi et al., 2013).

Since ghrelin and acyl ghrelin concentrations were found to be subnormal in FD patients, stimulating ghrelin receptors may be able to improve stomach emptying in these cohorts (Shindo et al., 2009; Kim et al., 2012). Electroacupuncture elevates ghrelin levels in the hypothalamus, stomach antrum, and small intestine and promotes adenosine 5′-monophosphate-activated protein kinase (AMPK)/Tuberous Sclerosis Complex 2 (TSC2)/Ras homolog enriched in brain (Rheb) signaling by inhibiting mTOR, resulting in dyspepsia relief (Tang et al., 2020).

There is evidence that ghrelin and acyl ghrelin alleviate depressive-like behavior in animal models of chronic stress (Fan et al., 2017; Huang et al., 2017) as well as suppress certain inflammatory responses (Ziko et al., 2018). Through ghrelin-AMPK signaling, electroacupuncture-induced ghrelin production improved gastrointestinal motility and suppressed stress-induced intestinal mucosal inflammation (Tang et al., 2020). Consequently, acupuncture may restore ghrelin expression in FD, which indicates a potentially dual therapeutic role for mood disorders and mucosal inflammatory cell infiltration, therefore improving gastrointestinal motility (Takeshita, 2020). It should be noted, however, that the enteroendocrine cells, epithelial cells, immune cells, autonomic nerve, and gut microflora are interdependent and operate in a convoluted circuit system by undefined mechanisms. In view of these difficulties in studying and comprehending such broad ranges of interactions, any comprehensive understanding of acupuncture in FD is presently imperceptible.

Overall, acupuncture has been shown to be an effective alternative treatment for patients suffering from FD. Mechanistically, investigations with patients and animal models have indicated that acupuncture may ameliorate intestinal inflammation, regulate the brain-gut axis *via* modulating brain processing of visceral pain, reforming intestinal microbiota, and restoring peptide hormones expression, and thereby alleviate FD symptoms.

## 5. Future perspectives

In this work, we performed a bibliometric analysis on 2,957 papers on FD that were obtained from the WOSCC of Clarivate Analytics between 2006 and 2022. The greatest level of publication activity was observed among Asian researchers from Japan, South Korea, and China. However, the extent of their worldwide collaboration fell short of extensiveness and diversification, and their research outcomes appeared to be less influential than those produced by their Western counterparts.

North America and Europe are obviously at the forefront of FD research, as shown by the current research. In recent bibliometric studies investigating the brain-gut axis and IBS, this conclusion was further corroborated (Zyoud et al., 2021; Wu et al., 2022). Besides the overwhelming support for research, the abundance of highly resourced research environments, and the higher availability of a well-trained workforce, the diversity of the lines of research developed by each research group certainly contributes to Western countries’ exceptional research performance. A closer look at the research directions of each prolific author in Table 2 can provide an indication of this. The betweenness centrality of the collaborative network demonstrated a dominance and influence of some researchers (e.g., Nicholas J. Talley, Jan Tack, Gerald Holtmann, Michael Camilleri, Ken Haruma, and Paul Moayyedi) over others in FD research. As likely initiators of collaborative relationships, these authors are typically the ones to provide the central funding or resources to support the community clusters in the network in which they are involved (Ekundayo and Okoh, 2020). Consequently, removal of such authors would result in fragmentation of the collaborative network and a general decline in FD research (Ekundayo and Okoh, 2020).

Jan Tack and colleagues, for example, are actively involved in the basic research pertaining to FD, which involves the investigation of duodenal pathology (i.e., increased permeability and infiltration of immune cells) and the interaction of such pathology with luminal (acid, bile acid, and microbiota) and central (stress) factors. In addition, their work is concerned with the evaluation of clinical research that may yield further proof of the efficacy of specific traditional herbal therapy and other medications for the benefit of FD patients. The overlap between FD and other FGIDs is also one of their primary research foci. Productive Asian authors, on the other hand, are more focused in providing clinical evidence of CAM as treatment strategies, such as herbal medicine and acupuncture. The evidence for these treatments, however, remains weak, and the trials were primarily published in less prestigious journals, making herbal medications less generalized on a global basis. Much of this challenge stems from difficulty in designing of the trials. Herbal remedies are typically prescribed using combinations of dried herbs decocted with water, with each combination having a different flavor. As a result, it is challenging to create a placebo that is identical to the studied drug, making it impossible to conduct a completely blinded study (Wan et al., 2013). In addition, research to date has been extremely heterogeneous in terms of the study population due to unclear diagnostic criteria, interventions in the form of varying dosages and combinations of herbal prescriptions, and treatment duration. A recent meta-analysis concluded that sham manual acupuncture and sham electroacupuncture significantly improved the symptoms and QOL scores of FD patients (Liu et al., 2022). Thus, clinical trials involving CAM may actually demonstrate a high placebo effect. In the context of CAM in FD patients, the main question regarding placebo has involved whether a particular therapy results in more symptom improvement than a placebo. However, clinical trials involving acupuncture and herbal compounds where a placebo control was not included further undermines their evidence base. Taken together, despite the widespread use of CAM, many of the studies to date are underpowered, had low-quality evidence, and have not been consistently replicated (Yoon et al., 2022). Among those studies, only a few were suitable for examining the effectiveness of herbal medications and acupuncture in FD patients. Nevertheless, this opportunity presents itself for clarifying these issues and contributing to this important area of research.

In addition, despite the growing body of evidence from clinical research on CAM in recent years, basic research has lagged behind. The potential mechanisms of acupuncture’s effect on FD, for example, have not yet been thoroughly explored, as previously mentioned. Many studies have linked the related mechanism of action with improved gastric motility and gastric hypersensitivity, both of which are directly related to the manifestation of dyspeptic symptoms; however, very few investigations have looked more deeply into the potential pathogenic features from the perspective of the primed duodenum, which is known to induce both gastric visceral hypersensitivity and abnormal motility (Wauters et al., 2020a). CAM research on FD, therefore, appears disconnected from these emerging targets.

Further, FD patients can also exhibit certain overlaps with GERD and IBS, and are, therefore, often misclassified (Quigley and Lacy, 2013; Pleyer et al., 2014; von Wulffen et al., 2019). Compared to patients with single FGID, those with overlap syndrome visit the hospital more frequently and report more frequent and severe gastrointestinal symptoms, more severe depression, as well as lower QOL (Lee et al., 2020; Barberio et al., 2022; Jones et al., 2022). FGID overlap syndrome is often explained by diseases sharing the same pathophysiology (such as visceral hypersensitivity and gastrointestinal motility disorder) occurring in multiple organs instead of being confined to one gastrointestinal segment. IBS is characterized by inflammatory infiltrates in the colon or duodenum, predominantly composed of lymphocytes and mast cells (Barbara et al., 2004; Walker et al., 2009). Infiltration and degranulation of eosinophils in the colonic mucosa are also likely to be associated with IBS development (Salvo-Romero et al., 2020; Casado-Bedmar et al., 2022). It is therefore possible for proximal small intestinal eosinophilia to cause FD rather than IBS. The development of IBS occurs most often in those with distal small intestinal or colonic immune activation, whereas extensive intestinal involvement may lead to both overlapping (Kaji et al., 2010; Talley, 2020). The delayed gastric emptying caused by duodenal immune activation and the resultant duodeno-gastric reflexes in FD may, in turn, lead to increased refluxates and distension of the stomach, which further promotes transient lower esophageal sphincter relaxations in post-prandial periods and contributes to the development of GERD (McCallum et al., 1981; Lee et al., 2004; Ronkainen et al., 2019). There is a substantial overlap between IBS and GERD with FD, suggesting that these disorders may share a common etiopathogenesis, namely duodenal eosinophilia, although the extent to which this contributes to their frequent overlap is unknown.

There is a plethora of evidence demonstrating the efficacy of herbal medicine, such as *Banxia-xiexin-tang* (also termed as *Banha-sasim-tang* in Traditional Korean medicine or *Hange-shashin-to* in Kampo medicine), Chaihu-shugan-san (Sihosogan-san in Traditional Korean medicine or *Saikosokan-to* in Kampo medicine), *Liu-jun-zi-tang* (termed as *Yukgunja-tang* in Traditional Korean medicine or *Rikkunshito* in Kampo medicine), and STW-5 (Iberogast) as well as acupuncture in treating FGIDs and GERD (Dai et al., 2020; Tan et al., 2020; Gwee et al., 2021; Yoon et al., 2022). In light of this, it remains to be determined whether these therapeutic strategies are optimally suited to overlapping patient cohorts. Additionally, basic studies investigating the mechanisms of CAM in treating overlap syndromes in FD patients may facilitate a greater understanding of potential therapeutic targets by sharpening the focus on the mucosal eosinophil-mast cell axis.

In conclusion, although 10% of the world’s population suffers with FD, the most common gastric sensorimotor disorder, it is underappreciated in clinical practice and tends to be refractory to therapy. Even though our understanding of the pathophysiology of FD has significantly advanced over the last two decades, realizing it goes beyond mere motility disorders and even psychosomatic syndromes, it is apparent that we are only at the beginning of unraveling this complex biology. As highlighted in this review, a research agenda for FD was proposed, focusing on the following identified research gaps: (1) there is mounting evidence that immune activation plays a role in FD, but its underlying causes are still a hot area of research. Additionally, there are still few therapeutic options available for targeting this immune activation in FD. From a global standpoint, intense collaboration between immunologists, epidemiologists, pathologists, neuroscientists, microbiologists, and psychologists is unquestionably the cornerstone of future research advancement. Only through a multidisciplinary scientific approach can FGIDs hold promise for fascinating discoveries and new targets for impacting clinical and therapeutical management of the disorder; (2) despite having the most publications in this topic, the Eastern countries, particularly China, Japan, and South Korea, showed minimal academic influence and little signs of global cooperative partnerships. In this regard, it is urgent for Asia to engage in collaborative efforts with other regions; (3) while there are robust research activities for CAM in Asian countries, there has been no consistent conclusion regarding its effectiveness. The basic research of CAM in FD has mostly concentrated on the dysregulated gut motility and gut sensation; however, there is a dearth of data on a potentially more significant factor—duodenal epithelium, that is, how the altered duodenal immune profiles and the gut flora are affected by CAM. Thus, well-planned, large-scale trials are required to assess the efficacy of CAM in treating FD, particularly in Asia. Basic research on CAM could benefit from the incorporation of novel viewpoints such as shifts in bile acid pool, duodenal immune alterations, mucosal barrier dysfunction, and miscommunication between the brain and the gut; (4) FD tended to overlap with another FGID (80% or more have an overlap) (Xiong et al., 2017). Current clinical guidelines for the treatment of FGID overlap syndrome frequently result in a multidrug regimen, which lowers patient adherence to therapy. In this context, CAM, which has recently been considered as an adjunctive treatment for FD, may be anticipated to have a role to play; (5) additional research that focuses on the identification of organic biomarkers or pathologic abnormalities to assist in the diagnosis of FD is required. Potentially game-changing emerging biomarkers that transform the diagnostic landscape include biopsy-detected duodenal inflammation, confocal laser endomicroscopy-identified cell gaps, and altered gut microbiota signatures. There may, however, be challenges to further exploring and confirming these novel pathophysiologic mechanisms. For instance, evaluating the intestinal microbiota signatures in FD is confounded by the wide heterogeneity of the human microbiota as well as the hostile and changing environment of the proximal small intestine, in which microbes have finicky growth requirements (Guarner and Malagelada, 2003; Turnbaugh et al., 2010). Furthermore, sampling and storage methods currently used for small bowel research are limited (Bharti and Grimm, 2021); (6) as to the exact mechanism of action of probiotics used in FD patients, it is unclear and is likely to vary from patient to patient. There is a need for further investigation to evaluate the treatment efficacy of probiotics in FD and to identify the appropriate strain and dose. In the meantime, as a growing body of evidence suggests that the intestinal microbiota interacts with neuroendocrine pathways in the brain-gut and gut-brain axes to potentially affect psychosocial symptoms as well as gastrointestinal discomfort in FD, probiotics appear to be a promising treatment option for co-morbid psychological distress and affective disorders, which warrants additional investigation; (7) in light of the recent observations, a greater emphasis has been placed in last few years on demonstrating that altered microbiomes lead to immune activation rather than an associative link. There is, however, a need for further research to determine the causal association between altered mucosal barrier dysfunction and gastrointestinal dysbiosis. Often, it is hypothesized that impaired barrier function enables uncontrolled entry of antigens into the lamina propria, resulting in an immune response. Hence, it remains an open question whether increased permeability plays a causal role in these conditions or if it is merely a consequence of immune activation although mechanistic insights into this last option are lacking in humans with FD. The unanswered and controversial issues seem to have opened up a new opportunity in the search for a more effective curative therapeutic plan tailored to a patient’s dyspeptic symptoms, whether it be by modifying diet, manipulating microbiota, stabilizing intestinal barriers or targeting inflammatory cytokines.

## 6. Strength and limitations

This study, like other bibliometric studies (Lin et al., 2021a; Luo and Lin, 2021; Zhang and Lin, 2022; Zhang L. et al., 2023), quantifies and draws qualitative conclusions from the size and characteristics of previously published academic publications from one of the most renowned and esteemed databases to examine the development of scientific research output in the field of FD and to identify potential future research directions and opportunities for collaboration. The utilization of a novel knowledge synthesis method and cutting-edge bibliometric analysis and mapping tools is the primary strength of the present study. By doing so, we were able to present an in-depth analysis of FD research and highlight several crucial aspects of the literature production process. In addition, the consistency of the results obtained from the two bibliometric mapping tools ensures that the information derived is highly valuable and reliable. On the level of meta-knowledge, we identified the FD research processes from a multi-dimensional perspective, such as the most productive countries and institutions, cooperation patterns among entities, and a timeline for knowledge development, thereby aiding researchers in identifying the most appropriate research partners and resources from the outset. In addition, we identified hot themes and trending topics.

This research study did have some limitations. First, despite being a reputable platform and one of the most thorough, accurate, and unbiased sources for literature searching, not all journals, institutions, or specific authors who contributed FD-related research are necessarily contained in the WOSCC database. Using alternative search engines may have yielded marginally different results. However, the database was selected due to its superior metadata, which permits multiple dimensions of analysis to be examined using network visualization software, as well as its ability to assign document type labels more precisely than other databases (Yeung, 2019). In addition, the qualitative nature of the thematic analysis increased the likelihood of subjective conclusions.

## 7. Conclusion

To the best of our knowledge, this is the first comprehensive and synthetic knowledge synthesis of FD research. The bibliometric analysis revealed a surge in interest in FD research in recent years, which may have been influenced by the introduction of the most recent Rome IV criteria, which underwent significant revisions, particularly in the definition of FD. The analysis of author keywords revealed that the field was most thoroughly studied with regard to *H. pylori* infection, pathophysiological mechanisms (such as duodenal inflammation; dysbiosis; and psychological factors), extraintestinal co-morbidities and overlap syndromes related to FD, herbal medicine, diabetic gastroparesis, and dietary factors in FD. The eosinophil-mast cell axis, gut microbiota, and psychological co-morbidity were among the potential central themes identified by the hot topic analysis in the pathophysiology of FD. More research is needed to determine the efficacy of anti-inflammatory drugs, mucosal protective agents, psychotherapy, dietary therapy, and microecological therapy in the treatment of FD.

This analysis can assist researchers and practitioners in comprehending the broader aspects of FD research and its emerging research trends. As a result, it can help a health professional gain perspective on the most significant research themes and act as a springboard for additional research.

## Author contributions

XT and FW conceived and designed the experiments. TZ, XM, and BZ conducted the experiments, analyzed and interpreted the data, and wrote the manuscript. TZ, BZ, and JZ revised this manuscript critically for significant intellectual content. XT gave final approval of the manuscript. All authors read and approved the final version of the manuscript.

## References

[B1] Abbasi A. Hossain L. Leydesdorff L. (2012). Betweenness centrality as a driver of preferential attachment in the evolution of research collaboration networks. *J. Informetr.* 6 403–412. 10.1016/j.joi.2012.01.002

[B2] Albert M. Rowland P. Friesen F. Laberge S. (2020). Interdisciplinarity in medical education research: Myth and reality. *Adv. Health Sci. Educ. Theory Pract.* 25 1243–1253. 10.1007/s10459-020-09977-8 32583329 PMC7704507

[B3] Alvarez-Peregrina C. Martinez-Perez C. Villa-Collar C. Sánchez-Tena M. (2021). A bibliometric and citation network analysis of myopia genetics. *Genes (Basel)* 12:447. 10.3390/genes12030447 33801043 PMC8003911

[B4] Ansari F. Pourjafar H. Tabrizi A. Homayouni A. (2020). The effects of probiotics and prebiotics on mental disorders: A review on depression, anxiety, alzheimer, and autism spectrum disorders. *Curr. Pharm. Biotechnol.* 21 555–565. 10.2174/1389201021666200107113812 31914909

[B5] Aria M. Cuccurullo C. (2017). bibliometrix: An R-tool for comprehensive science mapping analysis. *J. Informetr.* 11 959–975. 10.1016/j.joi.2017.08.007

[B6] Aro P. Talley N. Johansson S. Agréus L. Ronkainen J. (2015). Anxiety is linked to new-onset dyspepsia in the Swedish population: A 10-year follow-up study. *Gastroenterology* 148 928–937. 10.1053/j.gastro.2015.01.039 25644097

[B7] Aro P. Talley N. Ronkainen J. Storskrubb T. Vieth M. Johansson S. (2009). Anxiety is associated with uninvestigated and functional dyspepsia (Rome III criteria) in a Swedish population-based study. *Gastroenterology* 137 94–100. 10.1053/j.gastro.2009.03.039 19328797

[B8] Azer S. Azer S. (2018). What can we learn from top-cited articles in inflammatory bowel disease? A bibliometric analysis and assessment of the level of evidence. *BMJ Open* 8:e021233. 10.1136/bmjopen-2017-021233 30002009 PMC6082456

[B9] Aziz I. Palsson O. Törnblom H. Sperber A. Whitehead W. Simrén M. (2018). Epidemiology, clinical characteristics, and associations for symptom-based Rome IV functional dyspepsia in adults in the USA, Canada, and the UK: A cross-sectional population-based study. *Lancet Gastroenterol. Hepatol.* 3 252–262. 10.1016/S2468-1253(18)30003-7 29396034

[B10] Baier-Fuentes H. González-Serrano M. Alonso-Dos Santos M. Inzunza-Mendoza W. Pozo-Estrada V. (2020). Emotions and sport management: A bibliometric overview. *Front. Psychol.* 11:1512. 10.3389/fpsyg.2020.01512 32754088 PMC7366861

[B11] Banerjee S. Sindberg G. Wang F. Meng J. Sharma U. Zhang L. (2016). Opioid-induced gut microbial disruption and bile dysregulation leads to gut barrier compromise and sustained systemic inflammation. *Mucosal. Immunol.* 9 1418–1428. 10.1038/mi.2016.9 26906406 PMC4996771

[B12] Barbara G. Stanghellini V. De Giorgio R. Cremon C. Cottrell G. Santini D. (2004). Activated mast cells in proximity to colonic nerves correlate with abdominal pain in irritable bowel syndrome. *Gastroenterology* 126 693–702. 10.1053/j.gastro.2003.11.055 14988823

[B13] Barberio B. Yiannakou Y. Houghton L. Black C. Savarino E. Ford A. (2022). Overlap of Rome IV irritable bowel syndrome and functional dyspepsia and effect on natural history: A longitudinal follow-up study. *Clin. Gastroenterol. Hepatol.* 20 e89–e101. 10.1016/j.cgh.2021.04.011 33839276

[B14] Barden N. (2004). Implication of the hypothalamic-pituitary-adrenal axis in the physiopathology of depression. *J. Psychiatry Neurosci.* 29 185–193. 15173895 PMC400688

[B15] Barthel S. Annis D. Mosher D. Johansson M. (2006). Differential engagement of modules 1 and 4 of vascular cell adhesion molecule-1 (CD106) by integrins alpha4beta1 (CD49d/29) and alphaMbeta2 (CD11b/18) of eosinophils. *J. Biol. Chem.* 281 32175–32187. 10.1074/jbc.M600943200 16943205

[B16] Barthelemy M. (2004). Betweenness centrality in large complex networks. *Eur. phys. J. B* 38 163–168. 10.1140/epjb/e2004-00111-4

[B17] Beeckmans D. Farré R. Riethorst D. Keita Å Augustijns P. Söderholm J. (2020). Relationship between bile salts, bacterial translocation, and duodenal mucosal integrity in functional dyspepsia. *Neurogastroenterol. Motil.* 32:e13788. 10.1111/nmo.13788 31916349

[B18] Beeckmans D. Riethorst D. Augustijns P. Vanuytsel T. Farré R. Tack J. (2018). Altered duodenal bile salt concentration and receptor expression in functional dyspepsia. *United Eur. Gastroenterol. J.* 6 1347–1355. 10.1177/2050640618799120 30386607 PMC6206534

[B19] Begum M. Lewison G. Lawler M. Sullivan R. (2018). Mapping the European cancer research landscape: An evidence base for national and Pan-European research and funding. *Eur. J. Cancer* 100 75–84. 10.1016/j.ejca.2018.04.017 30014883

[B20] Bharti R. Grimm D. (2021). Current challenges and best-practice protocols for microbiome analysis. *Brief Bioinform.* 22 178–193. 10.1093/bib/bbz155 31848574 PMC7820839

[B21] Black C. Paine P. Agrawal A. Aziz I. Eugenicos M. Houghton L. (2022). British Society of Gastroenterology guidelines on the management of functional dyspepsia. *Gut* 71 1697–1723. 10.1136/gutjnl-2022-327737 35798375 PMC9380508

[B22] Blanchard C. Rothenberg M. (2009). Chemotactic factors associated with eosinophilic gastrointestinal diseases. *Immunol. Allergy Clin. North Am.* 29 141–8, xi. 10.1016/j.iac.2008.10.002 19141349 PMC4122321

[B23] Blanchard C. Mingler M. Vicario M. Abonia J. Wu Y. Lu T. (2007). IL-13 involvement in eosinophilic esophagitis: transcriptome analysis and reversibility with glucocorticoids. *J. Allergy Clin. Immunol.* 120 1292–1300. 10.1016/j.jaci.2007.10.024 18073124

[B24] Blažun Vošner H. Železnik D. Kokol P. Vošner J. Završnik J. (2017). Trends in nursing ethics research: Mapping the literature production. *Nurs. Ethics* 24 892–907. 10.1177/0969733016654314 27364534

[B25] Blažun H. Kokol P. Vošner J. (2015). Research literature production on nursing competences from 1981 till 2012: A bibliometric snapshot. *Nurse Educ. Today* 35 673–679. 10.1016/j.nedt.2015.01.002 25616510

[B26] Bochner B. (2000). Road signs guiding leukocytes along the inflammation superhighway. *J. Allergy Clin. Immunol.* 106 817–828. 10.1067/mai.2000.110813 11080701

[B27] Bonaz B. Bazin T. Pellissier S. (2018). The vagus nerve at the interface of the microbiota-gut-brain axis. *Front. Neurosci.* 12:49. 10.3389/fnins.2018.00049 29467611 PMC5808284

[B28] Bonaz B. Sinniger V. Pellissier S. (2016). Anti-inflammatory properties of the vagus nerve: potential therapeutic implications of vagus nerve stimulation. *J. Physiol.* 594 5781–5790. 10.1113/JP271539 27059884 PMC5063949

[B29] Brika S. Chergui K. Algamdi A. Musa A. Zouaghi R. E. (2022). Learning research trends in higher education in light of COVID-19: A bibliometric analysis. *Front. Psychol.* 12:762819. 10.3389/fpsyg.2021.762819 35308075 PMC8929398

[B30] Broadus R. N. (1987). Toward a definition of “bibliometrics”. *Scientometrics* 12 373–379. 10.1007/BF02016680

[B31] Brook R. Kleinman N. Choung R. Melkonian A. Smeeding J. Talley N. (2010). Functional dyspepsia impacts absenteeism and direct and indirect costs. *Clin. Gastroenterol. Hepatol.* 8 498–503. 10.1016/j.cgh.2010.03.003 20304102

[B32] Brown H. Esterházy D. (2021). Intestinal immune compartmentalization: implications of tissue specific determinants in health and disease. *Mucosal Immunol.* 14 1259–1270. 10.1038/s41385-021-00420-8 34211125

[B33] Burns G. Bruce J. Minahan K. Mathe A. Fairlie T. Cameron R. (2023). Type 2 and type 17 effector cells are increased in the duodenal mucosa but not peripheral blood of patients with functional dyspepsia. *Front. Immunol.* 13:1051632. 10.3389/fimmu.2022.1051632 36685573 PMC9852875

[B34] Burns G. Carroll G. Mathe A. Horvat J. Foster P. Walker M. (2019a). Evidence for local and systemic immune activation in functional dyspepsia and the irritable bowel syndrome: A systematic review. *Am. J. Gastroenterol.* 114 429–436. 10.1038/s41395-018-0377-0 30839392

[B35] Burns G. Pryor J. Holtmann G. Walker M. Talley N. Keely S. (2019b). Immune activation in functional gastrointestinal disorders. *Gastroenterol. Hepatol. (N. Y.)* 15 539–548. 31802978 PMC6883739

[B36] Casado-Bedmar M. de-Faria F. Biskou O. Lindqvist C. Ranasinghe P. Bednarska O. (2022). Elevated F-EDN correlates with mucosal eosinophil degranulation in patients with IBS-A possible association with microbiota? *J. Leukoc Biol.* 111 655–665. 10.1002/JLB.4A0521-228R 34151454

[B37] Ceulemans M. Jacobs I. Wauters L. Vanuytsel T. (2022). Immune activation in functional dyspepsia: Bystander becoming the suspect. *Front. Neurosci.* 16:831761. 10.3389/fnins.2022.831761 35557605 PMC9087267

[B38] Chen C. (2006a). CiteSpace II: Detecting and visualizing emerging trends and transient patterns in scientific literature. *J. Am. Soc. Inf. Sci. Technol.* 57 359–377. 10.1002/asi.20317

[B39] Chen C. (2016b). *CiteSpace: A practical guide for mapping scientific literature.* Hauppauge, NY: Nova Science Publishers, 41–44.

[B40] Chen X. Yang K. Xu Y. Li K. (2019). Top-100 highest-cited original articles in inflammatory bowel disease: A bibliometric analysis. *Medicine (Baltimore)* 98:e15718. 10.1097/MD.0000000000015718 31096525 PMC6531102

[B41] Chen Y. Lian B. Li P. Yao S. Hou Z. (2022a). Studies on irritable bowel syndrome associated with anxiety or depression in the last 20 years: A bibliometric analysis. *Front. Public Health* 10:947097. 10.3389/fpubh.2022.947097 36045729 PMC9421367

[B42] Chen Y. Lin M. Zhuang D. (2022b). Wastewater treatment and emerging contaminants: Bibliometric analysis. *Chemosphere* 297:133932. 10.1016/j.chemosphere.2022.133932 35149018

[B43] Chen Y. Zhao Y. Tan R. Zhang P. Long T. Shi Y. (2021). The influence of stomach back-shu and front-mu points on insular functional connectivity in functional dyspepsia rat models. *Evid. Based Complement. Alternat. Med.* 2021:2771094. 10.1155/2021/2771094 34621320 PMC8490795

[B44] Cheng E. Zhang X. Huo X. Yu C. Zhang Q. Wang D. (2013). Omeprazole blocks eotaxin-3 expression by oesophageal squamous cells from patients with eosinophilic oesophagitis and GORD. *Gut* 62 824–832. 10.1136/gutjnl-2012-302250 22580413 PMC3552049

[B45] Cheng E. Zhang X. Wilson K. Wang D. Park J. Huo X. (2016). JAK-STAT6 pathway inhibitors block eotaxin-3 secretion by epithelial cells and fibroblasts from esophageal eosinophilia patients: Promising agents to improve inflammation and prevent fibrosis in EoE. *PLoS One* 11:e0157376. 10.1371/journal.pone.0157376 27310888 PMC4911010

[B46] Chong Y. Han C. Li J. Long X. (2020). Mapping global research trends in stem cell therapy for inflammatory bowel disease: A bibliometric analysis from 1991 to 2019. *J. Int. Med. Res.* 48:300060520965824. 10.1177/0300060520965824 33115290 PMC7607292

[B47] Chuah K. Wong M. Tan P. Lim S. Beh K. Chong S. (2022). Small intestinal bacterial overgrowth in various functional gastrointestinal disorders: A case-control study. *Dig. Dis. Sci.* 67 3881–3889. 10.1007/s10620-021-07227-4 34417923

[B48] Cirillo C. Bessissow T. Desmet A. Vanheel H. Tack J. Vanden Berghe P. (2015). Evidence for neuronal and structural changes in submucous ganglia of patients with functional dyspepsia. *Am. J. Gastroenterol.* 110 1205–1215. 10.1038/ajg.2015.158 26077177

[B49] Cobo M. J. López-Herrera A. G. Herrera-Viedma E. Herrera F. (2011). An approach for detecting, quantifying, and visualizing the evolution of a research field: A practical application to the Fuzzy Sets Theory field. *J. Informetr.* 5 146–166. 10.1016/j.joi.2010.10.002

[B50] Collen M. Loebenberg M. (1989). Basal gastric acid secretion in nonulcer dyspepsia with or without duodenitis. *Dig. Dis. Sci.* 34 246–250. 10.1007/BF01536059 2914546

[B51] Connelly T. Devane L. Kelly J. Wrafter P. Messaris E. (2016). The 100 classic papers in ulcerative colitis: A bibliometric analysis. *Expert Rev. Gastroenterol. Hepatol.* 10 1187–1195. 10.1080/17474124.2016.1216786 27531253

[B52] Cryan J. Dinan T. (2012). Mind-altering microorganisms: The impact of the gut microbiota on brain and behaviour. *Nat. Rev. Neurosci.* 13 701–712. 10.1038/nrn3346 22968153

[B53] Cummings D. Purnell J. Frayo R. Schmidova K. Wisse B. Weigle D. S. (2001). A preprandial rise in plasma ghrelin levels suggests a role in meal initiation in humans. *Diabetes* 50 1714–1719. 10.2337/diabetes.50.8.1714 11473029

[B54] Cunill O. M. Salva A. S. Gonzalez L. O. Mulet-Forteza C. (2019). Thirty-fifth anniversary of the International Journal of Hospitality Management: A bibliometric overview. *Int. J. Hosp. Manage.* 78 89–101. 10.1016/j.ijhm.2018.10.013

[B55] Dai Y. Wu Y. Wen H. Li R. Chen W. Tang C. (2020). Different traditional herbal medicines for the treatment of gastroesophageal reflux disease in adults. *Front. Pharmacol.* 11:884. 10.3389/fphar.2020.00884 32765255 PMC7378538

[B56] Dalile B. Van Oudenhove L. Vervliet B. Verbeke K. (2019). The role of short-chain fatty acids in microbiota-gut-brain communication. *Nat. Rev. Gastroenterol. Hepatol.* 16 461–478. 10.1038/s41575-019-0157-3 31123355

[B57] Dellon E. Peterson K. Murray J. Falk G. Gonsalves N. Chehade M. (2020). Anti-siglec-8 antibody for eosinophilic gastritis and duodenitis. *N. Engl. J. Med.* 383 1624–1634. 10.1056/NEJMoa2012047 33085861 PMC7600443

[B58] Demir E. Comba A. (2020). The evolution of celiac disease publications: A holistic approach with bibliometric analysis. *Ir. J. Med. Sci.* 189 267–276. 10.1007/s11845-019-02080-x 31422546

[B59] Depoortere I. (2014). Taste receptors of the gut: Emerging roles in health and disease. *Gut* 63 179–190. 10.1136/gutjnl-2013-305112 24131638

[B60] Dong S. Wang L. Gou Y. Ying H. Shen X. Meng Z. (2017). Risk factors of liver metastasis from advanced pancreatic adenocarcinoma: A large multicenter cohort study. *World J. Surg. Oncol.* 15:120. 10.1186/s12957-017-1175-7 28673297 PMC5496221

[B61] Dong X. Yin T. Yu S. He Z. Chen Y. Ma P. (2022). Neural responses of acupuncture for treating functional dyspepsia: An fMRI study. *Front. Neurosci.* 16:819310. 10.3389/fnins.2022.819310 35585920 PMC9108289

[B62] Drossman D. (2016). Functional gastrointestinal disorders: History, pathophysiology, clinical features and Rome IV. *Gastroenterology* 150, 1262–1279. 10.1053/j.gastro.2016.02.032 27144617

[B63] Drossman D. Hasler W. (2016). Rome IV-functional GI disorders: Disorders of gut-brain interaction. *Gastroenterology* 150 1257–1261. 10.1053/j.gastro.2016.03.035 27147121

[B64] Du L. Chen B. Kim J. Chen X. Dai N. (2018). Micro-inflammation in functional dyspepsia: A systematic review and meta-analysis. *Neurogastroenterol. Motil.* 30:e13304. 10.1111/nmo.13304 29392796

[B65] Duncanson K. Burns G. Pryor J. Keely S. Talley N. (2021). Mechanisms of food-induced symptom induction and dietary management in functional dyspepsia. *Nutrients* 13:1109. 10.3390/nu13041109 33800668 PMC8066021

[B66] Ekundayo T. Okoh A. (2020). Systematic assessment of mycobacterium avium subspecies paratuberculosis infections from 1911-2019: A growth analysis of association with human autoimmune diseases. *Microorganisms* 8:1212. 10.3390/microorganisms8081212 32784941 PMC7465227

[B67] Enck P. Azpiroz F. Boeckxstaens G. Elsenbruch S. Feinle-Bisset C. Holtmann G. (2017). Functional dyspepsia. *Nat. Rev. Dis. Primers* 3:17081. 10.1038/nrdp.2017.81 29099093

[B68] Fan J. Li B. Wang X. Zhong L. Cui R. (2017). Ghrelin produces antidepressant-like effect in the estrogen deficient mice. *Oncotarget* 8 58964–58973. 10.18632/oncotarget.19768 28938610 PMC5601706

[B69] Farré R. Tack J. (2013). Food and symptom generation in functional gastrointestinal disorders: physiological aspects. *Am. J. Gastroenterol.* 108 698–706. 10.1038/ajg.2013.24 23458851

[B70] Fleming M. Ehsan L. Moore S. Levin D. (2020). The enteric nervous system and its emerging role as a therapeutic target. *Gastroenterol. Res. Pract.* 2020:8024171. 10.1155/2020/8024171 32963521 PMC7495222

[B71] Ford A. Moayyedi P. Black C. Yuan Y. Veettil S. Mahadeva S. (2021). Systematic review and network meta-analysis: Efficacy of drugs for functional dyspepsia. *Aliment. Pharmacol. Ther.* 53 8–21. 10.1111/apt.16072 32936964

[B72] Fotopoulou C. Khan T. Bracinik J. Glasbey J. Abu-Rustum N. Chiva L. (2022). Outcomes of gynecologic cancer surgery during the COVID-19 pandemic: an international, multicenter, prospective CovidSurg-Gynecologic Oncology Cancer study. *Am. J. Obstet. Gynecol.* 227 735.e1–735.e25. 10.1016/j.ajog.2022.06.052 35779589 PMC9242690

[B73] Freedberg D. Kim L. Yang Y. (2017). The risks and benefits of long-term use of proton pump inhibitors: Expert review and best practice advice from the american gastroenterological association. *Gastroenterology* 152 706–715. 10.1053/j.gastro.2017.01.031 28257716

[B74] Freeman L. C. (1977). A set of measures of centrality based on betweenness. *Sociometry* 40 35–41. 10.2307/3033543

[B75] Friesen C. Andre L. Garola R. Hodge C. Roberts C. (2002). Activated duodenal mucosal eosinophils in children with dyspepsia: A pilot transmission electron microscopic study. *J. Pediatr. Gastroenterol. Nutr.* 35 329–333. 10.1097/00005176-200209000-00017 12352522

[B76] Friesen C. Sandridge L. Andre L. Roberts C. Abdel-Rahman S. (2006). Mucosal eosinophilia and response to H1/H2 antagonist and cromolyn therapy in pediatric dyspepsia. *Clin. Pediatr. (Phila)* 45 143–147. 10.1177/000992280604500205 16528434

[B77] Fukui A. Takagi T. Naito Y. Inoue R. Kashiwagi S. Mizushima K. (2020). Higher levels of streptococcus in upper gastrointestinal mucosa associated with symptoms in patients with functional dyspepsia. *Digestion* 101 38–45. 10.1159/000504090 31752012

[B78] Futagami S. Itoh T. Sakamoto C. (2015). Systematic review with meta-analysis: Post-infectious functional dyspepsia. *Aliment. Pharmacol. Ther.* 41 177–188. 10.1111/apt.13006 25348873

[B79] Futagami S. Shindo T. Kawagoe T. Horie A. Shimpuku M. Gudis K. (2010). Migration of eosinophils and CCR2-/CD68-double positive cells into the duodenal mucosa of patients with postinfectious functional dyspepsia. *Am. J. Gastroenterol.* 105 1835–1842. 10.1038/ajg.2010.151 20461070

[B80] Garfield E. Sher I. H. (1993). Key words plus [TM]-algorithmic derivative indexing. *J. Am. Soc. Inf. Sci.* 44 298–298. 10.1002/(SICI)1097-4571(199306)44:5<298::AID-ASI5>3.0.CO;2-A

[B81] Gargala G. Lecleire S. François A. Jacquot S. Déchelotte P. Ballet J. (2007). Duodenal intraepithelial T lymphocytes in patients with functional dyspepsia. *World J. Gastroenterol.* 13 2333–2338. 10.3748/wjg.v13.i16.2333 17511033 PMC4147143

[B82] Ghoshal U. Ghoshal U. (2017). Small intestinal bacterial overgrowth and other intestinal disorders. *Gastroenterol. Clin. North Am.* 46 103–120. 10.1016/j.gtc.2016.09.008 28164845

[B83] Grandinetti T. Biedermann L. Bussmann C. Straumann A. Hruz P. (2019). Eosinophilic gastroenteritis: Clinical manifestation, natural course, and evaluation of treatment with corticosteroids and vedolizumab. *Dig. Dis. Sci.* 64 2231–2241. 10.1007/s10620-019-05617-3 30982212

[B84] Griseri T. Arnold I. Pearson C. Krausgruber T. Schiering C. Franchini F. (2015). Granulocyte macrophage colony-stimulating factor-activated eosinophils promote interleukin-23 driven chronic colitis. *Immunity* 43 187–199. 10.1016/j.immuni.2015.07.008 26200014 PMC4518500

[B85] Guarner F. Malagelada J. (2003). Gut flora in health and disease. *Lancet* 361 512–519. 10.1016/S0140-6736(03)12489-0 12583961

[B86] Guler A. Waaijer C. Palmblad M. (2016). Scientific workflows for bibliometrics. *Scientometrics* 107 385–398. 10.1007/s11192-016-1885-6 27122644 PMC4833826

[B87] Guo Y. Wei W. Chen J. (2020). Effects and mechanisms of acupuncture and electroacupuncture for functional dyspepsia: A systematic review. *World J. Gastroenterol.* 26 2440–2457. 10.3748/wjg.v26.i19.2440 32476804 PMC7243644

[B88] Gurusamy S. Shah A. Talley N. Koloski N. Jones M. Walker M. (2021). Small intestinal bacterial overgrowth in functional dyspepsia: A systematic review and meta-analysis. *Am. J. Gastroenterol.* 116 935–942. 10.14309/ajg.0000000000001197 33734110

[B89] Gwee K. Holtmann G. Tack J. Suzuki H. Liu J. Xiao Y. (2021). Herbal medicines in functional dyspepsia-Untapped opportunities not without risks. *Neurogastroenterol. Motil.* 33:e14044. 10.1111/nmo.14044 33258198 PMC7900952

[B90] Hannibal K. Bishop M. (2014). Chronic stress, cortisol dysfunction, and pain: a psychoneuroendocrine rationale for stress management in pain rehabilitation. *Phys. Ther.* 94 1816–1825. 10.2522/ptj.20130597 25035267 PMC4263906

[B91] Hartono J. Mahadeva S. Goh K. (2012). Anxiety and depression in various functional gastrointestinal disorders: Do differences exist? *J. Dig. Dis.* 13 252–257. 10.1111/j.1751-2980.2012.00581.x 22500787

[B92] Hawkins D. T. (2001). Bibliometrics of electronic journals in information science. *Inf. Res.* 7:120.

[B93] He Z. Lin M. Xu Z. Yao Z. Chen H. Alhudhaif A. (2022). Deconv-transformer (DecT): A histopathological image classification model for breast cancer based on color deconvolution and transformer architecture. *Inf. Sci.* 608 1093–1112. 10.1016/j.ins.2022.06.091

[B94] Hirano I. Dellon E. Hamilton J. Collins M. Peterson K. Chehade M. (2020). Efficacy of dupilumab in a phase 2 randomized trial of adults with active eosinophilic esophagitis. *Gastroenterology* 158 111–122.e10. 10.1053/j.gastro.2019.09.042 31593702

[B95] Ho R. Chung V. Wong C. Wu J. Wong S. Wu I. (2017). Acupuncture and related therapies used as add-on or alternative to prokinetics for functional dyspepsia: Overview of systematic reviews and network meta-analysis. *Sci. Rep.* 7:10320. 10.1038/s41598-017-09856-0 28871092 PMC5583250

[B96] Huang H. Zhu X. Han Q. Wang Y. Yue N. Wang J. (2017). Ghrelin alleviates anxiety- and depression-like behaviors induced by chronic unpredictable mild stress in rodents. *Behav. Brain Res.* 326 33–43. 10.1016/j.bbr.2017.02.040 28245976

[B97] Hurst N. Kendig D. Murthy K. Grider J. (2014). The short chain fatty acids, butyrate and propionate, have differential effects on the motility of the guinea pig colon. *Neurogastroenterol. Motil.* 26 1586–1596. 10.1111/nmo.12425 25223619 PMC4438679

[B98] Igarashi M. Nakae H. Matsuoka T. Takahashi S. Hisada T. Tomita J. (2017). Alteration in the gastric microbiota and its restoration by probiotics in patients with functional dyspepsia. *BMJ Open Gastroenterol.* 4:e000144. 10.1136/bmjgast-2017-000144 28761692 PMC5508964

[B99] Järbrink-Sehgal M. Sparkman J. Damron A. Walker M. Green L. Rosen D. (2021). Functional dyspepsia and duodenal eosinophil count and degranulation: A multiethnic US veteran cohort study. *Dig. Dis. Sci.* 66 3482–3489. 10.1007/s10620-020-06689-2 33185786

[B100] Jia K. Wang P. Li Y. Chen Z. Jiang X. Lin C. (2022). Research landscape of artificial intelligence and e-learning: A bibliometric research. *Front. Psychol.* 13:795039. 10.3389/fpsyg.2022.795039 35250730 PMC8889112

[B101] Jones M. Shah A. Walker M. Koloski N. Holtmann G. Talley N. (2022). Overlap of heartburn, functional dyspepsia, and irritable bowel syndrome in a population sample: Prevalence, temporal stability, and associated comorbidities. *Neurogastroenterol. Motil.* 34:e14349. 10.1111/nmo.14349 35293084

[B102] Jones M. Tack J. Van Oudenhove L. Walker M. Holtmann G. Koloski N. (2017). Mood and anxiety disorders precede development of functional gastrointestinal disorders in patients but not in the population. *Clin. Gastroenterol. Hepatol.* 15 1014–1020.e4. 10.1016/j.cgh.2016.12.032 28087404

[B103] Kaji M. Fujiwara Y. Shiba M. Kohata Y. Yamagami H. Tanigawa T. (2010). Prevalence of overlaps between GERD, FD and IBS and impact on health-related quality of life. *J. Gastroenterol. Hepatol.* 25 1151–1156. 10.1111/j.1440-1746.2010.06249.x 20594232

[B104] Kakiyama G. Pandak W. Gillevet P. Hylemon P. Heuman D. Daita K. (2013). Modulation of the fecal bile acid profile by gut microbiota in cirrhosis. *J. Hepatol.* 58 949–955. 10.1016/j.jhep.2013.01.003 23333527 PMC3936319

[B105] Keely S. Talley N. (2020). Duodenal bile acids as determinants of intestinal mucosal homeostasis and disease. *Neurogastroenterol. Motil.* 32:e13854. 10.1111/nmo.13854 32323477

[B106] Keely S. Walker M. Marks E. Talley N. (2015). Immune dysregulation in the functional gastrointestinal disorders. *Eur. J. Clin. Invest.* 45 1350–1359. 10.1111/eci.12548 26444549

[B107] Khoruts A. Sadowsky M. (2016). Understanding the mechanisms of faecal microbiota transplantation. *Nat. Rev. Gastroenterol. Hepatol.* 13 508–516. 10.1038/nrgastro.2016.98 27329806 PMC5909819

[B108] Kim K. Chung S. Cho S. (2015). Efficacy of acupuncture treatment for functional dyspepsia: A systematic review and meta-analysis. *Complement. Ther. Med.* 23 759–766. 10.1016/j.ctim.2015.07.007 26645513

[B109] Kim Y. Lee J. Lee T. Cho J. Kim J. Kim W. (2012). Plasma levels of acylated ghrelin in patients with functional dyspepsia. *World J. Gastroenterol.* 18 2231–2237. 10.3748/wjg.v18.i18.2231 22611317 PMC3351774

[B110] Kindt S. Van Oudenhove L. Broekaert D. Kasran A. Ceuppens J. Bossuyt X. (2009). Immune dysfunction in patients with functional gastrointestinal disorders. *Neurogastroenterol. Motil.* 21 389–398. 10.1111/j.1365-2982.2008.01220.x 19126184

[B111] Klaassen T. Vork L. Smeets F. Troost F. Kruimel J. Leue C. (2022). The interplay between stress and fullness in patients with functional dyspepsia and healthy controls: An exploratory experience sampling method study. *Psychosom. Med.* 84 306–312. 10.1097/PSY.0000000000001012 34524263

[B112] Kleinberg J. (2002). “Bursty and hierarchical structure in streams,” in *Proceedings of the eighth ACM SIGKDD international conference on Knowledge discovery and data mining*, New York, NY, 91–101. 10.1145/775047.775061

[B113] Ko S. Kuo B. Kim S. Lee H. Kim J. Han G. (2016). Individualized acupuncture for symptom relief in functional dyspepsia: A randomized controlled trial. *J. Altern. Complement. Med.* 22 997–1006. 10.1089/acm.2016.0208 27732083

[B114] Kokol P. Blažun Vošner H. Završnik J. (2021a). Application of bibliometrics in medicine: A historical bibliometrics analysis. *Health Inf. Libr. J.* 38 125–138. 10.1111/hir.12295 31995273

[B115] Kokol P. Kokol M. Zagoranski S. (2022). Machine learning on small size samples: A synthetic knowledge synthesis. *Sci. Prog.* 105:368504211029777. 10.1177/00368504211029777 35220816 PMC10358596

[B116] Kokol P. Završnik J. Vošner H. B. (2018). Bibliographic-based identification of hot future research topics: An opportunity for hospital librarianship. *J. Hosp. Librariansh.* 18 315–322. 10.1080/15323269.2018.1509193

[B117] Kokol P. Završnik J. Turèin M. Vošner H. B. (2021b). Enhancing the role of academic librarians in conducting scoping reviews. *arXiv* [Preprint]. arXiv:2103.01099.

[B118] Koloski N. Jones M. Talley N. (2016). Evidence that independent gut-to-brain and brain-to-gut pathways operate in the irritable bowel syndrome and functional dyspepsia: A 1-year population-based prospective study. *Aliment. Pharmacol. Ther.* 44 592–600. 10.1111/apt.13738 27444264

[B119] Komori K. Ihara E. Minoda Y. Ogino H. Sasaki T. Fujiwara M. (2019). The altered mucosal barrier function in the duodenum plays a role in the pathogenesis of functional dyspepsia. *Dig. Dis. Sci.* 64 3228–3239. 10.1007/s10620-019-5470-8 30673985

[B120] Kourikou A. Karamanolis G. Dimitriadis G. Triantafyllou K. (2015). Gene polymorphisms associated with functional dyspepsia. *World J. Gastroenterol.* 21 7672–7682. 10.3748/wjg.v21.i25.7672 26167069 PMC4491956

[B121] Lacy B. Weiser K. Kennedy A. Crowell M. Talley N. (2013). Functional dyspepsia: The economic impact to patients. *Aliment. Pharmacol. Ther.* 38 170–177. 10.1111/apt.12355 23725230

[B122] Lahner E. Bellentani S. Bastiani R. Tosetti C. Cicala M. Esposito G. (2013). A survey of pharmacological and nonpharmacological treatment of functional gastrointestinal disorders. *United Eur. Gastroenterol. J.* 1 385–393. 10.1177/2050640613499567 24917987 PMC4040767

[B123] Lan L. Zeng F. Liu G. Ying L. Wu X. Liu M. (2014). Acupuncture for functional dyspepsia. *Cochrane Database Syst. Rev.* 1:CD008487. 10.1002/14651858.CD008487.pub2 25306866 PMC10558101

[B124] Larose M. Archambault A. Provost V. Laviolette M. Flamand N. (2017). Regulation of eosinophil and group 2 innate lymphoid cell trafficking in asthma. *Front. Med. (Lausanne).* 4:136. 10.3389/fmed.2017.00136 28848734 PMC5554517

[B125] Lawal I. A. Klink M. Ndungu P. Moodley B. (2019). Brief bibliometric analysis of “ionic liquid” applications and its review as a substitute for common adsorbent modifier for the adsorption of organic pollutants. *Environ. Res.* 175 34–51. 10.1016/j.envres.2019.05.005 31102948

[B126] Lee I. Wang H. Chae Y. Preissl H. Enck P. (2016). Functional neuroimaging studies in functional dyspepsia patients: A systematic review. *Neurogastroenterol. Motil.* 28 793–805. 10.1111/nmo.12793 26940430

[B127] Lee K. (2021). The usefulness of symptom-based subtypes of functional dyspepsia for predicting underlying pathophysiologic mechanisms and choosing appropriate therapeutic agents. *J. Neurogastroenterol. Motil.* 27 326–336. 10.5056/jnm21042 34210898 PMC8266502

[B128] Lee K. Demarchi B. Demedts I. Sifrim D. Raeymaekers P. Tack J. (2004). A pilot study on duodenal acid exposure and its relationship to symptoms in functional dyspepsia with prominent nausea. *Am. J. Gastroenterol.* 99 1765–1773. 10.1111/j.1572-0241.2004.30822.x 15330916

[B129] Lee M. Jung H. Lee K. Mun Y. Park S. (2019). Degranulated eosinophils contain more fine nerve fibers in the duodenal mucosa of patients with functional dyspepsia. *J. Neurogastroenterol. Motil.* 25 212–221. 10.5056/jnm18176 30827070 PMC6474707

[B130] Lee S. Ryu H. Choi S. Jang S. (2020). Psychological factors influence the overlap syndrome in functional gastrointestinal disorder and quality of life among psychiatric patients in South Korea. *Psychiatry Investig.* 17 262–267. 10.30773/pi.2019.0278 32151127 PMC7113182

[B131] Lewis K. Lutgendorff F. Phan V. Söderholm J. Sherman P. McKay D. (2010). Enhanced translocation of bacteria across metabolically stressed epithelia is reduced by butyrate. *Inflamm. Bowel Dis.* 16 1138–1148. 10.1002/ibd.21177 20024905

[B132] Li K. Feng C. Chen H. Feng Y. Li J. (2022). Trends in worldwide research in inflammatory bowel disease over the period 2012-2021: A bibliometric study. *Front. Med. (Lausanne).* 9:880553. 10.3389/fmed.2022.880553 35665364 PMC9160461

[B133] Li X. Chen H. Lu H. Li W. Chen X. Peng Y. (2010). The study on the role of inflammatory cells and mediators in post-infectious functional dyspepsia. *Scand. J. Gastroenterol.* 45 573–581. 10.3109/00365521003632576 20163288

[B134] Li Y. Nie Y. Sha W. Su H. (2002). The link between psychosocial factors and functional dyspepsia: An epidemiological study. *Chin. Med. J. (Engl)* 115 1082–1084. 12173597

[B135] Li Y. Yang N. Huang J. Lin L. Qi L. Ma S. (2022). Effects of electroacupuncture at different acupoints on functional dyspepsia rats. *Evid. Based Complement. Alternat. Med.* 2022 6548623. 10.1155/2022/6548623 35154349 PMC8824746

[B136] Liebregts T. Adam B. Bredack C. Gururatsakul M. Pilkington K. Brierley S. (2011). Small bowel homing T cells are associated with symptoms and delayed gastric emptying in functional dyspepsia. *Am. J. Gastroenterol.* 106 1089–1098. 10.1038/ajg.2010.512 21245834

[B137] Lin M. Chen Y. Chen R. (2021a). Bibliometric analysis on Pythagorean fuzzy sets during 2013–2020. *Int. J. Intell. Comput. Cybern.* 14 104–121. 10.1108/IJICC-06-2020-0067

[B138] Lin M. Huang C. Chen R. Fujita H. Wang X. (2021b). Directional correlation coefficient measures for Pythagorean fuzzy sets: Their applications to medical diagnosis and cluster analysis. *Complex Intell. Syst.* 7 1025–1043. 10.1007/s40747-020-00261-1

[B139] Liu J. Song G. Huang Y. Lv C. Wang Y. Wu D. (2022). Placebo response rates in acupuncture therapy trials for functional dyspepsia: A systematic review and meta-analysis. *J. Clin. Gastroenterol.* 56 299–310. 10.1097/MCG.0000000000001679 35180148 PMC8900996

[B140] Liu R. Walsh R. Sheehan A. (2019). Prebiotics and probiotics for depression and anxiety: A systematic review and meta-analysis of controlled clinical trials. *Neurosci. Biobehav. Rev.* 102 13–23. 10.1016/j.neubiorev.2019.03.023 31004628 PMC6584030

[B141] Lu F. Tu Y. Peng X. Wang L. Wang H. Sun Z. (2008). A prospective multicentre study of mycophenolate mofetil combined with prednisolone as induction therapy in 213 patients with active lupus nephritis. *Lupus* 17 622–629. 10.1177/0961203308089428 18625634

[B142] Luo C. Wen Z. Zhen Y. Wang Z. Mu J. Zhu M. (2018). Chinese research into severe ulcerative colitis has increased in quantity and complexity. *World J. Clin. Cases* 6 35–43. 10.12998/wjcc.v6.i3.35 29564356 PMC5852397

[B143] Luo Y. Lin M. (2021). Flash translation layer: A review and bibliometric analysis. *Int. J. Intell. Comput. Cybern.* 14 480–508. 10.1108/IJICC-02-2021-0034

[B144] Luo Y. Lin M. Pan Y. Xu Z. (2022). Dual locality-based flash translation layer for NAND flash-based consumer electronics. *IEEE Trans. Consum. Electron.* 68 281–290. 10.1109/TCE.2022.3189761

[B145] Mahadeva S. Goh K. (2006). Epidemiology of functional dyspepsia: A global perspective. *World J. Gastroenterol.* 12 2661–2666. 10.3748/wjg.v12.i17.2661 16718749 PMC4130971

[B146] Mallidou A. (2014). Mapping the landscape of knowledge synthesis. *Nurs. Manag. (Harrow)* 21 30–39. 10.7748/nm.21.5.30.e1242 25167127

[B147] Mao X. Guo S. Ni W. Zhang T. Liu Q. Du S. (2020). Electroacupuncture for the treatment of functional dyspepsia: A systematic review and meta-analysis. *Medicine (Baltimore)* 99:e23014. 10.1097/MD.0000000000023014 33157947 PMC7647594

[B148] Markowiak-Kopeć P. Śliżewska K. (2020). The effect of probiotics on the production of short-chain fatty acids by human intestinal microbiome. *Nutrients* 12:1107. 10.3390/nu12041107 32316181 PMC7230973

[B149] Marshall K. Tomasini A. Makky K. Kumar S. Mayer A. N. (2010). Dynamic Lkb1-TORC1 signaling as a possible mechanism for regulating the endoderm-intestine transition. *Dev. Dyn.* 239 3000–3012. 10.1002/dvdy.22437 20925120 PMC4420030

[B150] Masuy I. Van Oudenhove L. Tack J. (2019). Review article: Treatment options for functional dyspepsia. *Aliment. Pharmacol. Ther.* 49 1134–1172. 10.1111/apt.15191 30924176

[B151] Matsueda K. Hongo M. Tack J. Saito Y. Kato H. (2012). A placebo-controlled trial of acotiamide for meal-related symptoms of functional dyspepsia. *Gut* 61 821–828. 10.1136/gutjnl-2011-301454 22157329 PMC3345932

[B152] McCallum R. Berkowitz D. Lerner E. (1981). Gastric emptying in patients with gastroesophageal reflux. *Gastroenterology* 80 285–291. 10.1016/0016-5085(81)90716-2 7450419

[B153] Min J. Ocampo C. Stevens W. Price C. Thompson C. Homma T. (2017). Proton pump inhibitors decrease eotaxin-3/CCL26 expression in patients with chronic rhinosinusitis with nasal polyps: Possible role of the nongastric H,K-ATPase. *J. Allergy Clin. Immunol.* 139 130–141.e11. 10.1016/j.jaci.2016.07.020 27717558 PMC5222859

[B154] Miwa H. Nagahara A. Asakawa A. Arai M. Oshima T. Kasugai K. (2022). Evidence-based clinical practice guidelines for functional dyspepsia 2021. *J. Gastroenterol.* 57 47–61. 10.1007/s00535-021-01843-7 35061057 PMC8831363

[B155] Miwa H. Oshima T. Tomita T. Fukui H. Kondo T. Yamasaki T. (2019). Recent understanding of the pathophysiology of functional dyspepsia: Role of the duodenum as the pathogenic center. *J. Gastroenterol.* 54 305–311. 10.1007/s00535-019-01550-4 30767076 PMC6437122

[B156] Moayyedi P. Lacy B. Andrews C. Enns R. Howden C. Vakil N. (2017). ACG and CAG clinical guideline: Management of dyspepsia. *Am. J. Gastroenterol.* 112 988–1013. 10.1038/ajg.2017.154 28631728

[B157] Molina-Torres G. Rodriguez-Arrastia M. Roman P. Sanchez-Labraca N. Cardona D. (2019). Stress and the gut microbiota-brain axis. *Behav. Pharmacol.* 30 187–200. 10.1097/FBP.0000000000000478 30844962

[B158] Mukewar S. Sharma A. Rubio-Tapia A. Wu T. Jabri B. Murray J. (2017). Open-capsule budesonide for refractory celiac disease. *Am. J. Gastroenterol.* 112 959–967. 10.1038/ajg.2017.71 28323276

[B159] Nakae H. Tsuda A. Matsuoka T. Mine T. Koga Y. (2016). Gastric microbiota in the functional dyspepsia patients treated with probiotic yogurt. *BMJ Open Gastroenterol.* 3:e000109. 10.1136/bmjgast-2016-000109 27752337 PMC5051319

[B160] Nam Y. Kwon S. Lee Y. Jang E. Ahn S. (2018). Relationship between job stress and functional dyspepsia in display manufacturing sector workers: A cross-sectional study. *Ann. Occup. Environ. Med.* 30:62. 10.1186/s40557-018-0274-4 30364417 PMC6194695

[B161] Nan J. Liu J. Mu J. Dun W. Zhang M. Gong Q. (2015). Brain-based correlations between psychological factors and functional dyspepsia. *J. Neurogastroenterol. Motil.* 21 103–110. 10.5056/jnm14096 25540947 PMC4288085

[B162] Nan J. Liu J. Zhang D. Yang Y. Yan X. Yin Q. (2014). Altered intrinsic regional activity and corresponding brain pathways reflect the symptom severity of functional dyspepsia. *Neurogastroenterol. Motil.* 26 660–669. 10.1111/nmo.12311 24467632

[B163] Narotsky D. Green P. Lebwohl B. (2012). Temporal and geographic trends in celiac disease publications: A bibliometric analysis. *Eur. J. Gastroenterol. Hepatol.* 24 1071–1077. 10.1097/MEG.0b013e328355a4ab 22713511

[B164] Nojkov B. Zhou S. Dolan R. Davis E. Appelman H. Guo X. (2020). Evidence of duodenal epithelial barrier impairment and increased pyroptosis in patients with functional dyspepsia on confocal laser endomicroscopy and “ex vivo” mucosa analysis. *Am. J. Gastroenterol.* 115 1891–1901. 10.14309/ajg.0000000000000827 33156108 PMC8409129

[B165] Ohtsu T. Takagi A. Uemura N. Inoue K. Sekino H. Kawashima A. (2017). The ameliorating effect of lactobacillus gasseri OLL2716 on functional dyspepsia in *Helicobacter* pylori-uninfected individuals: A randomized controlled study. *Digestion* 96 92–102. 10.1159/000479000 28768250 PMC5637312

[B166] Pang B. Jiang T. Du Y. Li J. Li B. Hu Y. (2016). Acupuncture for functional dyspepsia: What strength does it have? a systematic review and meta-analysis of randomized controlled trials. *Evid. Based Complement. Alternat. Med.* 2016:3862916. 10.1155/2016/3862916 28119758 PMC5227170

[B167] Paroni Sterbini F. Palladini A. Masucci L. Cannistraci C. Pastorino R. Ianiro G. (2016). Effects of proton pump inhibitors on the gastric mucosa-associated microbiota in dyspeptic patients. *Appl. Environ. Microbiol.* 82 6633–6644. 10.1128/AEM.01437-16 27590821 PMC5086557

[B168] Pasricha P. Grover M. Yates K. Abell T. Bernard C. Koch K. (2021). Functional dyspepsia and gastroparesis in tertiary care are interchangeable syndromes with common clinical and pathologic features. *Gastroenterology* 160 2006–2017. 10.1053/j.gastro.2021.01.230 33548234 PMC8547190

[B169] Pavlidis P. Powell N. Vincent R. Ehrlich D. Bjarnason I. Hayee B. (2015). Systematic review: bile acids and intestinal inflammation-luminal aggressors or regulators of mucosal defence? *Aliment. Pharmacol. Ther.* 42 802–817. 10.1111/apt.13333 26223936

[B170] Pellicano R. Ribaldone D. Fagoonee S. Astegiano M. Saracco G. Mégraud F. (2016). A 2016 panorama of *Helicobacter* pylori infection: Key messages for clinicians. *Panminerva Med.* 58 304–317. 27716738

[B171] Peterson L. Artis D. (2014). Intestinal epithelial cells: regulators of barrier function and immune homeostasis. *Nat. Rev. Immunol.* 14 141–153. 10.1038/nri3608 24566914

[B172] Pleyer C. Bittner H. Locke G. Choung R. Zinsmeister A. Schleck C. (2014). Overdiagnosis of gastro-esophageal reflux disease and underdiagnosis of functional dyspepsia in a USA community. *Neurogastroenterol. Motil.* 26 1163–1171. 10.1111/nmo.12377 24916517

[B173] Potter M. Goodsall T. Walker M. Talley N. (2020). Dual histamine blockade for the treatment of adult functional dyspepsia: A single centre experience. *Gut* 69:966. 10.1136/gutjnl-2019-318412 31040169

[B174] Potter M. Wood N. Walker M. Jones M. Talley N. (2019). Proton pump inhibitors and suppression of duodenal eosinophilia in functional dyspepsia. *Gut* 68 1339–1340. 10.1136/gutjnl-2018-316878 29982192

[B175] Powell N. Walker M. Talley N. (2010). Gastrointestinal eosinophils in health, disease and functional disorders. *Nat. Rev. Gastroenterol. Hepatol.* 7 146–156. 10.1038/nrgastro.2010.5 20125092

[B176] Pritchard A. (1969). Statistical bibliography or bibliometrics. *J. Doc.* 25:348.

[B177] Pulipati P. Sarkar P. Jakkampudi A. Kaila V. Sarkar S. Unnisa M. (2020). The Indian gut microbiota-is it unique? *Indian J. Gastroenterol.* 39 133–140. 10.1007/s12664-020-01037-8 32388710

[B178] Puthanmadhom Narayanan S. Linden D. Peters S. Desai A. Kuwelker S. O’Brien D. (2021). Duodenal mucosal secretory disturbances in functional dyspepsia. *Neurogastroenterol. Motil.* 33:e13955. 10.1111/nmo.13955 32776463 PMC7772227

[B179] Qi R. Shi Z. Weng Y. Yang Y. Zhou Y. Surento W. (2020). Similarity and diversity of spontaneous brain activity in functional dyspepsia subtypes. *Acta Radiol.* 61 927–935. 10.1177/0284185119883391 31684749

[B180] Quigley E. (2017). Prokinetics in the management of functional gastrointestinal disorders. *Curr. Gastroenterol. Rep.* 19:53. 10.1007/s11894-017-0593-6 28887755

[B181] Quigley E. Lacy B. (2013). Overlap of functional dyspepsia and GERD–Diagnostic and treatment implications. *Nat. Rev. Gastroenterol. Hepatol.* 10 175–186. 10.1038/nrgastro.2012.253 23296247

[B182] Rodiño-Janeiro B. Alonso-Cotoner C. Pigrau M. Lobo B. Vicario M. Santos J. (2015). Role of corticotropin-releasing factor in gastrointestinal permeability. *J. Neurogastroenterol. Motil.* 21 33–50. 10.5056/jnm14084 25537677 PMC4288093

[B183] Rodrigues S. P. Van Eck N. J. Waltman L. Jansen F. W. (2014). Mapping patient safety: A large-scale literature review using bibliometric visualisation techniques. *BMJ open* 4:e004468. 10.1136/bmjopen-2013-004468 24625640 PMC3963077

[B184] Rohr M. Narasimhulu C. Rudeski-Rohr T. Parthasarathy S. (2020). Negative effects of a high-fat diet on intestinal permeability: A review. *Adv. Nutr.* 11 77–91. 10.1093/advances/nmz061 31268137 PMC7442371

[B185] Ronkainen J. Aro P. Jones M. Walker M. Agréus L. Andreasson A. (2021). Duodenal eosinophilia and the link to anxiety: A population-based endoscopic study. *Neurogastroenterol. Motil.* 33:e14109. 10.1111/nmo.14109 33687126

[B186] Ronkainen J. Aro P. Walker M. Agréus L. Johansson S. Jones M. (2019). Duodenal eosinophilia is associated with functional dyspepsia and new onset gastro-oesophageal reflux disease. *Aliment. Pharmacol. Ther.* 50 24–32. 10.1111/apt.15308 31107579

[B187] Ropert A. Cherbut C. Rozé C. Le Quellec A. Holst J. Fu-Cheng X. (1996). Colonic fermentation and proximal gastric tone in humans. *Gastroenterology* 111 289–296. 10.1053/gast.1996.v111.pm8690193 8690193

[B188] Rothenberg M. Wen T. Greenberg A. Alpan O. Enav B. Hirano I. (2015). Intravenous anti-IL-13 mAb QAX576 for the treatment of eosinophilic esophagitis. *J. Allergy Clin. Immunol.* 135 500–507. 10.1016/j.jaci.2014.07.049 25226850

[B189] Rupp S. Stengel A. (2022). Bi-directionality of the microbiota-gut-brain axis in patients with functional dyspepsia: Relevance of psychotherapy and probiotics. *Front. Neurosci.* 16:844564. 10.3389/fnins.2022.844564 35295092 PMC8919856

[B190] Salvo-Romero E. Martínez C. Lobo B. Rodiño-Janeiro B. Pigrau M. Sánchez-Chardi A. (2020). Overexpression of corticotropin-releasing factor in intestinal mucosal eosinophils is associated with clinical severity in Diarrhea-Predominant Irritable Bowel Syndrome. *Sci. Rep.* 10:20706. 10.1038/s41598-020-77176-x 33244004 PMC7692489

[B191] Sánchez-Tena M. Martinez-Perez C. Villa-Collar C. Alvarez-Peregrina C. (2022). Long-term effect of contact lens wear: A citation network study. *Cont. Lens Anterior Eye* 45:101527. 10.1016/j.clae.2021.101527 34732300

[B192] Sandhu K. Sherwin E. Schellekens H. Stanton C. Dinan T. Cryan J. (2017). Feeding the microbiota-gut-brain axis: Diet, microbiome, and neuropsychiatry. *Transl. Res.* 179 223–244. 10.1016/j.trsl.2016.10.002 27832936

[B193] Schöffel N. Bendels M. Groneberg D. (2016). Ulcerative colitis: A scientometric approach to the global research output and network. *Eur. J. Intern. Med.* 34 e41–e43. 10.1016/j.ejim.2016.06.019 27373580

[B194] Schöffel N. Brüggmann D. Klingelhöfer D. Bendels M. Groneberg D. (2021). Ulcerative colitis: A critical approach to the global research output employing density-equalizing mapping and scientometric methods. *J. Clin. Gastroenterol.* 55 e19–e26. 10.1097/MCG.0000000000001351 32324679

[B195] Shaidullov I. Sorokina D. Sitdikov F. Hermann A. Abdulkhakov S. Sitdikova G. (2021). Short chain fatty acids and colon motility in a mouse model of irritable bowel syndrome. *BMC Gastroenterol.* 21:37. 10.1186/s12876-021-01613-y 33499840 PMC7836204

[B196] Shanahan F. Ghosh T. O’Toole P. (2021). The healthy microbiome-what is the definition of a healthy gut microbiome? *Gastroenterology* 160 483–494. 10.1053/j.gastro.2020.09.057 33253682

[B197] Shen J. Zhou Z. Cao J. Zhang B. Hu J. Li J. (2022). Biologic therapy for Crohn’s disease over the last 3 decades. *World J. Clin. Cases* 10 594–606. 10.12998/wjcc.v10.i2.594 35097085 PMC8771400

[B198] Shen L. Weber C. Raleigh D. Yu D. Turner J. (2011). Tight junction pore and leak pathways: a dynamic duo. *Annu. Rev. Physiol.* 73 283–309. 10.1146/annurev-physiol-012110-142150 20936941 PMC4655434

[B199] Shimura S. Ishimura N. Mikami H. Okimoto E. Uno G. Tamagawa Y. (2016). Small intestinal bacterial overgrowth in patients with refractory functional gastrointestinal disorders. *J. Neurogastroenterol. Motil.* 22 60–68. 10.5056/jnm15116 26554916 PMC4699722

[B200] Shindo T. Futagami S. Hiratsuka T. Horie A. Hamamoto T. Ueki N. (2009). Comparison of gastric emptying and plasma ghrelin levels in patients with functional dyspepsia and non-erosive reflux disease. *Digestion* 79 65–72. 10.1159/000205740 19246923

[B201] Siddiq K. Akbar H. Khan M. Siddiqui A. Nusrat S. Blay J. (2018). The 100 most influential papers and recent trends in the field of gastrointestinal stromal tumours: A bibliometric analysis. *Cureus* 10:e2311. 10.7759/cureus.2311 29755907 PMC5947978

[B202] Silva Y. Bernardi A. Frozza R. (2020). The role of short-chain fatty acids from gut microbiota in gut-brain communication. *Front. Endocrinol. (Lausanne).* 11:25. 10.3389/fendo.2020.00025 32082260 PMC7005631

[B203] Singh M. Singh V. Schurman J. Friesen C. (2020). Mucosal Th17 cells are increased in pediatric functional dyspepsia associated with chronic gastritis. *Dig. Dis. Sci.* 65 3184–3190. 10.1007/s10620-019-06041-3 31916087

[B204] Small H. (1973). Co-citation in the scientific literature: A new measure of the relationship between documents. *J. Am. Soc. Inf. Sci.* 42 676–684. 10.1002/asi.4630240406 36176388

[B205] Söderholm J. Perdue M. (2001). Stress and gastrointestinal tract. II. Stress and intestinal barrier function. *Am. J. Physiol. Gastrointest. Liver Physiol.* 280 G7–G13. 10.1152/ajpgi.2001.280.1.G7 11123192

[B206] Sohrabi B. Vanani I. R. Jalali S. M. J. Abedin E. (2019). Evaluation of research trends in knowledge management: A hybrid analysis through burst detection and text clustering. *J. Inf. Knowl. Manage.* 18:1950043. 10.1142/S0219649219500436

[B207] Song Y. Pei L. Chen L. Geng H. Yuan M. Xu W. (2020). Electroacupuncture relieves irritable bowel syndrome by regulating IL-18 and gut microbial dysbiosis in a trinitrobenzene sulfonic acid-induced post-inflammatory animal model. *Am. J. Chin. Med.* 48 77–90. 10.1142/S0192415X20500044 31918565

[B208] Sperber A. Bangdiwala S. Drossman D. Ghoshal U. Simren M. Tack J. (2021). Worldwide prevalence and burden of functional gastrointestinal disorders, results of Rome foundation global study. *Gastroenterology* 160 99–114.e3. 10.1053/j.gastro.2020.04.014 32294476

[B209] Stanghellini V. Chan F. Hasler W. Malagelada J. Suzuki H. Tack J. (2016). Gastroduodenal disorders. *Gastroenterology* 150 1380–1392. 10.1053/j.gastro.2016.02.011 27147122

[B210] Stein M. Collins M. Villanueva J. Kushner J. Putnam P. Buckmeier B. (2006). Anti-IL-5 (mepolizumab) therapy for eosinophilic esophagitis. *J. Allergy Clin. Immunol.* 118 1312–1319. 10.1016/j.jaci.2006.09.007 17157662

[B211] Stenfeldt A. Wennerås C. (2004). Danger signals derived from stressed and necrotic epithelial cells activate human eosinophils. *Immunology* 112 605–614. 10.1111/j.1365-2567.2004.01906.x 15270732 PMC1782530

[B212] Straumann A. Conus S. Grzonka P. Kita H. Kephart G. Bussmann C. (2010). Anti-interleukin-5 antibody treatment (mepolizumab) in active eosinophilic oesophagitis: A randomised, placebo-controlled, double-blind trial. *Gut* 59 21–30. 10.1136/gut.2009.178558 19828470

[B213] Sugawara R. Lee E. Jang M. Jeun E. Hong C. Kim J. (2016). Small intestinal eosinophils regulate Th17 cells by producing IL-1 receptor antagonist. *J. Exp. Med.* 213 555–567. 10.1084/jem.20141388 26951334 PMC4821642

[B214] Sun E. Zhang X. Zhao Y. Li J. Sun J. Mu Z. (2021). Beverages containing *Lactobacillus paracasei* LC-37 improved functional dyspepsia through regulation of the intestinal microbiota and their metabolites. *J. Dairy Sci.* 104 6389–6398. 10.3168/jds.2020-19882 33714585

[B215] Šuran D. Blažun Vošner H. Završnik J. Kokol P. Sinkoviè A. Kaniè V. (2022). Lipoprotein(a) in cardiovascular diseases: Insight from a bibliometric study. *Front. Public Health* 10:923797. 10.3389/fpubh.2022.923797 35865239 PMC9294325

[B216] Swiontkowski M. Teague D. Sprague S. Bzovsky S. Heels-Ansdell D. Bhandari M. (2021). Impact of centre volume, surgeon volume, surgeon experience and geographic location on reoperation after intramedullary nailing of tibial shaft fractures. *Can. J. Surg.* 64 E371–E376. 10.1503/cjs.004020 34222771 PMC8410470

[B217] Taché Y. Bonaz B. (2007). Corticotropin-releasing factor receptors and stress-related alterations of gut motor function. *J. Clin. Invest.* 117 33–40. 10.1172/JCI30085 17200704 PMC1716215

[B218] Tack J. Ly H. Carbone F. Vanheel H. Vanuytsel T. Holvoet L. (2016). Efficacy of mirtazapine in patients with functional dyspepsia and weight loss. *Clin. Gastroenterol. Hepatol.* 14 385–392.e4. 10.1016/j.cgh.2015.09.043 26538208

[B219] Takeshita K. (2020). Sharpening the focus: Acupuncture interrupts the brain-gut vicious cycle underlying functional dyspepsia. *Dig. Dis. Sci.* 65 1578–1580. 10.1007/s10620-020-06080-1 32026281

[B220] Taki M. Oshima T. Li M. Sei H. Tozawa K. Tomita T. (2019). Duodenal low-grade inflammation and expression of tight junction proteins in functional dyspepsia. *Neurogastroenterol. Motil.* 31:e13576. 10.1111/nmo.13576 30790378

[B221] Talley N. (2020). What causes functional gastrointestinal disorders? A proposed disease model. *Am. J. Gastroenterol.* 115 41–48. 10.14309/ajg.0000000000000485 31895721

[B222] Talley N. Ford A. (2015). Functional dyspepsia. *N. Engl. J. Med.* 373 1853–1863. 10.1056/NEJMra1501505 26535514

[B223] Talley N. Locke G. Saito Y. Almazar A. Bouras E. Howden C. (2015). Effect of amitriptyline and escitalopram on functional dyspepsia: A multicenter, randomized controlled study. *Gastroenterology* 149 340–349.e2. 10.1053/j.gastro.2015.04.020 25921377 PMC4516571

[B224] Talley N. Walker M. Aro P. Ronkainen J. Storskrubb T. Hindley L. (2007). Non-ulcer dyspepsia and duodenal eosinophilia: An adult endoscopic population-based case-control study. *Clin. Gastroenterol. Hepatol.* 5 1175–1183. 10.1016/j.cgh.2007.05.015 17686660

[B225] Talley N. Walker M. Jones M. Keely S. Koloski N. Cameron R. (2021). Letter: Budesonide for functional dyspepsia with duodenal eosinophilia-randomised, double-blind, placebo-controlled parallel-group trial. *Aliment. Pharmacol. Ther.* 53 1332–1333. 10.1111/apt.16396 34029411

[B226] Tambucci R. Quitadamo P. Ambrosi M. De Angelis P. Angelino G. Stagi S. (2019). Association between obesity/overweight and functional gastrointestinal disorders in children. *J. Pediatr. Gastroenterol. Nutr.* 68 517–520. 10.1097/MPG.0000000000002208 30444836

[B227] Tan N. Gwee K. Tack J. Zhang M. Li Y. Chen M. (2020). Herbal medicine in the treatment of functional gastrointestinal disorders: A systematic review with meta-analysis. *J. Gastroenterol. Hepatol.* 35 544–556. 10.1111/jgh.14905 31674057

[B228] Tan V. Liu K. Lam F. Hung I. Yuen M. Leung W. (2017). Randomised clinical trial: rifaximin versus placebo for the treatment of functional dyspepsia. *Aliment. Pharmacol. Ther.* 45 767–776. 10.1111/apt.13945 28112426

[B229] Tanaka F. Tominaga K. Fujikawa Y. Nagami Y. Kamata N. Yamagami H. (2016). Concentration of glial cell line-derived neurotrophic factor positively correlates with symptoms in functional dyspepsia. *Dig. Dis. Sci.* 61 3478–3485. 10.1007/s10620-016-4329-5 27718082

[B230] Tang L. Zeng Y. Li L. Wang J. Peng D. Zhang T. (2020). Electroacupuncture upregulated ghrelin in rats with functional dyspepsia via AMPK/TSC2/Rheb-mediated mTOR inhibition. *Dig. Dis. Sci.* 65 1689–1699. 10.1007/s10620-019-05960-5 31863340 PMC7225202

[B231] Teitelbaum A. Gareau M. Jury J. Yang P. Perdue M. (2008). Chronic peripheral administration of corticotropin-releasing factor causes colonic barrier dysfunction similar to psychological stress. *Am. J. Physiol. Gastrointest. Liver Physiol.* 295 G452–G459. 10.1152/ajpgi.90210.2008 18635602

[B232] Tejasen C. (2016). Historical bibliometric analysis: A case of the journal of the siam society, 1972–1976. *Proc. Assoc. Inf. Sci. Technol.* 53 1–6. 10.1002/pra2.2016.14505301108

[B233] Tomita T. Oshima T. Miwa H. (2018). New approaches to diagnosis and treatment of functional dyspepsia. *Curr. Gastroenterol. Rep.* 20:55. 10.1007/s11894-018-0663-4 30338390

[B234] Turnbaugh P. Quince C. Faith J. McHardy A. Yatsunenko T. Niazi F. (2010). Organismal, genetic, and transcriptional variation in the deeply sequenced gut microbiomes of identical twins. *Proc. Natl. Acad. Sci. U.S.A.* 107 7503–7508. 10.1073/pnas.1002355107 20363958 PMC2867707

[B235] Tziatzios G. Gkolfakis P. Papanikolaou I. Mathur R. Pimentel M. Damoraki G. (2021). High prevalence of small intestinal bacterial overgrowth among functional dyspepsia patients. *Dig. Dis.* 39 382–390. 10.1159/000511944 33011725

[B236] Urdaneta V. Casadesús J. (2017). Interactions between bacteria and bile salts in the gastrointestinal and hepatobiliary tracts. *Front. Med. (Lausanne)* 4:163. 10.3389/fmed.2017.00163 29043249 PMC5632352

[B237] Van Eck N. J. Waltman L. (2013). VOSviewer manual. *Leiden* 1 1–53.

[B238] Van Houten J. Wessells R. Lujan H. DiCarlo S. (2015). My gut feeling says rest: Increased intestinal permeability contributes to chronic diseases in high-intensity exercisers. *Med. Hypotheses* 85 882–886. 10.1016/j.mehy.2015.09.018 26415977

[B239] Van Oudenhove L. Aziz Q. (2013). The role of psychosocial factors and psychiatric disorders in functional dyspepsia. *Nat. Rev. Gastroenterol. Hepatol.* 10 158–167. 10.1038/nrgastro.2013.10 23358396

[B240] van Zanten G. Knudsen A. Röytiö H. Forssten S. Lawther M. Blennow A. (2012). The effect of selected synbiotics on microbial composition and short-chain fatty acid production in a model system of the human colon. *PLoS One* 7:e47212. 10.1371/journal.pone.0047212 23082149 PMC3474826

[B241] Vanheel H. Farré R. (2013). Changes in gastrointestinal tract function and structure in functional dyspepsia. *Nat. Rev. Gastroenterol. Hepatol.* 10 142–149. 10.1038/nrgastro.2012.255 23318268

[B242] Vanheel H. Carbone F. Valvekens L. Simren M. Tornblom H. Vanuytsel T. (2017). Pathophysiological abnormalities in functional dyspepsia subgroups according to the Rome III criteria. *Am. J. Gastroenterol.* 112 132–140. 10.1038/ajg.2016.499 27958284

[B243] Vanheel H. Vicario M. Beeckmans D. Cocca S. Wauters L. Accarie A. (2020). Duodenal acidification induces gastric relaxation and alters epithelial barrier function by a mast cell independent mechanism. *Sci. Rep.* 10:17448. 10.1038/s41598-020-74491-1 33060783 PMC7562901

[B244] Vanheel H. Vicario M. Vanuytsel T. Van Oudenhove L. Martinez C. Keita Å (2014). Impaired duodenal mucosal integrity and low-grade inflammation in functional dyspepsia. *Gut* 63 262–271. 10.1136/gutjnl-2012-303857 23474421

[B245] Vanuytsel T. van Wanrooy S. Vanheel H. Vanormelingen C. Verschueren S. Houben E. (2014). Psychological stress and corticotropin-releasing hormone increase intestinal permeability in humans by a mast cell-dependent mechanism. *Gut* 63 1293–1299. 10.1136/gutjnl-2013-305690 24153250

[B246] Vasapolli R. Schulz C. Schweden M. Casèn C. Kirubakaran G. Kirste K. (2021). Gut microbiota profiles and the role of anti-CdtB and anti-vinculin antibodies in patients with functional gastrointestinal disorders (FGID). *Eur. J. Clin. Invest.* 51:e13666. 10.1111/eci.13666 34390492

[B247] Vieira E. Leonel A. Sad A. Beltrão N. Costa T. Ferreira T. (2012). Oral administration of sodium butyrate attenuates inflammation and mucosal lesion in experimental acute ulcerative colitis. *J. Nutr. Biochem.* 23 430–436. 10.1016/j.jnutbio.2011.01.007 21658926

[B248] von Wulffen M. Talley N. Hammer J. McMaster J. Rich G. Shah A. (2019). Overlap of irritable bowel syndrome and functional dyspepsia in the clinical setting: prevalence and risk factors. *Dig. Dis. Sci.* 64 480–486. 10.1007/s10620-018-5343-6 30368683

[B249] Walker M. Aggarwal K. Shim L. Bassan M. Kalantar J. Weltman M. (2014). Duodenal eosinophilia and early satiety in functional dyspepsia: Confirmation of a positive association in an Australian cohort. *J. Gastroenterol. Hepatol.* 29 474–479. 10.1111/jgh.12419 24304041

[B250] Walker M. Salehian S. Murray C. Rajendran A. Hoare J. Negus R. (2010). Implications of eosinophilia in the normal duodenal biopsy – An association with allergy and functional dyspepsia. *Aliment. Pharmacol. Ther.* 31 1229–1236. 10.1111/j.1365-2036.2010.04282.x 20222916

[B251] Walker M. Talley N. Prabhakar M. Pennaneac’h C. Aro P. Ronkainen J. (2009). Duodenal mastocytosis, eosinophilia and intraepithelial lymphocytosis as possible disease markers in the irritable bowel syndrome and functional dyspepsia. *Aliment. Pharmacol. Ther.* 29 765–773. 10.1111/j.1365-2036.2009.03937.x 19183150 PMC4070654

[B252] Wallon C. Persborn M. Jönsson M. Wang A. Phan V. Lampinen M. (2011). Eosinophils express muscarinic receptors and corticotropin-releasing factor to disrupt the mucosal barrier in ulcerative colitis. *Gastroenterology* 140 1597–1607. 10.1053/j.gastro.2011.01.042 21277851

[B253] Wallon C. Yang P. Keita A. Ericson A. McKay D. Sherman P. (2008). Corticotropin-releasing hormone (CRH) regulates macromolecular permeability via mast cells in normal human colonic biopsies in vitro. *Gut* 57 50–58. 10.1136/gut.2006.117549 17525093

[B254] Wan M. Orlu-Gul M. Legay H. Tuleu C. (2013). Blinding in pharmacological trials: the devil is in the details. *Arch. Dis. Child.* 98 656–659. 10.1136/archdischild-2013-304037 23898156 PMC3833301

[B255] Wang X. Li X. Ge W. Huang J. Li G. Cong Y. (2015). Quantitative evaluation of duodenal eosinophils and mast cells in adult patients with functional dyspepsia. *Ann. Diagn. Pathol.* 19 50–56. 10.1016/j.anndiagpath.2015.02.001 25735567

[B256] Wauters L. Burns G. Ceulemans M. Walker M. Vanuytsel T. Keely S. (2020a). Duodenal inflammation: An emerging target for functional dyspepsia? *Expert Opin. Ther. Targets* 24 511–523. 10.1080/14728222.2020.1752181 32249629

[B257] Wauters L. Ceulemans M. Vanuytsel T. (2021e). Duodenum at a crossroads: Key integrator of overlapping and psychological symptoms in functional dyspepsia? *Neurogastroenterol. Motil.* 33:e14262. 10.1111/nmo.14262 34561921

[B258] Wauters L. Ceulemans M. Frings D. Lambaerts M. Accarie A. Toth J. (2021a). Proton pump inhibitors reduce duodenal eosinophilia, mast cells, and permeability in patients with functional dyspepsia. *Gastroenterology* 160 1521–1531.e9. 10.1053/j.gastro.2020.12.016 33346007

[B259] Wauters L. Dickman R. Drug V. Mulak A. Serra J. Enck P. (2021b). United european gastroenterology (UEG) and european society for neurogastroenterology and motility (ESNM) consensus on functional dyspepsia. *Neurogastroenterol. Motil.* 33:e14238. 10.1111/nmo.14238 34399024

[B260] Wauters L. Slaets H. De Paepe K. Ceulemans M. Wetzels S. Geboers K. (2021c). Efficacy and safety of spore-forming probiotics in the treatment of functional dyspepsia: a pilot randomised, double-blind, placebo-controlled trial. *Lancet Gastroenterol. Hepatol.* 6 784–792. 10.1016/S2468-1253(21)00226-0 34358486

[B261] Wauters L. Talley N. Walker M. Tack J. Vanuytsel T. (2020b). Novel concepts in the pathophysiology and treatment of functional dyspepsia. *Gut* 69 591–600. 10.1136/gutjnl-2019-318536 31784469

[B262] Wauters L. Tito R. Ceulemans M. Lambaerts M. Accarie A. Rymenans L. (2021d). Duodenal dysbiosis and relation to the efficacy of proton pump inhibitors in functional dyspepsia. *Int. J. Mol. Sci.* 22:13609. 10.3390/ijms222413609 34948413 PMC8708077

[B263] Weber R. P. (1990). *Basic content analysis*, Vol. 49. Thousand Oaks, CA: Sage. 10.4135/9781412983488

[B264] Wechsler M. Munitz A. Ackerman S. Drake M. Jackson D. Wardlaw A. (2021). Eosinophils in health and disease: A state-of-the-art review. *Mayo Clin. Proc.* 96 2694–2707. 10.1016/j.mayocp.2021.04.025 34538424

[B265] Wei L. Singh R. Ro S. Ghoshal U. (2021). Gut microbiota dysbiosis in functional gastrointestinal disorders: Underpinning the symptoms and pathophysiology. *JGH Open* 5 976–987. 10.1002/jgh3.12528 34584964 PMC8454481

[B266] Weller P. Spencer L. (2017). Functions of tissue-resident eosinophils. *Nat. Rev. Immunol.* 17 746–760. 10.1038/nri.2017.95 28891557 PMC5783317

[B267] Wilhelm S. Rjater R. Kale-Pradhan P. (2013). Perils and pitfalls of long-term effects of proton pump inhibitors. *Expert Rev. Clin. Pharmacol.* 6 443–451. 10.1586/17512433.2013.811206 23927671

[B268] Williamson K. Johanson G. (Eds.) (2017). *Research methods: Information, systems, and contexts.* Cambridge: Chandos Publishing.

[B269] Wu P. Xiong S. Zhong P. Yang W. Chen M. Tang T. (2022). Global trends in research on irritable bowel syndrome and the brain-gut axis: Bibliometrics and visualization analysis. *Front. Pharmacol.* 13:956204. 10.3389/fphar.2022.956204 36160395 PMC9493189

[B270] Xiang C. Wang Y. Liu H. (2017). A scientometrics review on nonpoint source pollution research. *Ecol. Eng.* 99 400–408. 10.1016/j.ecoleng.2016.11.028

[B271] Xiong J. Fu Y. Qiu J. Liao W. Luo L. Chen S. (2022). Global research trends of immunotherapy and biotherapy for inflammatory bowel disease: A bibliometric analysis from 2002 to 2021. *Biomed. Eng. Online* 21:42. 10.1186/s12938-022-01011-9 35761289 PMC9238098

[B272] Xiong L. Gong X. Siah K. Pratap N. Ghoshal U. Abdullah M. (2017). Rome foundation Asian working team report: Real world treatment experience of Asian patients with functional bowel disorders. *J. Gastroenterol. Hepatol.* 32 1450–1456. 10.1111/jgh.13730 28084664

[B273] Xu D. Gao J. Gillilland M. Wu X. Song I. Kao J. (2014). Rifaximin alters intestinal bacteria and prevents stress-induced gut inflammation and visceral hyperalgesia in rats. *Gastroenterology* 146 484–496.e4. 10.1053/j.gastro.2013.10.026 24161699 PMC3939606

[B274] Xu P. Lv T. Dong S. Cui Z. Luo X. Jia B. (2022). Association between intestinal microbiome and inflammatory bowel disease: Insights from bibliometric analysis. *Comput. Struct. Biotechnol. J.* 20 1716–1725. 10.1016/j.csbj.2022.04.006 35495114 PMC9019919

[B275] Xu Z. Wang X. Wang X. Skare M. (2021). A comprehensive bibliometric analysis of entrepreneurship and crisis literature published from 1984 to 2020. *J. Bus. Res.* 135 304–318. 10.1016/j.jbusres.2021.06.051

[B276] Yagi T. Asakawa A. Ueda H. Miyawaki S. Inui A. (2013). The role of ghrelin in patients with functional dyspepsia and its potential clinical relevance (Review). *Int. J. Mol. Med.* 32 523–531. 10.3892/ijmm.2013.1418 23778458

[B277] Yang N. Tan C. Lin L. Su X. Li Y. Qi L. (2022). Potential mechanisms of acupuncture for functional dyspepsia based on pathophysiology. *Front. Neurosci.* 15:781215. 10.3389/fnins.2021.781215 35145373 PMC8822151

[B278] Ye Y. Wang X. Zheng Y. Yang J. Yang N. Shi G. (2018). Choosing an animal model for the study of functional dyspepsia. *Can. J. Gastroenterol. Hepatol.* 2018:1531958. 10.1155/2018/1531958 29623262 PMC5830275

[B279] Yeung A. W. K. (2019). Comparison between Scopus, Web of Science, PubMed and publishers for mislabelled review papers. *Curr. Sci.* 116 1909–1914. 10.18520/cs/v116/i11/1909-1914

[B280] Yoon J. Ko S. Park J. Cha J. (2022). Complementary and alternative medicine for functional dyspepsia: An Asian perspective. *Medicine (Baltimore)* 101:e30077. 10.1097/MD.0000000000030077 36107498 PMC9439791

[B281] Yuan H. Li Z. Zhang Y. Li X. Li F. Li Y. (2015). Anxiety and depression are associated with increased counts and degranulation of duodenal mast cells in functional dyspepsia. *Int. J. Clin. Exp. Med.* 8 8010–8014. 26221363 PMC4509308

[B282] Yuan X. Chang C. Chen X. Li K. (2021). Emerging trends and focus of human gastrointestinal microbiome research from 2010-2021: A visualized study. *J. Transl. Med.* 19:327. 10.1186/s12967-021-03009-8 34332587 PMC8325541

[B283] Zala A. Walker M. Talley N. (2015). Emerging drugs for functional dyspepsia. *Expert Opin. Emerg. Drugs* 20 221–233. 10.1517/14728214.2015.1009827 25645940

[B284] Železnik D. Blažun Vošner H. Kokol P. (2017). A bibliometric analysis of the journal of advanced nursing, 1976-2015. *J. Adv. Nurs.* 73 2407–2419. 10.1111/jan.13296 28295539

[B285] Zeng F. Qin W. Liang F. Liu J. Tang Y. Liu X. (2011). Abnormal resting brain activity in patients with functional dyspepsia is related to symptom severity. *Gastroenterology* 141 499–506. 10.1053/j.gastro.2011.05.003 21684280

[B286] Zeng F. Qin W. Ma T. Sun J. Tang Y. Yuan K. (2012). Influence of acupuncture treatment on cerebral activity in functional dyspepsia patients and its relationship with efficacy. *Am. J. Gastroenterol.* 107 1236–1247. 10.1038/ajg.2012.53 22641307

[B287] Zeng F. Song W. Liu X. Xie H. Tang Y. Shan B. (2009). Brain areas involved in acupuncture treatment on functional dyspepsia patients: A PET-CT study. *Neurosci. Lett.* 456 6–10. 10.1016/j.neulet.2009.03.080 19429123

[B288] Zhang J. Lin M. (2022). A comprehensive bibliometric analysis of Apache Hadoop from 2008 to 2020. *Int. J. Intell. Comput. Cybern.* 10.1108/IJICC-01-2022-0004 [Epub ahead of print].

[B289] Zhang J. Liu Y. Huang X. Chen Y. Hu L. Lan K. (2020). Efficacy comparison of different acupuncture treatments for functional dyspepsia: A systematic review with network meta-analysis. *Evid. Based Complement. Alternat. Med.* 2020:3872919. 10.1155/2020/3872919 32256643 PMC7106911

[B290] Zhang L. Ling J. Lin M. (2022a). Artificial intelligence in renewable energy: A comprehensive bibliometric analysis. *Energy Rep.* 8 14072–14088. 10.1016/j.egyr.2022.10.347

[B291] Zhang L. Ling J. Lin M. (2023). Carbon neutrality: A comprehensive bibliometric analysis. *Environ. Sci. Pollut. Res. Int.* 10.1007/s11356-023-25797-w [Epub ahead of print]. 36800084

[B292] Zhang S. Liu Y. Li S. Ye F. Foreman R. Chen J. (2020). Effects of electroacupuncture on stress-induced gastric dysrhythmia and mechanisms involving autonomic and central nervous systems in functional dyspepsia. *Am. J. Physiol. Regul. Integr. Comp. Physiol.* 319 R106–R113. 10.1152/ajpregu.00256.2019 32493036 PMC8424570

[B293] Zhang T. Ma X. Tian W. Zhang J. Wei Y. Zhang B. (2022b). Global research trends in irritable bowel syndrome: A bibliometric and visualized study. *Front. Med. (Lausanne)* 9:922063. 10.3389/fmed.2022.922063 35833106 PMC9271748

[B294] Zhang T. Zhang B. Tian W. Ma X. Wang F. Wang P. (2022c). A Bibliometric analysis of atrophic gastritis from 2011 to 2021. *Front. Med. (Lausanne)* 9:843395. 10.3389/fmed.2022.843395 35252276 PMC8891522

[B295] Zheng P. Feng B. Oluwole C. Struiksma S. Chen X. Li P. (2009). Psychological stress induces eosinophils to produce corticotrophin releasing hormone in the intestine. *Gut* 58 1473–1479. 10.1136/gut.2009.181701 19651632

[B296] Zhong L. Shanahan E. Raj A. Koloski N. Fletcher L. Morrison M. (2017). Dyspepsia and the microbiome: Time to focus on the small intestine. *Gut* 66 1168–1169. 10.1136/gutjnl-2016-312574 27489239

[B297] Zhong M. Lin M. (2022). Bibliometric analysis for economy in COVID-19 pandemic. *Heliyon* 8:e10757. 10.1016/j.heliyon.2022.e10757 36185135 PMC9509534

[B298] Zhou H. Liang H. Li Z. Xiang H. Liu W. Li J. (2013). Vagus nerve stimulation attenuates intestinal epithelial tight junctions disruption in endotoxemic mice through α7 nicotinic acetylcholine receptors. *Shock* 40 144–151. 10.1097/SHK.0b013e318299e9c0 23860583

[B299] Zhou W. Su J. Zhang H. (2016). Efficacy and safety of acupuncture for the treatment of functional dyspepsia: Meta-analysis. *J. Altern. Complement. Med.* 22 380–389. 10.1089/acm.2014.0400 27028618

[B300] Ziko I. Sominsky L. De Luca S. Lelngei F. Spencer S. (2018). Acylated ghrelin suppresses the cytokine response to lipopolysaccharide and does so independently of the hypothalamic-pituitary-adrenal axis. *Brain Behav. Immun.* 74 86–95. 10.1016/j.bbi.2018.07.011 30009998

[B301] Zimmermann N. Hershey G. Foster P. Rothenberg M. (2003). Chemokines in asthma: Cooperative interaction between chemokines and IL-13. *J. Allergy Clin. Immunol.* 111 227–42; quiz243. 10.1067/mai.2003.139 12589338

[B302] Zyoud S. Smale S. Waring W. Sweileh W. Al-Jabi S. (2021). Global research trends in the microbiome related to irritable bowel syndrome: A bibliometric and visualized study. *World J. Gastroenterol.* 27 1341–1353. 10.3748/wjg.v27.i13.1341 33833487 PMC8015301

[B303] Zyoud S. Smale S. Waring W. Sweileh W. Al-Jabi S. (2019). Global research trends in microbiome-gut-brain axis during 2009-2018: A bibliometric and visualized study. *BMC Gastroenterol.* 19:158. 10.1186/s12876-019-1076-z 31470803 PMC6716890

