# Supplementary material for: Research trends in the field of the gut-brain interaction: Functional dyspepsia in the spotlight – An integrated bibliometric and science mapping approach
Source: Front Neurosci. 2023 Mar 8;17:1109510. doi: 10.3389/fnins.2023.1109510 (PMC10035075; doi:10.3389/fnins.2023.1109510)

Supplementary Material

Research trends in the field of the gut-brain interaction: functional dyspepsia in the spotlight. An integrated bibliometric and science mapping approach

Tai Zhang1,2,3, Beihua Zhang1,2,3, Xiangxue Ma1,2,3, Jiaqi Zhang1,2,3, Yuchen Wei1,2,3, Fengyun Wang1,2,3*, and Xudong Tang3*

*** Correspondence:** wfy811@163.com (F.W.); txdly@sina.com (X.T.)

# Supplementary Data

Search strategy in Science Citation Index Expanded of the Web of Science Core Collection of Clarivate Analytics

((TI=((“indigestion*” AND (intestin* OR digest* OR gastr* OR gut OR epigastr* OR stomach*)) OR dyspep* OR asepsy OR asepsia OR (“epigastric pain syndrome”) OR ((disturbance* OR disorder* OR difficult* OR dysfunction* OR disease* OR impair* OR condition* OR abnormal* OR illness* OR patholog* OR discomfort* OR damage* OR injur* OR irritab* OR pain* OR distress* OR burning) AND postprandial*))) OR AK=((“indigestion*” AND (intestin* OR digest* OR gastr* OR gut OR epigastr* OR stomach*)) OR dyspep* OR asepsy OR asepsia OR (“epigastric pain syndrome”) OR ((disturbance* OR disorder* OR difficult* OR dysfunction* OR disease* OR impair* OR condition* OR abnormal* OR illness* OR patholog* OR discomfort* OR damage* OR injur* OR irritab* OR pain* OR distress* OR burning) AND postprandial*))) AND DT=(Article OR Review). Here, a changeable string of characters was represented with an asterisk (*), which serves as a wildcard.

Timespan: 1 January, 2006 to December 31, 2021.

**Supplementary Figure 1**

*H. pylori* infection (red cluster)


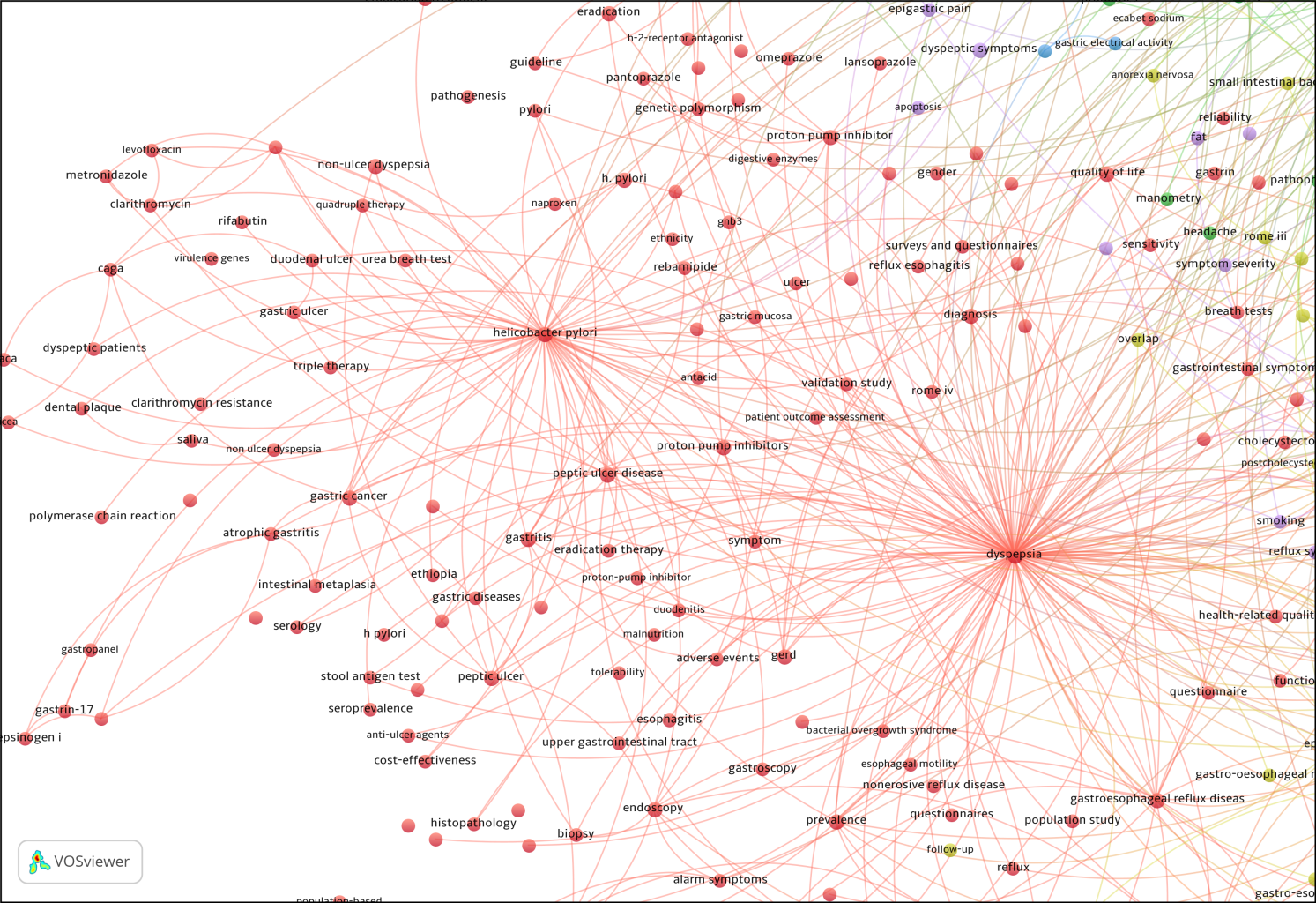


**Supplementary Figure 2**

Pathophysiological mechanisms of FD (green cluster)


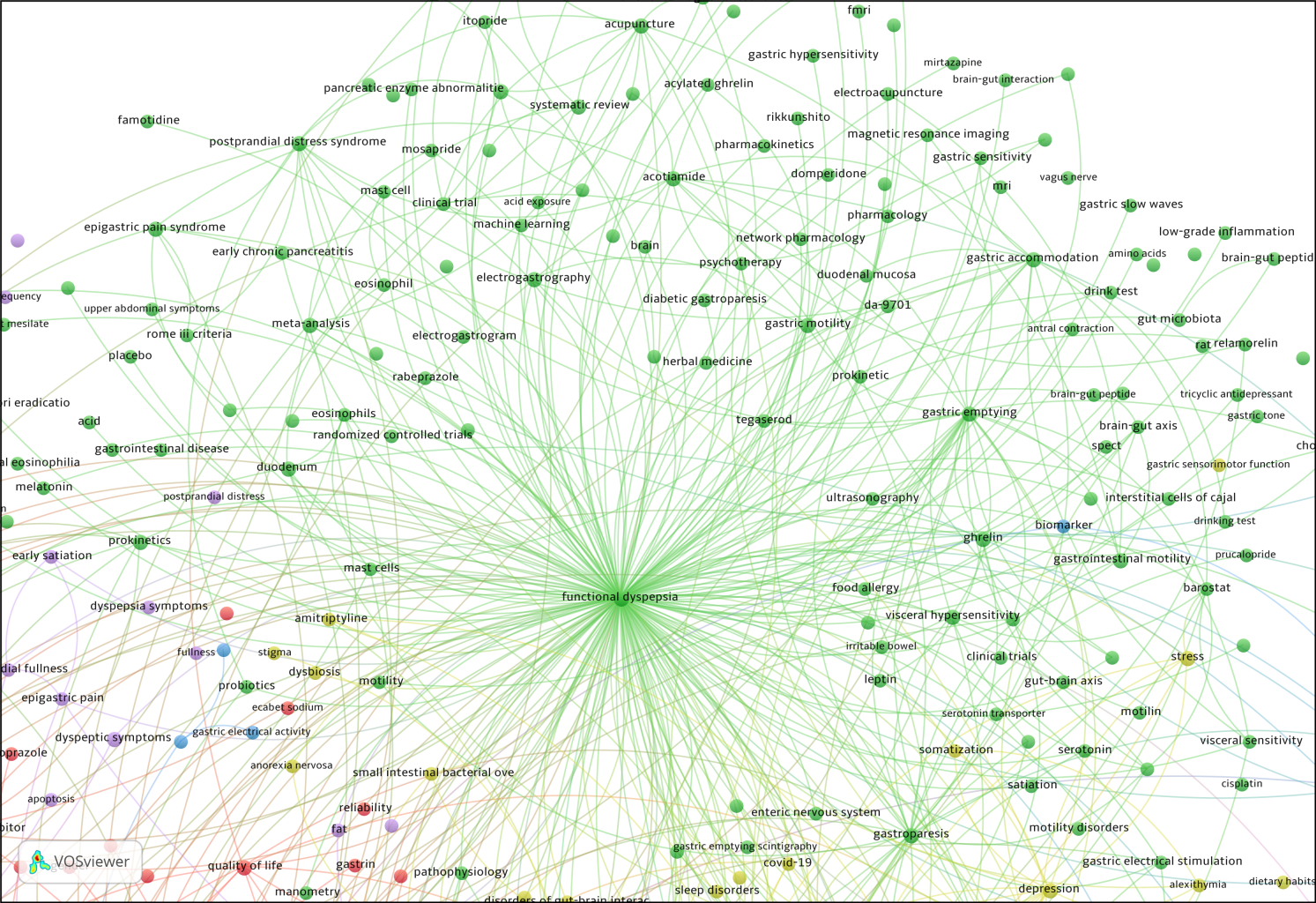


**Supplementary Figure 3**

Extraintestinal comorbidities and overlap syndromes associated with FD (yellow cluster); Herbal medicine in FD (pink cluster)


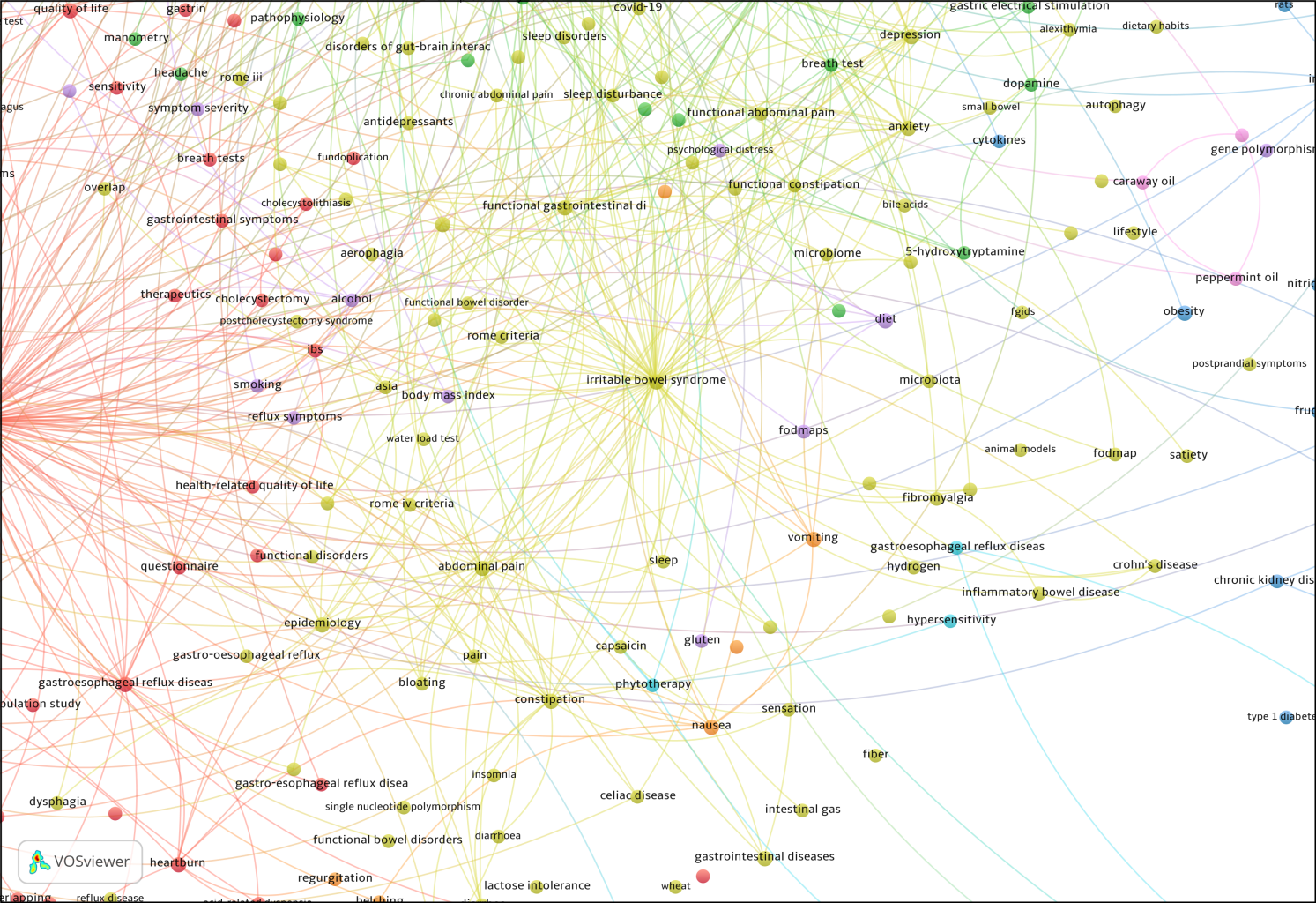


**Supplementary Figure 4**

Herbal medicine in FD (light blue cluster)

**
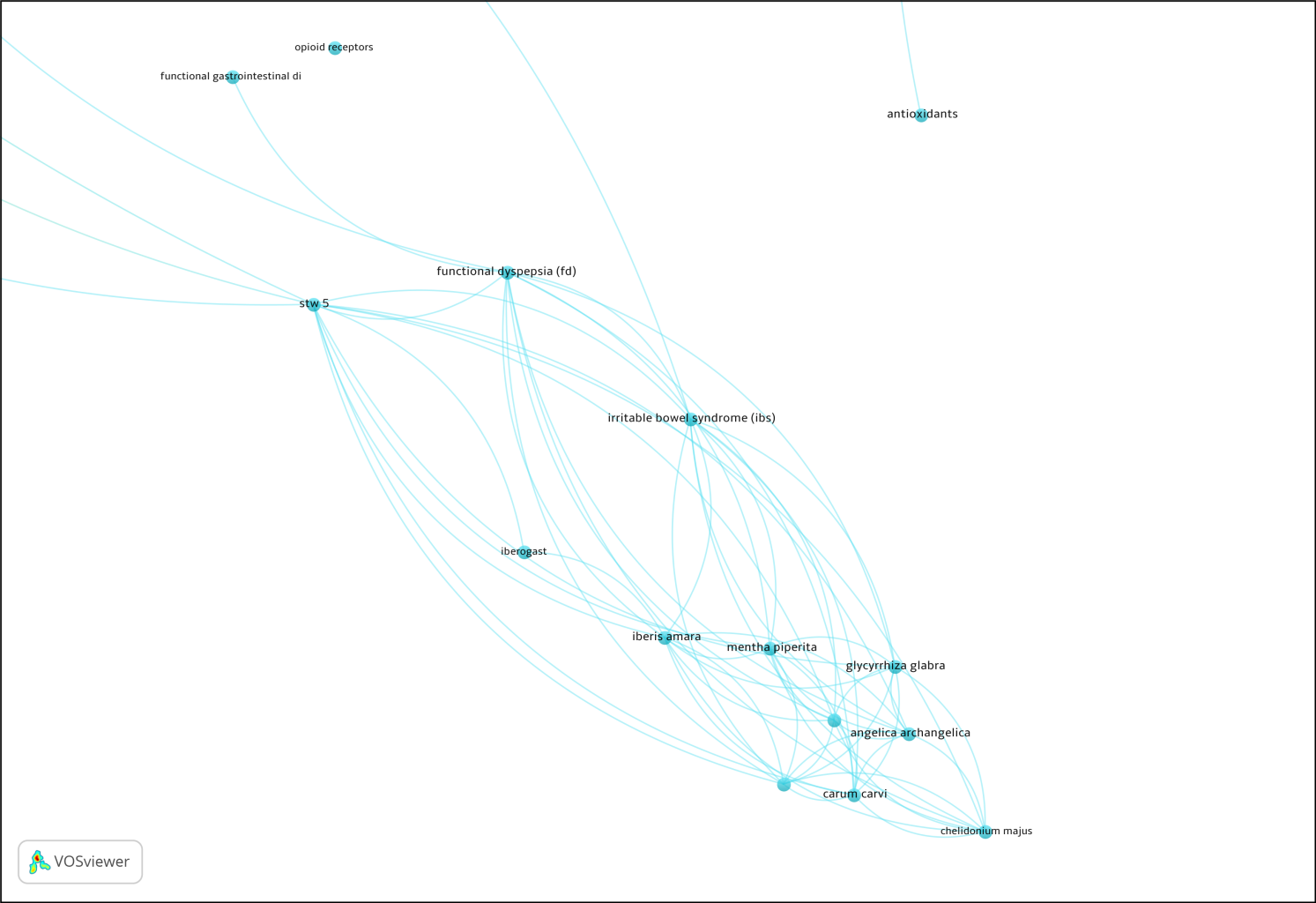
**

**Supplementary Figure 5**

Diabetic gastroparesis (dark blue cluster)


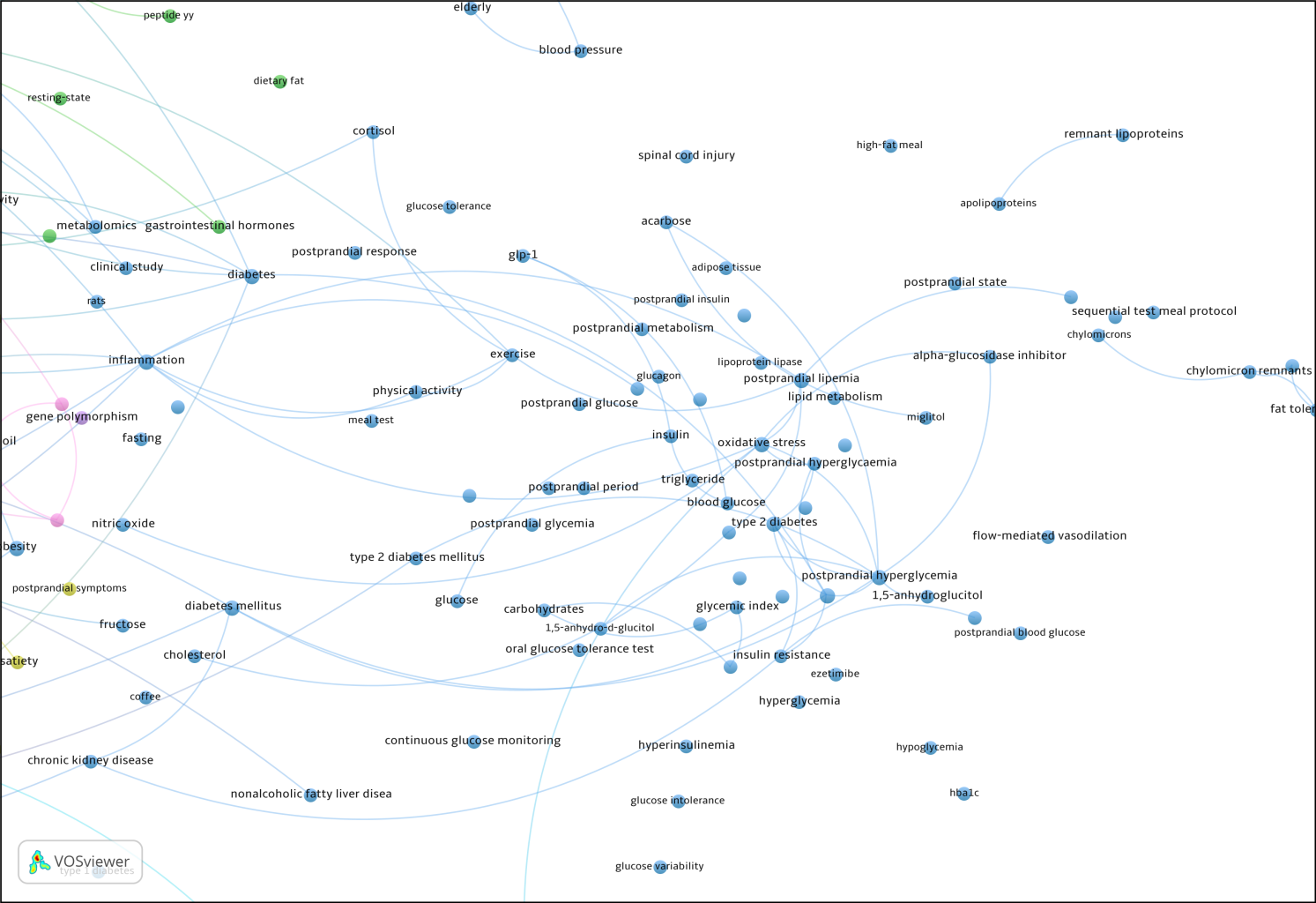


**Supplementary Figure 6**

Dietary factors in FD (purple cluster)


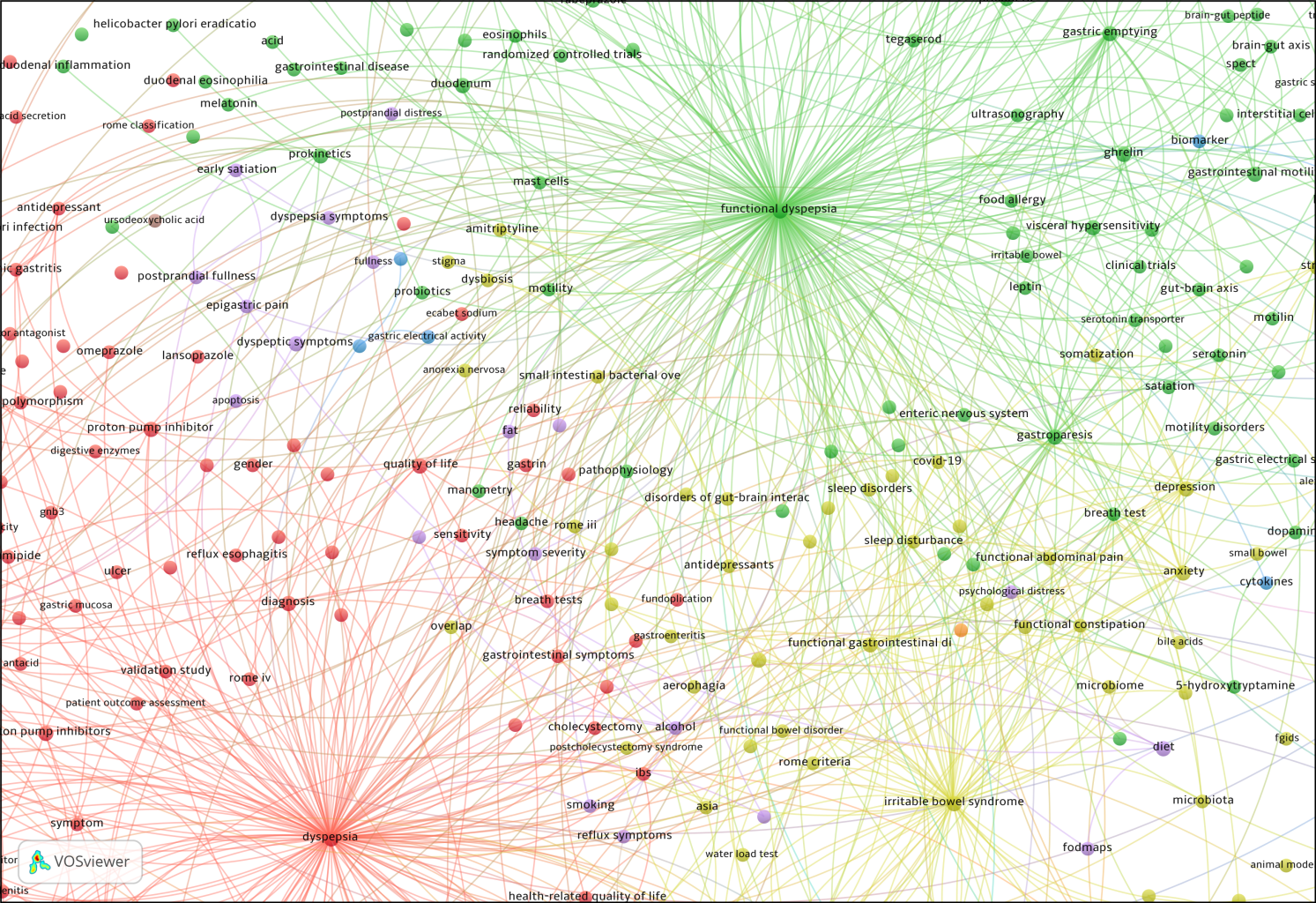


**Supplementary Figure 7**

The clustered scientific landscape for the period 2017-2019


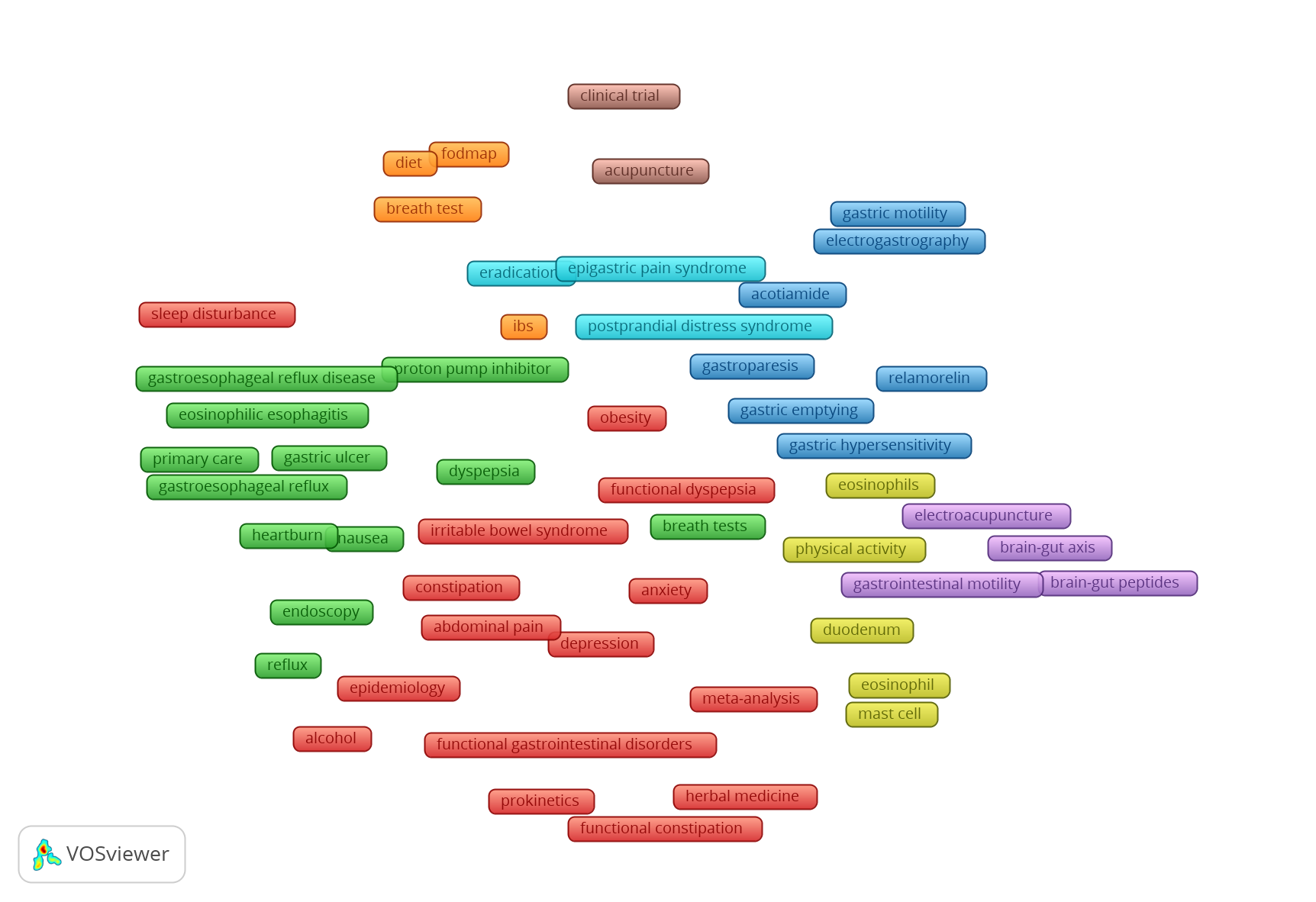


**Supplementary Figure 8**

The clustered scientific landscape for the period 2020-2022


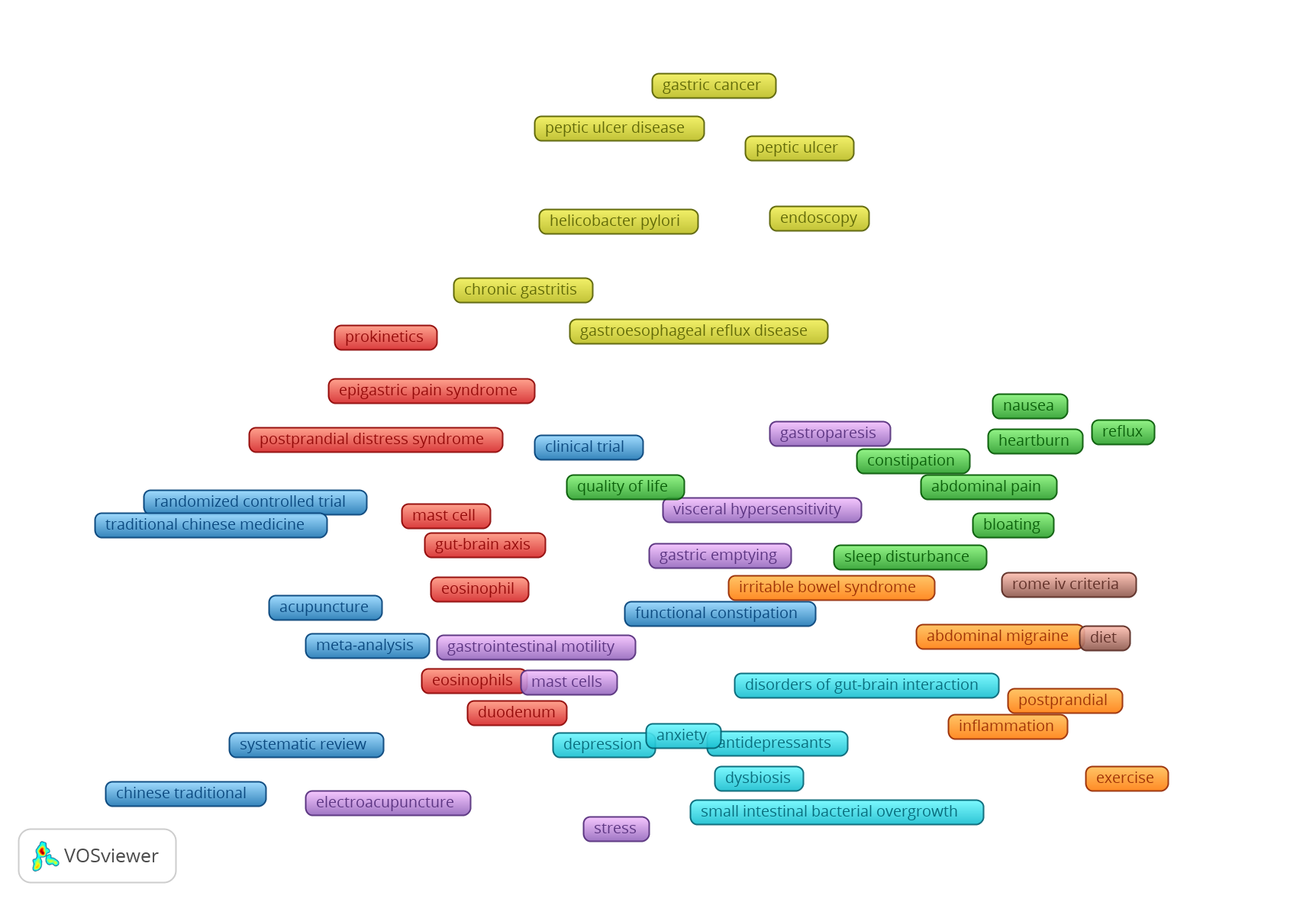

Supplement: Supplementary file 1 [file Data_Sheet_1.docx]
